# *KCNQ1* disruption alters human neuronal development through mitochondrial and insulin signalling pathways

**DOI:** 10.1038/s42003-026-11111-0

**Published:** 2026-10-13

**Authors:** Dorothea Schall, Baran E. Güler, Siamand Alibrahim, Mahmudul Hasan, Naeem Ahmad, Joanna Widomska, Claudio Acuna, Mairéad Sullivan, Ralph Röth, Chang Liu, Linda Blaier, Katalin Vincze, Karin Burau, Simone Macri, Daniela Mauceri, Marcin Luzarowski, Ágota Apáti, János M. Réthelyi, Geert Poelmans, Gudrun A. Rappold, Barbara Franke, Jeffrey C. Glennon, Simone Berkel

**Affiliations:** 1 Institute of Human Genetics, University Hospital Heidelberg, Heidelberg, Germany; 2 Pharmaceutical Biotechnology, Department of Pharmacological and Biomolecular Sciences, University of Milan La Statale, Milan, Italy; 3 National Institute for Biotechnology and Genetic Engineering-College, Pakistan Institute of Engineering and Applied Sciences (NIBGE-C, PIEAS), Jhang Road, Faisalabad, Pakistan; 4 Department of Medical Neuroscience, Radboud University Medical Center, Nijmegen, The Netherlands; 5 Donders Institute for Brain, Cognition and Behaviour, Radboud University, Nijmegen, The Netherlands; 6 Chica and Heinz Schaller Foundation, Institute of Anatomy and Cell Biology, Heidelberg University, Heidelberg, Germany; 7 Conway Institute of Biomolecular and Biomedical Research, School of Medicine, University College Dublin, Dublin, Ireland; 8 nCounter Core Facility, Institute of Human Genetics, Heidelberg University Hospital, Heidelberg, Germany; 9 Department of Psychiatry and Psychotherapy, Semmelweis University, Budapest, Hungary; 10 HUN-REN, Research Centre for Natural Sciences, Budapest, Hungary; 11 Centre for Behavioural Sciences and Mental Health, Istituto Superiore di Sanità, Rome, 00161 Italy; 12 Department of Neurobiology, Interdisciplinary Centre for Neurosciences (IZN), Heidelberg University, Heidelberg, Germany; 13 Institute of Anatomy and Cell Biology, Dept. Molecular and Cellular Neuroscience, University of Marburg, Marburg, Germany; 14 Core Facility for Mass Spectrometry and Proteomics, Center for Molecular Biology of Heidelberg University (ZMBH), DKFZ-ZMBH Alliance, 69120 Heidelberg, Germany; 15 Department of Human Genetics, Radboud University Medical Center, Nijmegen, The Netherlands; 16 Institute of Human Genetics, Heidelberg University, Heidelberg, Germany

**Keywords:** Ion channels in the nervous system, Molecular neuroscience

## Abstract

*KCNQ1* encodes a voltage-gated potassium channel implicated in peripheral and neurological disorders, but its role in human neurodevelopment remains unclear. We generated *KCNQ1* knockout (KO) human induced pluripotent stem cells and differentiated them into neural stem cells (NSCs) and cortical neurons. *KCNQ1*-deficient NSCs show impaired neurite outgrowth, linked to reduced cell adhesion and disrupted neural cell adhesion molecule signalling, a phenotype reproduced by pharmacological KCNQ1 inhibition. Multi-omics and follow-up analyses reveal mitochondrial dysfunction, including reduced mitochondrial copy number and ATP synthase expression. KO cells also show impaired insulin signalling, with diminished insulin receptor gene expression and altered RAS-MAPK and PI3K-AKT pathways. In differentiated neuronal cultures, *KCNQ1* loss decreases synaptic activity and maintains an immature transcriptional profile. Together, these findings identify a role for KCNQ1 in regulating cell adhesion, mitochondrial function, insulin signalling, and neuronal maturation, expanding our understanding of processes that may contribute to neurological manifestations associated with *KCNQ1*-related disorders.

## Introduction

KCNQ1 is the pore-forming alpha subunit of a voltage-gated potassium channel complex. It interacts with KCNE family members, which act as beta subunits, to assemble the functional Kv7.1 potassium channel^1,2^. This channel generates a slow delayed rectifier current^3^.

KCNQ1 is ubiquitously expressed in humans, though its function varies in different tissues^2^. In the heart, it is responsible for the main repolarizing potassium current, and for the regulation of the cardiac action potential duration^3^. In peripheral organs such as the stomach, intestines, kidney, thyroid gland, and the respiratory system, KCNQ1 is expressed in epithelial cells. Here, it facilitates the transport of ions, hormones, and water, maintaining potassium homeostasis^2^. In the pancreas, it modulates insulin secretion from beta cells by regulating potassium channel currents^4^. KCNQ1 is also expressed in multiple regions of the developing and adult brain in mice and humans. In neuronal tissues, it interacts with KCNE1 and is hypothesized to influence neuronal excitability^5^. Evidence for neuronal expression remains limited, although neuronal localization has been reported in two studies^5,6^. In addition, active translation of *Kcnq1* transcripts was demonstrated in developing rat prefrontal neurons, where pharmacological and molecular experiments implicated Kcnq1 in the regulation of intrinsic neuronal excitability^7^. Kcnq1 expression has also been detected in rat brain microvessels and primary endothelial cells, suggesting a potential role in blood-brain barrier permeability^8^. However, the precise function of KCNQ1 in the brain and neurons remains largely unknown.

*KCNQ1* is an imprinted gene, with only the maternal transcript expressed in most human tissues. The paternal transcript is silenced by the non-coding *KCNQ1 overlapping transcript 1* (*KCNQ1OT1*) which is located in antisense orientation within intron 10 of the *KCNQ1* gene^9^. *KCNQ1* has been linked to various diseases, primarily those affecting the heart, such as familial atrial fibrillation^10^, long^11^ and short^12^ QT syndrome, Romano-Ward syndrome^13^, and Jervell and Lange-Nielsen syndrome; the latter presents with deafness^4^. Additionally, *KCNQ1* is recognized as a susceptibility gene for diabetes mellitus type 2 (DM2)^14,15^ in genome-wide association studies (GWAS). Genetic variants in *KCNQ1* have been identified in patients with epilepsy and can increase risk for sudden unexplained death^5^. Moreover, the integration of GWAS findings on obsessive compulsive disorder (OCD) has highlighted insulin-related signalling as a key pathway in OCD etiology and identified KCNQ1 as a crucial molecule in this process^16,17^.

Notably, comorbidities exist among the various diseases in which *KCNQ1* is involved. Genetic variants in *KCNQ1* have been identified in patients with long QT syndrome (LQTS), who also experience epilepsy^18–20^. Individuals with LQTS are at increased risk of developing DM2, hearing loss, epilepsy, and psychiatric disorders compared to the general population^21,22^. Patients with DM2 were found to exhibit mild reductions in cognitive function, and an elevated risk for dementia (50–100%) compared to matched non-diabetic controls^23–25^. They performed worse on tests assessing verbal and visual memory, attention, processing speed, executive function, and motor control^23,25^. DM2 is caused by dysfunctional insulin signalling leading to insulin resistance, and is linked to multiple co-morbidities including LQTS^21^, epilepsy^26^, neuropsychiatric^27,28^ and neurodegenerative disorders^25^. Recent studies provide first evidence for a shared genetic etiology of these multi-morbid conditions, with mounting evidence pointing to disturbed insulin signalling as a common mechanism^29,30^. Given *KCNQ1*´s involvement in LQTS, DM2, epilepsy, and OCD, it may also play a role in comorbidities between diseases of peripheral organs and the central nervous system. As KCNQ1 regulates insulin secretion in pancreatic cells^4^, and contributes to DM2 and related conditions, we hypothesize that it may also influence insulin signalling in the brain and impacts disease comorbidities.

In this study, we investigated KCNQ1 in human neuronal stem cells (NSCs) and cortical neurons to better understand its functional role, and to unravel its impact on insulin signalling. The majority of pathogenic variants in *KCNQ1* are loss-of-function; therefore, genetic deletion of *KCNQ1* helps to assess its physiological role, and to elucidate disease mechanisms. To achieve this, we generated knockouts (KOs) of *KCNQ1* in human induced pluripotent stem cells (iPSCs) that were subsequently differentiated into NSCs and cortical neurons. The loss of *KCNQ1* impaired early neuronal differentiation by reducing neurite outgrowth and affected neuronal function. Transcriptome and proteome analyses revealed disruptions in gene transcription, protein translation, mitochondrial function and insulin signalling, involving key downstream effectors such as RAS, mitogen-activated protein kinase (MAPK, also known as “extracellular signal-regulated kinase” (ERK)), phosphatidylinositol 3-kinase (PI3K), and Akt serine/threonine kinase (AKT). Overall, our findings demonstrate a role for KCNQ1 in neuronal development and function.

## Results

### Generation and differentiation of *KCNQ1*-KO lines

**Fig. 1:**
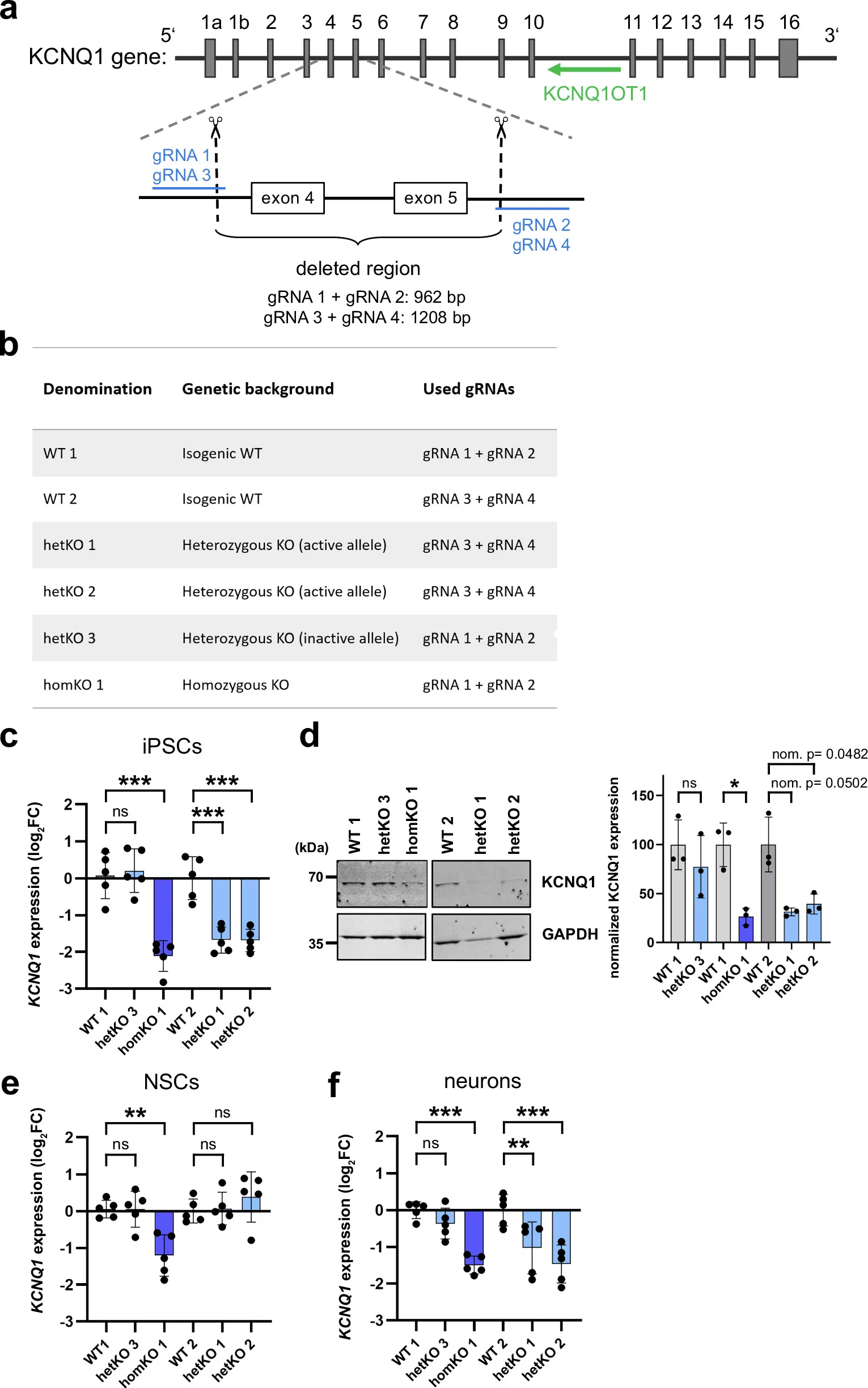
Generation of *KCNQ1*-KO cell lines and relative *KCNQ1* expression in iPSCs, NSCs and neurons. **a** Illustration of the *KCNQ1* gene and the induced deletion. Exons 4 and 5 were deleted by using two gRNAs flanking both exons. KCNQ1OT1 is the non-coding *KCNQ1* overlapping transcript 1 in antisense orientation. **b** List of generated *KCNQ1*-KO cell lines and isogenic controls. **c** KCNQ1 expression on RNA level in iPSCs. KO lines were compared to their respective WT control (treated with the same gRNA, see 1b) and expression changes were calculated as log2fold change (log2FC). Data are presented as mean ± SD (n = 5 biological replicates). One-way ANOVA with Bonferroni-adjusted post hoc testing. ANOVA: *p* < 0.0001, F = 23.34, post hoc test: hetKO 3: *p* > 0.999; homKO 1, hetKO 1, hetKO 2: *p* < 0.001. **d** KCNQ1 protein expression in iPSCs assessed by Western blot (left, representative blot; right, quantification) normalized to GAPDH. HomKO 1 and hetKO 3 were compared to WT 1 on separate membranes using independent WT 1 replicates; therefore, WT 1 values are membrane-specific and not directly comparable across panels. Statistical analyses were restricted to within-membrane comparisons using a two-tailed Welch’s t-tests (n = 3 biological replicates). Nominal *p*-values: hetKO 3: *p* = 0.3319, homKO 1: *p* = 0.0076, hetKO 1: *p* = 0.0482, hetKO 2: *p* = 0.0502; adjusted p-values after Bonferroni correction: hetKO 3: *p* > 0.999, homKO 1: *p* = 0.0304, hetKO 1: *p* = 0.1928, hetKO 2: *p* = 0.2008, values were normalized to mean of the respective WT control which was set to 100. Nom. – nominal *p*-value. **e, f** KCNQ1 expression on RNA level in NSCs (e), and neurons (f) shown as log2FC relative to the corresponding WT controls (see b). Data are presented as mean ± SD (n = 5 biological replicates). One-way ANOVA with Bonferroni-adjusted post hoc testing. **e** ANOVA: *p* < 0.0005, F = 6.71, post hoc test: hetKO 3: *p* > 0.999, homKO 1: *p* < 0.0014, hetKO 1: *p* > 0.999, hetKO 2: *p* = 0.8789. **f** ANOVA: *p* < 0.0001, F = 11.70, post hoc test: hetKO 3: *p* = 0.8663, homKO 1 *p* < 0.0001, hetKO 1 *p* = 0.0061, hetKO 2: *p* = 0.0001. * *p* < 0.05, ** *p* < 0.01, *** *p* < 0.001.

To investigate the role of KCNQ1 in human neuronal cells, we used an iPSC line from a healthy human donor^31^, and generated *KCNQ1*-KO lines with the CRISPR/Cas9 system by deleting exon 4 and 5 with two different guide RNA (gRNA) combinations (Fig. 1a). We obtained one homozygous and three heterozygous *KCNQ1*-KO lines, as well as two unedited wildtype (WT) isogenic control lines (Fig. 1b, Supplementary Fig. 1).

Deletion of exons 4 and 5 introduced a frameshift and premature stop codon. qPCR and Western blot confirmed markedly reduced KCNQ1 expression in the homozygous KO and in two heterozygous lines (hetKO 1 and hetKO 2), whereas hetKO 3 showed no reduction (Fig. 1c, d, Supplementary Fig. 2).

As *KCNQ1* is an imprinted gene for which only the maternal allele is actively transcribed in most tissues, we investigated allele-specific editing. Imprinting of *KCNQ1* is generally not affected by the reprogramming of somatic cells into iPSCs^32^. SNP analysis showed that hetKO 1 and hetKO 2 carried the deletion on the active allele, whereas hetKO 3 was edited on the inactive allele (Supplementary Fig. 3), explaining the preserved *KCNQ1* expression in this line. All iPSC lines displayed normal karyotypes, no detectable off-target editing, and retained pluripotency (Supplementary Fig. 4)

For the investigation of KCNQ1 function in neuronal cells, iPSCs were differentiated into NSCs and cortical neurons^33^, which expressed appropriate lineage markers and exhibited expected neuronal morphology and electrophysiological properties (Supplementary Fig. 5, 6). *KCNQ1* expression remained reduced in homozygous KO cells and, to a lesser extent, in heterozygous KO cells (Fig. 1e, f). HetKO 3 did not show reduced *KCNQ1* expression in iPSCs, NSCs, or neurons (Fig. 1c-f), as it carries the deletion on the inactive allele. KCNQ1 transcript levels were within a low expression range across the human iPSC-derived system, with only modest variation between cell types, including slightly higher levels in iPSCs compared to NSCs and neurons (Supplementary Fig. 1c). Although *KCNQ1* transcripts were reproducibly detected in NSCs and neurons, endogenous KCNQ1 protein expression in the differentiated neural cells could not be conclusively demonstrated. qPCR analysis of the prefrontal cortex of *Mus musculus* (mouse) across developmental stages (E15, E17, P1, P12, and adult) indicated a similar expression range, without evident developmental changes, aside from a minor decrease in adulthood (Supplementary Fig. 1d). Consistent with these findings, Western blot analysis of adult mouse prefrontal cortex samples confirmed the presence of KCNQ1 protein, providing independent evidence for cortical KCNQ1 expression in vivo (Supplementary Fig. 7).

Allele-specific RT-PCR revealed monoallelic *KCNQ1* expression in iPSCs but biallelic expression in NSCs and neurons, indicating partial loss of imprinting during neural differentiation (Supplementary Fig. 8). KCNQ1OT1 expression remained unchanged, excluding altered expression of this imprinting regulator as the underlying cause (Supplementary Fig. 9). Although *KCNQ1* expression was not reduced in hetKO 3, this line was included in subsequent analyses because the KO allele became transcriptionally active following loss of imprinting during neural differentiation.

Having established and validated the cellular models, we next investigated the role of KCNQ1 in NSCs.

### *KCNQ1* loss and inhibition modestly reduced neurite outgrowth during early neuronal differentiation

**Fig. 2:**
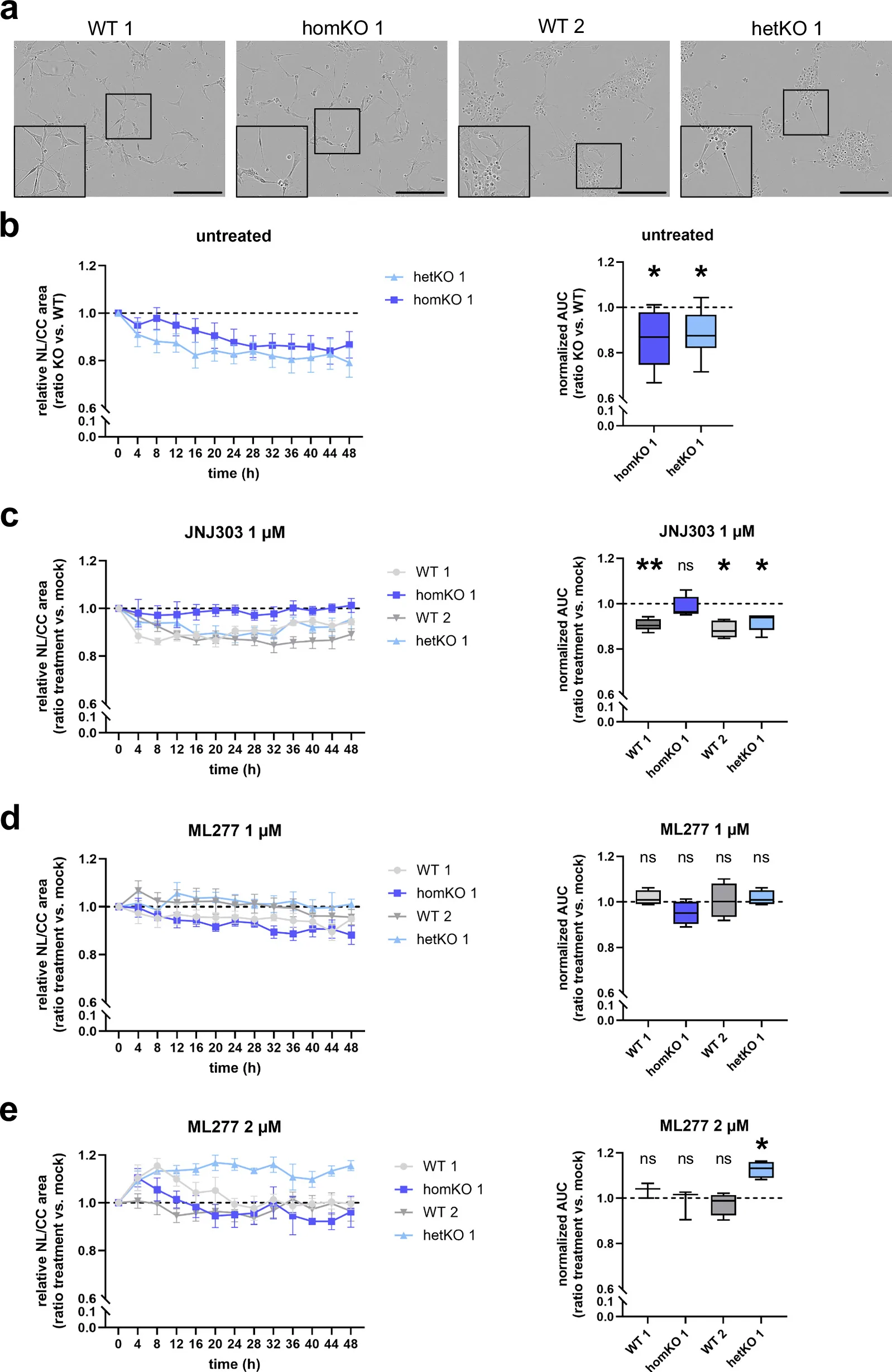
Neurite outgrowth measurement during early neuronal differentiation. **a** Representative images of differentiating NSCs 48 h after differentiation start. Scale bar: 200 µm. **b**. Neurite outgrowth was measured within the first 48 h of neuronal differentiation using the Incucyte system. Relative neurite length per cell body cluster area (NL/CC) was calculated for each KO line and normalized to its respective WT control (set to 1; homKO 1 vs WT 1, hetKO 1 vs WT 2). Data are presented as mean ± SEM (n = 9 independent experiments). The area under the curve (AUC) was calculated from normalized time-course data and analyzed using a one-sample t-test with Bonferroni correction. Adjusted *p*-values: homKO 1, *p* = 0.0176; hetKO 1, *p* = 0.0246. The dotted line indicates the normalized WT controls. **c-e** Effects of pharmacological modulation of KCNQ1 activity on neurite outgrowth during neuronal differentiation. NSCs were treated at differentiation onset with the KCNQ1 antagonist JNJ303 1 µM (IC50 = 64 nM) (**c**), or the KCNQ1 agonist ML277 (EC50 = 270 nM) at 1 µM (**d**) or 2 µM (**e**). Neurite outgrowth was monitored for 48 h and normalized to the respective mock-treated controls (DMSO, mock = 1). Data are shown as mean ± SEM. AUC values derived from normalized time-course data were analyzed using a one-sample t-test with Bonferroni correction. **c** JNJ303 treatment (n = 5 independent experiments). Adjusted *p*-values: WT 1, *p* = 0.0056; homKO 1, *p* > 0.999; WT 2, *p* = 0.0100; hetKO 1, *p* = 0.0484. **d** ML277 treatment 1 µM (n = 4 independent experiments). Adjusted *p*-values: WT 1, *p* > 0.999, homKO 1, *p* = 0.6012; WT 2, *p* > 0.999; hetKO 1, *p* > 0.999. **e** ML277 treatment, 2 µM. Relative NL/CC area normalized to the respective mock control over 48 h (mock = 1, WT 1 and homKO 1 n = 3, WT 2 and hetKO 1 n = 4 independent experiments). Adjusted *p*-values: WT 1, *p* = 0.8268, homKO 1, *p* > 0.999; WT 2, *p* > 0.999; hetKO 1, *p* = 0.0248. AUC data are shown as box-and-whisker plots; center line, median; box limits, 25th and 75th percentiles; whiskers, minimum to maximum values. * *p* < 0.05, ** *p* < 0.01.

To investigate the effects of *KCNQ1* disruption on early neuronal differentiation, we analyzed neurite outgrowth during the initial 48 h of NSC differentiation, when immature neurites emerge and develop into axons and dendrites. Live-cell imaging revealed a time-dependent increase in neurite outgrowth in all cell lines. While linear mixed-effects modelling did not detect genotype-dependent growth dynamics (Supplementary Fig. 10a), normalization within individual experiments and area under the curve (AUC) analysis uncovered a consistent reduction in neurite outgrowth in both homKO 1 and hetKO 1 compared with their respective WT controls (Fig. 2a, b). An independent differentiation protocol^34^ confirmed this phenotype (Suppl. Fig. 10b-d), supporting a role for KCNQ1 in early neuronal development. The stronger effect in hetKO 1 likely reflects differences in baseline growth between control lines.

To determine whether pharmacological inhibition phenocopies genetic *KCNQ1* loss, NSCs were treated with the selective Kv7.1 inhibitor JNJ303. Similar to the genetic KO, JNJ303 significantly reduced neurite outgrowth in both WT lines and in hetKO 1, whereas homKO 1 cells, lacking KCNQ1, were unresponsive (Fig. 2c; Supplementary Fig. 11).

Conversely, treatment with the selective KCNQ1 activator ML277 had no effect at 1 µM, whereas 2 µM partially rescued neurite outgrowth in hetKO 1 but not homKO 1 cells (Fig. 2d, e; Supplementary Fig. 12). The absence of an effect in homozygous KO cells supports the specificity of ML277 for KCNQ1 and is consistent with residual KCNQ1 activity in the heterozygous NSCs.

Although neurite outgrowth varied substantially between experiments and no significant genotype effect was detected in the raw trajectories, the normalized AUC analysis indicated a modest and reproducible reduction in early neurite outgrowth following *KCNQ1* disruption under our experimental conditions.

Neither genetic deletion nor pharmacological modulation of KCNQ1 affected NSC proliferation or apoptosis (Supplementary Fig. 13), indicating that the observed neurite phenotype is not secondary to altered cell viability.

### *KCNQ1* disruption affects multiple signalling pathways in NSCs

**Fig. 3:**
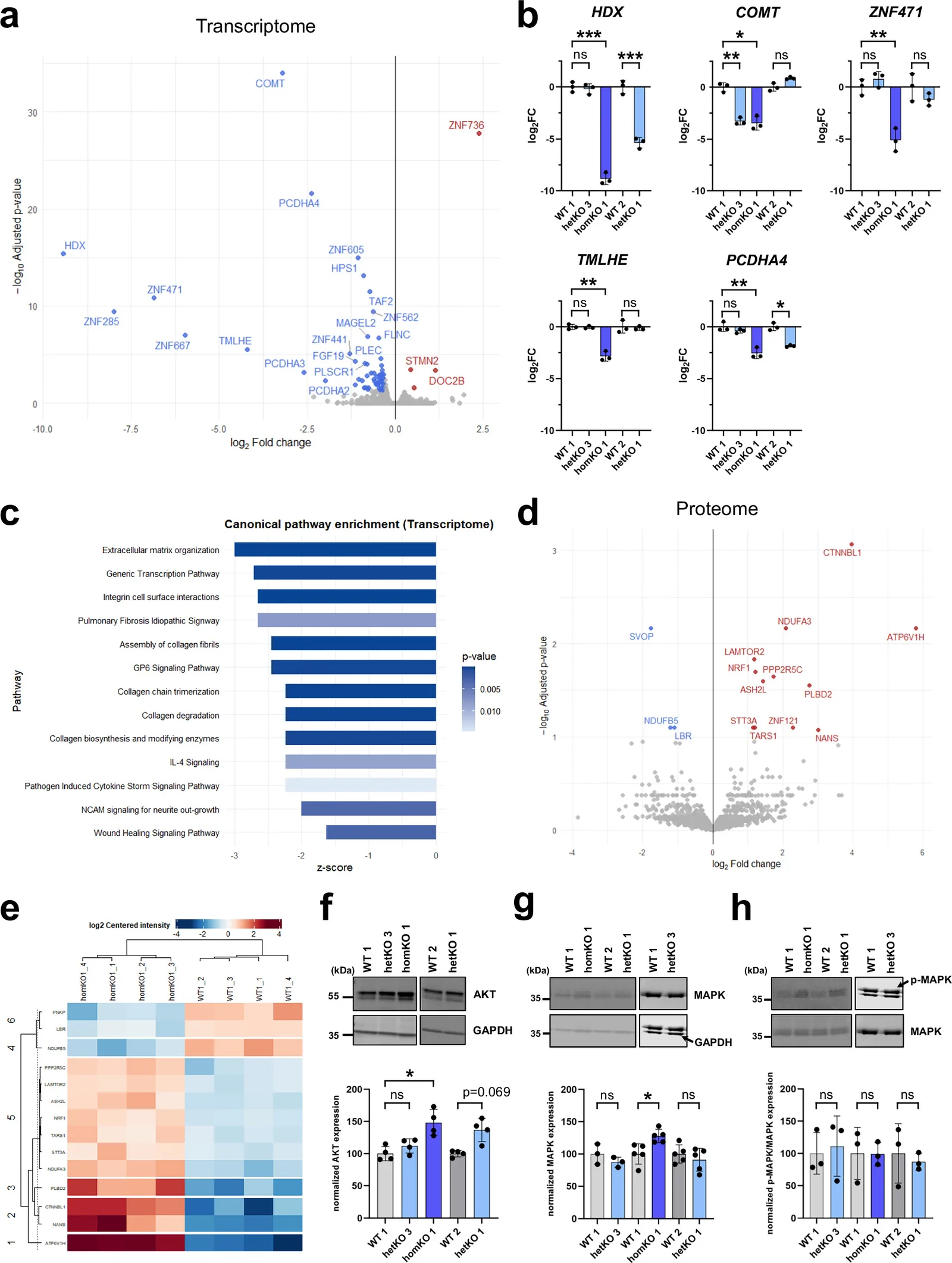
Transcriptome and proteome analysis of homozygous *KCNQ1*-KO and WT NSCs. **a** Volcano plot of transcriptome analysis from NSCs homKO 1 compared to WT 1. Genes with an adjusted *p*-value FDR < 0.05 and a log2fold change > 1 are shown. **b** Validation of transcriptome analysis by analysing the expression of the most significantly dysregulated genes in homKO 1 and additionally hetKO 1 and hetKO 3 compared to their respective WT control. Welch´s one-way ANOVA with Dunnett´s T3 post hoc test, mean ± SD, (n = 3 biological replicates). *HDX*: *p* < 0.0001, F = 117.5, post hoc test: hetKO 3: *p* = 0.9497, homKO 1: *p* < 0.0001, hetKO 1: *p* = 0.0009; *COMT*: *p* < 0.0002, F = 85.52, post hoc test: hetKO 3: *p* = 0.0014, homKO 1: *p* = 0.0114, hetKO 1: *p* = 0.1294; *ZNF471*: *p* = 0.0104, F = 11.45, post hoc test: hetKO 3: *p* = 0.5527, homKO 1: *p* = 0.0072, hetKO 1: *p* = 0.4973; *TMLHE*: *p* = 0.0042, F = 18.31, post hoc test: hetKO 3: *p* = 0.9854, homKO 1: *p* = 0.0061, hetKO 1: *p* = 0.9717; *PCDHA4*: *p* = 0.0009, F = 41.63, post hoc test: hetKO 3: *p* = 0.4958, homKO 1: *p* = 0.0078, hetKO 1: *p* = 0.0276; **c** Bar graph with significant canonical pathways from transcriptomic data. All pathways with enrichment FDR *p* < 0.05 and with z-scores assigned are shown and sorted by z-score magnitude. All these pathways have negative z-scores, indicating that, based on the profile of differentially expressed genes (at FDR *p* < 0.05), they are predicted to be inhibited. **d** Volcano plot of proteome analysis from NSCs homKO 1 compared to WT 1. Gene names are shown for proteins (for better comparison) with an adjusted *p*-value FDR < 0.10 and a log2fold change > 1.3. **e** Heatmap showing significant DEPs (FDR < 0.10) for individual biological replicates. **f-h** Western blot analysis of AKT (f), MAPK (g), and p-MAPK (h) (top: representative membrane image, bottom: quantification) mean ± SD. (f, n = 4 biological replicates; g, n = 5 biological replicates, except for WT 1 and hetKO 3 comparison, where n = 3 biological replicates; h, n = 3 biological replicates). **f** Welch´s one way ANOVA with Dunnett´s T3 post hoc test for AKT: *p* = 0.0131, F = 7.160, post hoc test: hetKO 3: *p* = 0.4104, homKO 1: *p* = 0.0230, hetKO 1: *p* = 0.0690. **g** Welch´s two-tailed t-test with Bonferroni correction, adjusted *p*-values: homKO 1: *p* = 0.0405, hetKO 1: *p* > 0.999; hetKO 3: *p* = 0.9006; **h** Welch´s two-tailed t-test with Bonferroni correction, adjusted p-values: *p* > 0.999 for all comparisons. ns – not significant. * *p* < 0.05, ** *p* < 0.01, *** *p* < 0.001.

To elucidate molecular alterations caused by *KCNQ1* loss, we performed transcriptomic and proteomic analysis of homozygous *KCNQ1*-KO and WT NSCs (passages 6 and 7 from two independent NSC inductions). Principal component analysis (PCA) revealed separation between homKO 1 and WT 1 NSCs for the transcriptomic and proteomic data (Supplementary Fig. 14). RNA sequencing identified 72 differentially expressed protein coding genes (DEGs; FDR < 0.05) the majority of which were downregulated in *KCNQ1*-KO NSCs (Fig. 3a; Suppl. Table 1 Tab1). *KCNQ1* and other members of the *KCNQ* gene family (*KCNQ2-5*), as well as genes coding for KCNQ1 interaction partners, such as *KCNE1* and *KCNE2*, were not significantly differentially expressed. They had a low read coverage and were excluded from downstream analysis.

Notably, several protocadherin (*PCDHA2-4*) and zinc finger (*ZNF285, ZNF471, ZNF667*) genes were among the most strongly downregulated, suggesting impaired cell adhesion and transcriptional regulation (labelled in yellow in Suppl. Table 1, Tab 1). qPCR validated the downregulation of selected DEGs (*HDX, PCDHA4, ZNF471, TMLHE* and *COMT*) and confirmed similar expression changes in heterozygous KO NSCs, whereas hetKO 3 largely resembled WT cells (Fig. 3b).

Pathway analysis identified inhibition of extracellular matrix organization, NCAM signalling and transcriptional pathways, together with perturbation of insulin signalling involving the RAS-MAPK and PI3K-AKT cascades (Fig. 3c; Supplementary Fig. 14, Supplementary Table 1 Tab2). Alterations in PKA and PKC signalling were also detected (Supplementary Fig. 14).

Proteomic analysis identified 15 differentially expressed proteins (FDR < 0.10; Fig. 3d, e; Suppl. Table 2 Tab 1). Several were linked to mitochondrial function, including the respiratory chain components NDUFA3 and NDUFB5 and the mitochondrial regulator NRF1, while pathway enrichment additionally implicated protein translation, metabolism and mitochondrial function (Supplementary Fig. 15, 16, Supplementary Table 2, Tabs 2, 3). These findings were consistent with the transcriptomic data and further supported dysregulation of insulin signalling and its downstream RAS-MAPK and PI3K-AKT pathways.

Together, the multi-omics analyses indicate that *KCNQ1* loss disrupts cell adhesion, gene expression, insulin signalling and mitochondrial function in NSCs, providing the basis for the subsequent functional analyses.

### Loss of *KCNQ1* affects expression of AKT and MAPK in NSCs

As transcriptomic and proteomic analyses implicated insulin signalling in *KCNQ1*-KO NSCs (Table 1), we examined the expression of the key downstream regulators AKT and MAPK by Western blotting. AKT protein levels were increased in homKO 1 and hetKO 1 compared to their respective controls, although the increase in hetKO 1 did not remain significant after multiple-testing correction (Fig. 3f, Supplementary Fig. 17). Phosphorylated AKT (Ser473) was not detected. MAPK expression was also increased in homKO 1 NSCs, whereas phosphorylation levels (Thr202/Tyr204) were unchanged (Fig. 3g, h; Supplementary Fig. 18a, 18b), indicating increased levels of active MAPK. No differences were observed in hetKO 3 compared with WT 1. Together, these findings indicate increased AKT and MAPK expression in homozygous *KCNQ1*-KO NSCs.

**Table 1:** Overview of enriched canonical pathways and biological processes identified in homozygous *KCNQ1*-KO NSCs and neurons by transcriptomic and proteomic analyses

Cell type	Transcriptome canonical pathways, network enrichment (IPA)	Proteome canonical pathways (IPA)^*^ & GSEA^#^
NSCs	Integrin cell surface interaction Generic transcription pathway NCAM signalling for neurite outgrowth Protein kinase A & C signalling Insulin signalling (RAS/ERK, PI3K)	Cell adhesion ^#^ Gene expression regulation ^*, #^ Protein kinase C signalling ^#^ Insulin-like growth factor receptor signalling pathway ^#^ Protein translation ^#^ Mitochondria function ^*, #^ Metabolism ^*^
neurons	Generic transcription pathway Insulin signalling - ERK - AKT	Regulation of transcription ^#^
		Regulation of insulin secretion ^#^ Insulin responsive compartment ^#^
		Vesicle trafficking/recycling (GARP complex)^#^ Protein translation ^*, #^ Mitochondria function ^#^ Layer formation in the cerebral cortex ^#^ Cellular growth, proliferation and development ^*^ Cell cycle ^#^
		Metabolism of RNA and proteins ^*^

To identify molecular impairments, enrichment analyses were performed using IPA for transcriptomic data, and both IPA (*) and GSEA (#) for proteomic data. Signalling pathways identified on transcript level were specifically searched for among the significant GO terms (GSEA) and canonical pathways (IPA) on protein level and listed in this table. A summary of the most significant biological processes, cellular compartments, and molecular functions from GSEA (20 most significant), and from the IPA cellular mechanisms and canonical pathways is shown. The full data sets can be found in Supplementary Table 2 (Tabs 2, 3, 5, 6).

### Loss of *KCNQ1* impairs mitochondrial biogenesis in NSCs

**Fig. 4:**
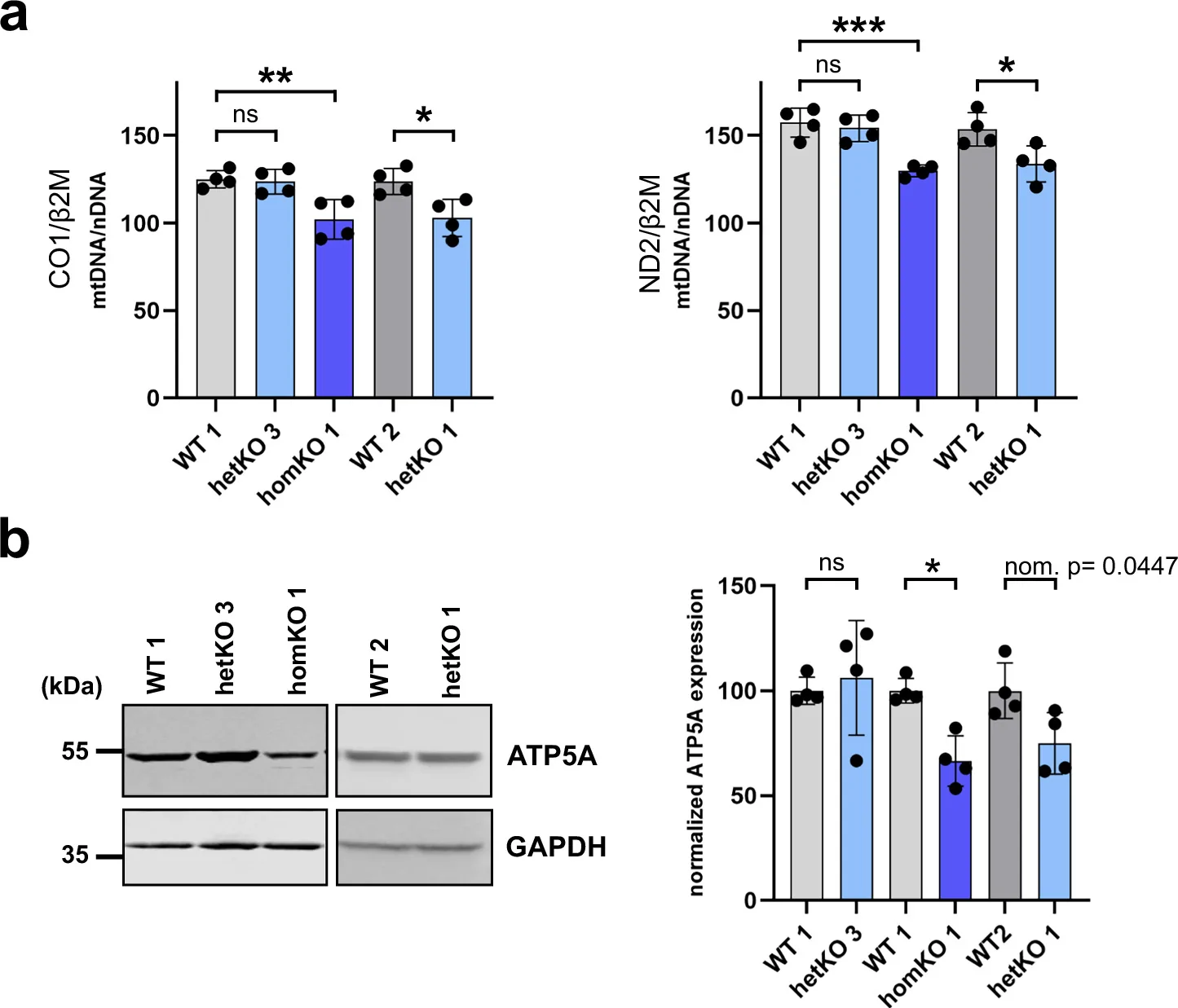
Analysis of mitochondrial copy number and ATP5A expression. **a** The amount of mitochondrial DNA was determined by quantification of two mitochondrial genes (*CO1, ND2*) relative to the amount of DNA of the nuclear gene *β2M*. One-way ANOVA with Bonferroni-adjusted post hoc test, mean ± SD, (n = 4 biological replicates). *CO1/β2M*: *p* = 0.0016, F = 7.5, post hoc test: hetKO 3: *p* > 0.999, homKO 1: *p* = 0.0059, hetKO 1: *p* = 0.0118; *ND2/β2M*: *p* = 0.0004, F = 9.940, post hoc test: hetKO 3: *p* > 0.999 homKO 1: *p* = 0.0008, hetKO 1: *p* = 0.0113. **b** ATP5A expression was measured by Western blot (left: representative membrane image, right: quantification). HomKO 1 and hetKO 3 were compared to WT 1 but analyzed on separate membranes using independent WT 1 biological replicates; therefore, WT 1 values are membrane-specific and not directly comparable across panels. Statistical comparisons were restricted to within-membrane pairwise analyses and performed using a two-tailed Welch´s t-test. (n = 4 biological replicates), nominal *p*-values: hetKO 3: *p* = 0.685, homKO 1: *p* = 0.006, hetKO 1: *p* = 0.0447; Bonferroni corrected p-values: hetKO 3: *p* > 0.999, homKO 1: *p* = 0.018, hetKO 1: *p* = 0.1341, values were normalized to mean of the respective WT control which was set to 100. *ND2*: *NADH dehydrogenase 2*; *CO1*: *cytochrome C oxidase 1*; *β2M*: *beta-2 microglobulin*; ATP5A: ATP synthase F1 subunit alpha. nom. – nominal p-value. * *p* < 0.05, ** *p* < 0.01, *** *p* < 0.001.

To follow up on the mitochondrial alterations indicated by the proteomic analysis, we analyzed mitochondrial DNA (mtDNA) copy number and deletion. While no evidence of mtDNA deletions was detected (Supplementary Fig. 19b), mtDNA copy number was significantly reduced in homKO 1 and hetKO 1 NSCs compared with their respective controls (Fig. 4a), suggesting impaired mitochondrial biogenesis. Consistent with this, pathway enrichment analyses implicated mitochondrial biogenesis, respiratory electron transport, mitochondrial fusion, and cytochrome c release (Supplementary Fig. 16; Supplmentary Table 2, Tabs 2, 3). We next quantified the mitochondrial ATP synthase subunit ATP5α by Western blotting and observed reduced expression in homKO 1 NSCs (Fig. 4b, Supplementary Fig. 20). ATP5α levels were also decreased in hetKO 1, reaching nominal significance only, whereas hetKO 3 resembled WT 1.

To determine whether these molecular alterations were accompanied by changes in mitochondrial architecture, we quantified mitochondrial area per nucleus, foreground length per nucleus, aspect ratio, and circularity in MitoTracker-stained NSCs. Despite the reductions in mtDNA copy number and ATP5α expression, linear mixed-effects analysis revealed no significant effect of genotype on any mitochondrial morphology parameters (Supplementary Fig. 21). Although variability between biological replicates was relatively high, no consistent genotype-dependent differences in mitochondrial content or network morphology were observed. Together, these findings suggest that *KCNQ1* loss is associated with impaired mitochondrial biogenesis and reduced ATP synthase expression without detectable alterations in mitochondrial abundance or network organization under the experimental conditions examined.

### *KCNQ1* loss is accompanied by impaired neuronal maturation and reduced synaptic activity

**Fig. 5:**
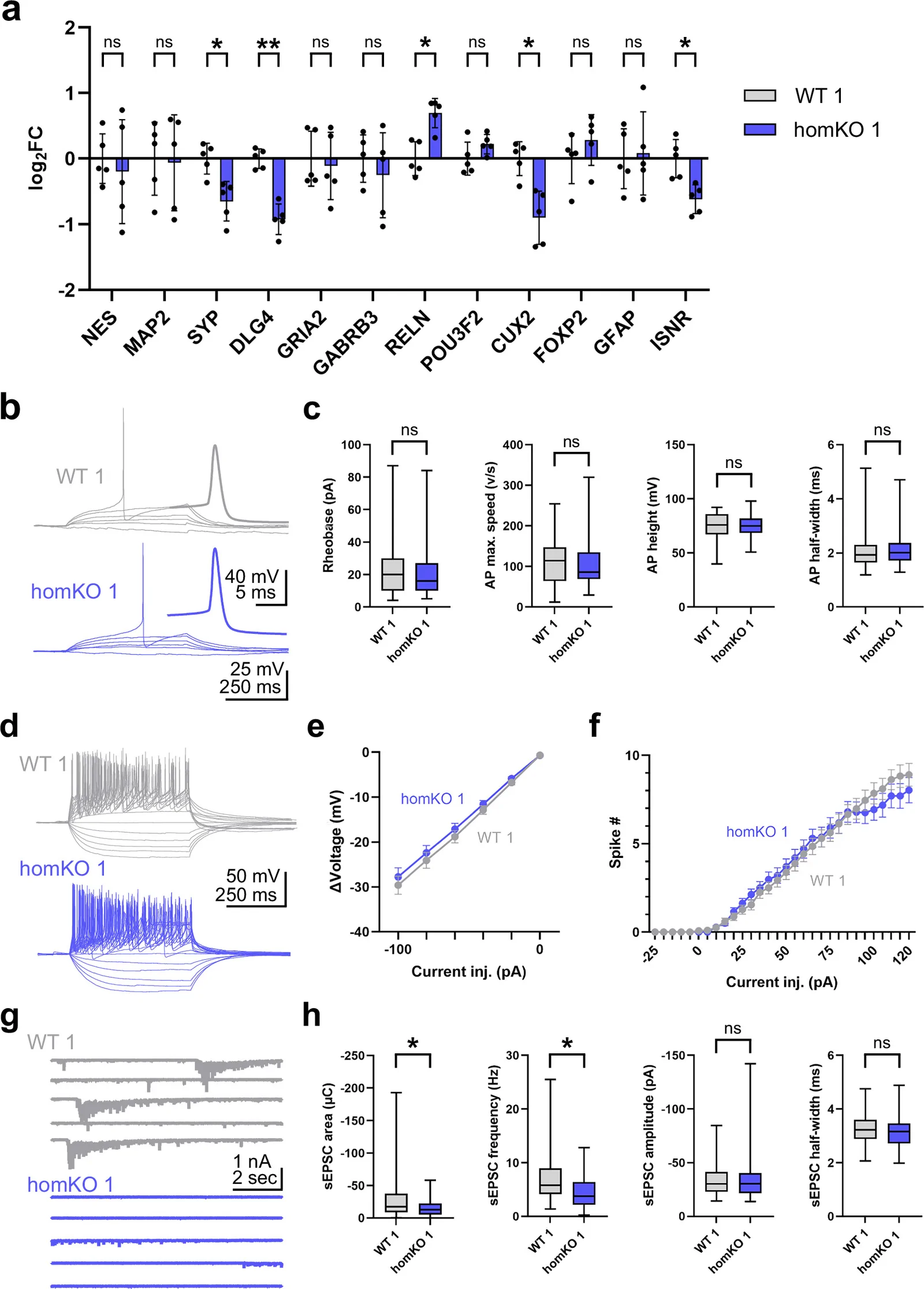
Impact of *KCNQ1* deletion on intrinsic properties and synaptic activity in neurons. **a** Expression analysis of 12 marker genes in *KCNQ1*‑KO neurons and control lines (WT 1, WT 2, hetKO 1, hetKO 3, homKO 1,). Significant differences were detected only in homKO 1 compared with WT 1 for *SYP*, *DLG4*, *RELN*, *CUX2*, and *ISNR*. The full marker set for homKO 1 is shown, as only homKO 1 neurons showed statistically significant results; the complete dataset is provided in Supplementary Table 1 (Tab4). Data are shown as mean ± SD (n = 5 biological replicates). Statistical analysis was performed using multiple unpaired two‑tailed Welch’s t‑tests with Benjamini–Hochberg adjustment for multiple comparisons. Adjusted *p*-values: *SYP*: *p* = 0.04311; *DLG4*: *p* = 0.006; *RELN*: *p* = 0.03526; *CUX2*: *p* = 0.04311; *ISNR*: *p* = 0.04311. **b- c** Basic properties of neurons. **b** Representative recordings of a control (WT 1) and a mutant (homKO 1) neuron. Cells were kept at near -70mV holding potentials and progressively depolarized using positive current injections (from 0 pA, +5 pA steps for 500 ms). **c** Summary plots of rheobase, as well as action potential (AP) max speed, height, and half-width. Number of experiments: Rheobase WT 1 and homKO 1: 55 cells/3 batches, AP properties (all graphs) WT 1: 43 cells/3 batches, homKO 1: 46 cells/3 batches. Unpaired two-tailed Student’s *t*-test, mean ± SEM. Rheobase: *p* = 0.4239; AP max. speed: *p* = 0.7198; AP height: *p* = 0.9943; AP half-width: *p* = 0.8290. **d, e** Neuronal excitability. **d** Representative recordings of a control (WT 1) and a mutant (homKO 1) neuron. Cells were kept at near -70mV holding potentials and subjected to a series of current injection steps (from -100 to +480 pA, 20 pA steps for 500 ms). **e** Summary plot of voltage responses upon injections of hyperpolarizing current injections (from -100 pA to 0 pA). Number of experiments for WT 1 and homKO 1: 45 cells/3 batches for each. Two-way ANOVA (injected current x cell line) with Bonferroni-adjusted post hoc test, mean ± SEM, all *p* > 0.05. **f** Summary plot of spiking activity upon injections of depolarizing current injections (from 0 pA to 480 pA). Number of experiments: WT 1 45 cells/3 batches; homKO 1: 44 cells/3 batches. Two-way ANOVA (injected current x cell line) with Bonferroni-adjusted post hoc test, mean ± SEM, all *p* > 0.05. **g, h** Spontaneous excitatory postsynaptic currents (sEPSCs). **g** Representative recordings of a control and a mutant neuron at -70 mV holding potentials. **h** Summary graphs of sEPSC integrated charge, sEPSC frequency, amplitude, and half-width. Number of experiments: WT 1 and homKO 1 45 cells/3 batches for each. Unpaired two-tailed Student’s *t*-test, mean ± SEM. sEPSC charge: *p* = 0.0183; sEPSC frequency: 0.0123; sEPSC amplitude: *p* = 0.5617; sEPSC half-width: *p* = 0.2023. *NES*: Nestin; *MAP2*: microtubule associated protein2; *SYP*: synaptophysin; *DLG4*: discs large MAGUK scaffold protein 4; *GRIA4*: glutamate ionotropic receptor AMPA type subunit 4; *GABRB3*: gamma-aminobutyric acid type A receptor subunit beta 3; *RELN*: reelin; *POU3F2*: POU class 3 homeobox 2; *CUX2*: cut like homeobox 2; *FOXP2*: forkhead box P2; *GFAP*: glia fibrillary acidic protein; *ISNR*: insulin receptor. Data are shown as box-and-whisker plots; center line, median; box limits, 25th and 75th percentiles; whiskers, minimum to maximum values.* *p* < 0.05, ** *p* < 0.01.

To investigate the role of *KCNQ1* in cortical neurons, we analysed genes that are typically expressed in neurons and their precursors (Fig. 5a, Supplementary Table 1 Tab4) in *KCNQ1*‑KO neurons and control lines (WT 1, WT 2, hetKO 1, hetKO 3, homKO 1). Homozygous *KCNQ1*-KO neurons showed reduced expression of the synaptic markers *SYP* and *DLG4*, the upper-layer cortical marker *CUX2*, and the insulin receptor gene *INSR*, while *RELN*, an early neuronal marker, was increased compared with WT 1. No significant changes were detected in the heterozygous KO lines, except for a nominal reduction in *INSR* expression in hetKO 1 (Suppl. Table 1, Tab 4). The differentiated cultures contained a small proportion of GFAP-positive immature astrocyte-like cells, with a comparable low *GFAP* expression (Fig. 5a, Supplementary Table 1 Tab 4, Supplementary Fig. 6). Together, these findings indicate delayed neuronal maturation and impaired insulin signalling following homozygous *KCNQ1* loss.

Patch-clamp recordings revealed no differences in action potential properties or intrinsic neuronal excitability between homozygous *KCNQ1*-KO and WT neurons (Fig. 5b-f). In contrast, excitatory synaptic transmission was reduced, as demonstrated by decreased spontaneous excitatory postsynaptic current (EPSC) frequency and overall charge transfer (Fig. 5g, h). These alterations were absent in heterozygous *KCNQ1*-KO neurons (Supplementary Fig. 22a), consistent with residual *KCNQ1* expression due to partial loss of imprinting (Supplementary Fig. 8). Together, these findings indicate that homozygous *KCNQ1* loss impairs excitatory synaptic function, likely reflecting delayed neuronal maturation.

### *KCNQ1* disruption affects multiple signalling pathways in neurons

Transcriptomic and proteomic analyses were performed on iPSC-derived cortical neurons from WT and *KCNQ1*-KO lines (WT 1, WT 2, homKO 1, hetKO 1, hetKO 2, and hetKO 3) as described for NSCs. RNA sequencing detected 15,231 protein-coding genes and the PCA plot did not reveal a clear separation between the WT and the KO groups, suggesting minimal differences in the underlying data, and a low impact of the *KCNQ1*-KO on transcription in neurons (Supplementary Fig. 22b). This is in line with the fact that potassium channels mostly influence transcription indirectly after their activation, whereas our analysis was carried out under basic cellular conditions. However, differential expression analysis identified significant DEGs across the *KCNQ1*-KO lines (FDR < 0.05, Supplementary Fig. 22c, 23a, b Supplementary Table 1, Tabs 5–6)). Shared DEGs were enriched for genes involved in mitochondrial function (*TXNRD2*), transcriptional regulation (*ZFP3, ZNF471, ZNF585A/B, HOXB9*), cell adhesion (*PCDHA4, PCDHGC3*), neurodevelopment (*NGFR, NHLH2*), and insulin regulation (*ISL1*) (Supplementary Fig. 23c, Supplementary Table 1 (Tab 7)). Validation by nCounter analysis confirmed differential expression for 14 of 16 selected genes (Supplementary Fig. 23d, Supplementary Table 1 (Tab 8)), demonstrating high reproducibility of the RNA sequencing data. Pathway analysis identified reduced transcriptional activity and implicated insulin signalling, including the RAS-MAPK and PI3K-AKT pathways (Table 1, Supplementary Table 1 (Tabs 9-12), Supplementary Fig. 24, 25).

**Fig. 6:**
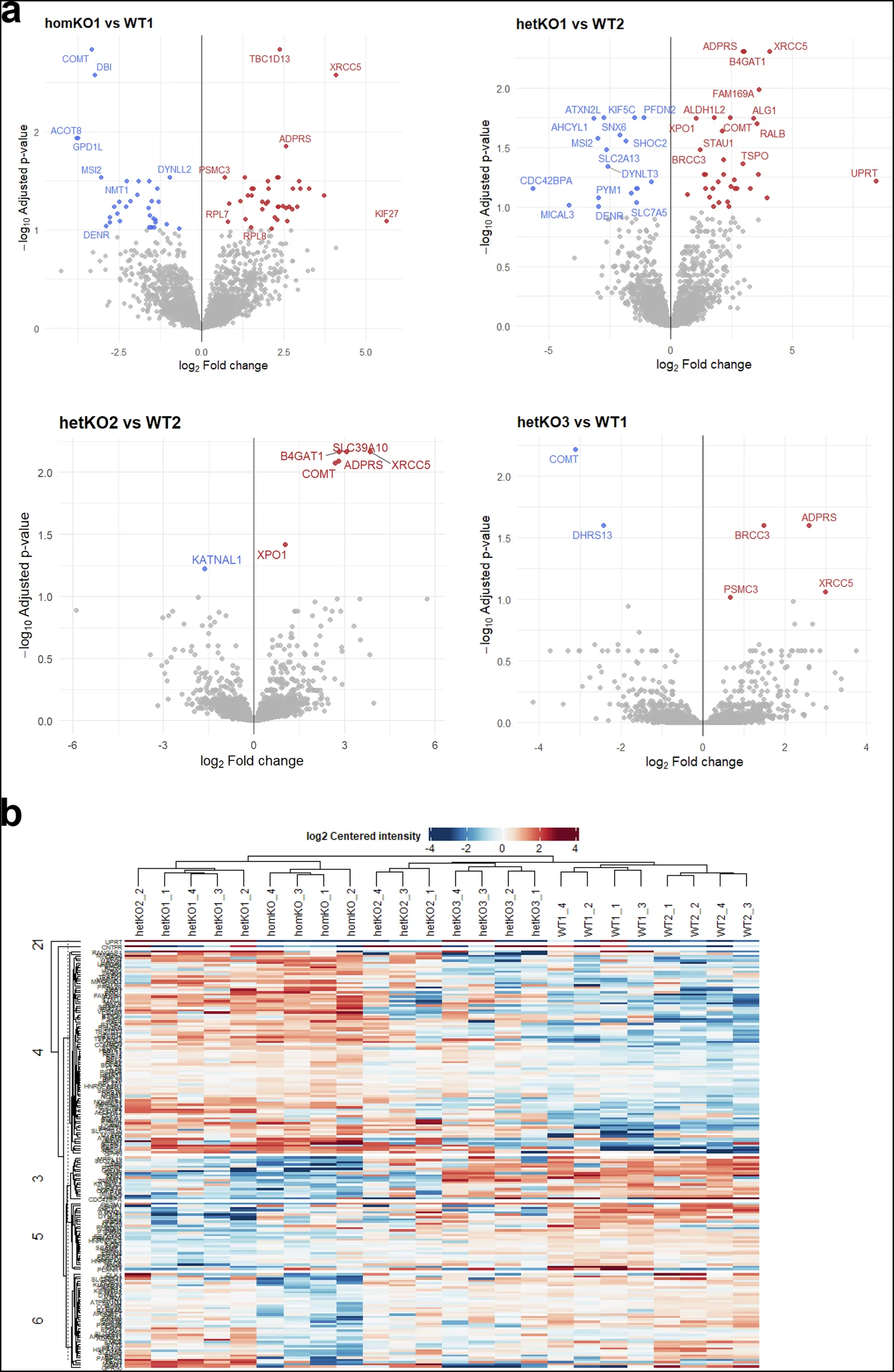
Proteomic data of *KCNQ1*-KO neurons. **a** Volcano plots showing DEPs in neurons (FDR < 0.1, log2fold change > 1.3). Significantly upregulated proteins are shown in red, downregulated proteins in blue. **b** The heatmap shows the log2 centered intensity of all DEPs in homKO 1. The dendrogram indicates separation between WT and KO cell lines, where homKO 1 and hetKO 1 as well as hetKO 2 and hetKO 3 are clustering closer together.

Proteomic analysis identified 2,990 proteins and revealed clearer genotype-dependent separation than the transcriptomic data (Supplementary Fig. 26). Using an FDR cutoff of < 0.10, we identified significantly altered neuronal proteins (Fig. 6a, b; Suppl. Table 2 Tab 4). Differentially expressed proteins in homozygous *KCNQ1*-KO neurons implicated mitochondrial metabolism (ACOT8, GPD1L, DBI, DHRS13 ATAD3B, MAVS, TSPO), RAS-MAPK and PI3K-AKT signalling (KRAS, STRN3, MSI2, ROBO1, NMT1, TBC1D13), and mTOR-dependent protein translation (Fig. 6a, b; Suppl. Table 2, Tab5, Tab6). Several proteins, including TSPO, MSI2 and STAU1, were consistently altered in both homozygous and heterozygous *KCNQ1*-KO neurons. TSPO is a mitochondrial protein involved in signalling and metabolism. STAU1, is an RNA-binding protein important for mRNA transport and localization. MSI2 is a post-transcriptional regulator involved in neuronal differentiation. Pathway enrichment further indicated impairments in neuronal growth, development and protein translation (Table 1; Supplementary Fig. 27), supporting the immature neuronal phenotype observed in *KCNQ1*-KO cultures.

Because the omics analyses implicated insulin signalling and mitochondrial function, we performed targeted follow-up experiments. In contrast to NSCs, however, neither mitochondrial DNA copy number nor ATP5α expression was altered in *KCNQ1*-KO neurons (Supplementary Fig. 19, 20), suggesting that these mitochondrial defects are specific to the NSC stage.

### *KCNQ1* disruption affects similar pathways in NSCs and neurons

Several transcriptomic alterations identified in homozygous *KCNQ1*-KO NSCs persisted in differentiated neuronal cultures, particularly those affecting gene transcription and insulin signalling, including the AKT and MAPK pathways. At the protein level, GSEA and IPA identified shared alterations in NSCs and neurons involving protein translation, gene transcription, mitochondrial function, and cellular metabolism (Table 1). Comparative proteomic analysis further highlighted the ATPase complex and mitochondrial protein-containing complexes as among the most significantly affected cellular compartments in both cell types. Enrichment of mitochondrial transport, the inner mitochondrial membrane, cytochrome c release, and ATP-related processes, including ATP hydrolysis and proton transmembrane transport, indicates impaired cellular energy metabolism following *KCNQ1* loss (Supplementary Fig. 28). The consistency of these findings across NSCs and neuronal cultures supports a common role for KCNQ1 in regulating mitochondrial and metabolic function.

## Discussion

To investigate the role of KCNQ1 in human neuronal development, we generated *KCNQ1*-KO and isogenic control iPSC lines, which were subsequently differentiated into NSCs and cortical neurons. We observed that KCNQ1 already plays a role in early neuronal differentiation, influencing neurite outgrowth, mitochondrial function, and cellular energy metabolism. In differentiated neuronal cultures, *KCNQ1* loss was accompanied by reduced functional activity, and a more immature gene expression profile. Combined transcriptomic and proteomic analyses revealed that key biological processes — including gene transcription, protein translation, mitochondrial function, and insulin signalling — were disrupted in both NSCs and neuronal cultures. These results indicate that molecular dysfunctions originating during early neurodevelopment persist throughout neuronal maturation.

In NSCs, both homozygous and heterozygous *KCNQ1*-KOs showed reduced neurite outgrowth during early neuronal differentiation. Moreover, pharmacological inhibition of KCNQ1 channels with the antagonist JNJ303 led to reduced neurite outgrowth in both WT and hetKO 1 NSCs, whereas no effect was observed in homKO 1 NSCs. Notably, the reduced neurite outgrowth in the hetKO 1 NSCs could be rescued by activating the Kv7.1 channel with the agonist ML277. Even though *KCNQ1* is an imprinted gene and the deletion in hetKO 1 affects the active allele, this NSC line also expressed the WT allele which explains the rescue effect. As a possible underlying mechanism for the observed diminished neurite outgrowth, transcriptome and proteome analyses pointed towards impairments in cell adhesion and a decrease in NCAM signalling, a known regulator of neurite outgrowth. Additionally, the omics data revealed that loss of *KCNQ1* affects PKA and PKC signalling pathways in NSCs. While transcriptomic data predicted an inhibition of both PKA and PKC signalling, the PKC signalling seems to be activated at the protein level (GSEA), suggesting the involvement of post-transcriptional regulation or compensatory responses. Importantly, both PKA and PKC modulate KCNQ1 activity through phosphorylation and are known to regulate key cellular processes such as adhesion, proliferation, differentiation, and metabolism^35–37^. Therefore, dysregulation of these pathways may also contribute to the observed cellular phenotype.

The loss of *KCNQ1* in heterozygous and homozygous KO NSCs results in mitochondrial dysfunction, characterized by a decrease in mitochondrial biogenesis — as evidenced by reduced mitochondrial DNA copy numbers. Moreover, the expression of a subunit of the ATP-synthase complex (ATP5α), a key component in ATP production, is diminished, suggesting decreased ATP production and a potentially compromised cellular energy metabolism. Proteomic analysis further supported these findings, revealing alterations in pathways related to mitochondrial biogenesis, respiratory electron transport, and cellular metabolism. In contrast, quantitative analysis of mitochondrial morphology demonstrated no significant changes in mitochondrial area, network length, aspect ratio, or circularity, indicating that the molecular defects are not accompanied by overt alterations in mitochondrial abundance or network architecture. This dissociation suggests that *KCNQ1* deficiency primarily impairs mitochondrial function and biogenesis rather than mitochondrial structural organization, and that mitochondrial morphology is maintained despite reduced respiratory capacity.

During the differentiation of NSCs into neural progenitor cells, and eventually neurons, cells undergo an energetic transition from the dominance of glycolysis to relying on mitochondrial oxidative phosphorylation (OxPhos) — a more efficient mode of ATP generation^38^. As the process of neuronal differentiation increases energy demands, an impaired mitochondrial energy supply very likely explains the observed reduction in neurite outgrowth in differentiating NSCs lacking *KCNQ1*. In these KO cells, the metabolic shift from glycolysis to OxPhos may be delayed. Moreover, a possible ATP deficiency could affect phosphorylation of KCNQ1 directly and indirectly through PKC signalling^39^. Taken together, these results suggest that KCNQ1 plays an important role in regulating cellular energy metabolism.

Further, loss of *KCNQ1* in NSCs and neurons alters insulin signalling. This was initially observed in the enrichment analysis of the DEGs and DEPs in the homKO 1 dataset, which indicated a dysregulation of the insulin signalling, including downstream the RAS-MAPK and PI3K-AKT cascades. Furthermore, gene expression of the insulin receptor was significantly reduced in homKO 1 neurons relative to WT controls. These neurons showed a general enrichment of proteins regulated by insulin (insulin-responsive compartment). In particular, pathways related to insulin secretion and insulin receptor signalling were enriched, with additional evidence supporting the involvement of AKT and MAPK signalling. AKT and MAPK, both regulate cytoskeleton dynamics and promote neurite outgrowth^40^. As previously reported, both pathways respond to a wide range of stimuli, including growth factors, cytokines, nutrient levels, and cellular stress, enabling them to tightly regulate cellular energy homeostasis and mitochondrial function. Remarkably, AKT and MAPK signalling promote mitochondrial biogenesis, enhance mitochondrial fusion, and support OxPhos and ATP production^41,42^. Therefore, the upregulation of AKT and MAPK observed in *KCNQ1*-deficient NSCs by Western blotting likely reflects a compensatory mechanism aimed at restoring mitochondrial biogenesis and function, which were found to be impaired.

In our differentiated neuronal cultures, *KCNQ1* loss resulted in a reduced frequency of spontaneous EPSCs, indicating impaired synaptic activity. This is in contrast to the well-established role of potassium channels in dampening neuronal excitability by stabilizing the membrane potential and facilitating action potential repolarization. In the current study, this reduction is likely due to the delayed maturation of homKO 1 compared to WT neurons. HomKO 1 neurons exhibit lower expression of synaptic genes such as *SYP* and *DLG4*, increased expression of the cortical layer I marker *RELN*, and decreased expression of the layer II–IV marker *CUX2*. Additional evidence for impairments of neuronal differentiation was obtained at protein level pointing towards impairments in the layer formation of the cerebral cortex (GSEA) and cell proliferation and growth (IPA). Insulin signalling is critically important for early neuronal development both in vivo and in vitro, as it regulates NSC proliferation, neuronal differentiation, axonal and dendritic growth, synaptogenesis, and neuronal survival^43^. Thus, the combination of decreased insulin receptor expression and disrupted cellular energy metabolism very likely contributes to the reduced maturity phenotype of homKO 1 neuronal cultures and their reduced spontenous EPSC frequency.

The presence of related phenotypes in comparatively homogeneous NSC cultures is consistent with an effect of *KCNQ1* disruption within the neural precursor population. However, these NSCs can give rise to both neurons and astrocytes, and the differentiated neuronal cultures contained a small proportion of GFAP-positive immature astrocyte-like cells. Therefore, the observed neuronal maturation and electrophysiological phenotypes cannot be considered strictly neuron-autonomous, and a limited indirect contribution from astroglial cells cannot be excluded.

Taken together, this study provides evidence that *KCNQ1* disruption affects NSC properties and neuronal differentiation in a human iPSC-derived neural model. Our findings show that KCNQ1 influences key cellular processes, including cell adhesion, transcriptional regulation, insulin signalling, and mitochondrial function. *KCNQ1* disruption also impaired neuronal maturation and synaptic activity, potentially through changes in insulin signalling and cellular energy metablism. Clinically, *KCNQ1* variants are linked to disorders of peripheral organs — such as long/short QT syndrome, atrial fibrillation, Jervell and Lange-Nielsen syndrome, Romano-Ward syndrome, and type 2 diabetes (DM2) — as well as neurological conditions including epilepsy and OCD^10–20^. Some genetic variants were even directly linked to comorbidities, such as long QT syndrome with epilepsy. Thus, elucidating the role of KCNQ1 in human neuronal cells contributes to a better understanding of these disease comorbidities. Individuals with pathogenic *KCNQ1* variants, while initially diagnosed with cardiac disease, may also have an elevated risk for DM2, epilepsy, cognitive impairment, and psychiatric disorders^21,22^.

While this study provides important insights, some limitations should be taken into account when interpreting the results. The use of human iPSC-derived neural stem cells and early cortical neurons, enables controlled mechanistic analysis of KCNQ1 function but, as an in vitro system, does not fully recapitulate the cellular diversity and circuit complexity of the developing brain. Therefore, the relevance of the observed cellular phenotypes to higher-order brain function requires validation in complementary systems, including animal models and human brain organoids. Although many results were statistically significant and reproducible — for instance, the observed reduction in neurite outgrowth during NSC differentiation — the identified effect sizes were modest. This may reflect the inherently low expression levels of KCNQ1 in NSCs and neurons. In addition, the sample size was limited, as only one homozygous *KCNQ1*-KO iPSC clone was available, and most functional analyses in NSCs were conducted using this clone alongside a single heterozygous KO line and their respective isogenic controls. The use of the heterozygous line, carrying the disruption on the active allele, was supported by prior evidence for stable imprinting at the KCNQ1 locus in human iPSCs^32^. However, the heterozygous condition did not fully recapitulate the homozygous phenotype, reflecting residual or reactivated KCNQ1 expression in NSCs and neurons. Validation in additional independent homozygous KO clones would further strengthen the conclusions. Different significance thresholds needed to be applied for transcriptomic and proteomic analyses. While transcriptomic data were evaluated at FDR < 0.05, a less stringent threshold (FDR < 0.10) was used for proteomic data due to the limited number of differentially expressed proteins. Further investigation will provide valuable insights into the extent of insulin signalling and mitochondrial dysfunction in neurons with *KCNQ1* loss and deepen our understanding of the associated metabolic and energetic consequences. A detailed biophysical characterization of KCNQ1 channel function was beyond the scope of this study. Future studies should examine potassium-ion permeability in NSCs and assess slow‑inactivating potassium currents resembling IKs or IM in human neurons. Electrophysiological characterization, ideally combined with genetic rescue and pharmacological validation, will be required to determine whether KCNQ1-dependent channel activity contributes directly to the observed phenotypes. Such analyses will help distinguish developmental consequences of *KCNQ1* disruption from direct effects on mature neuronal excitability.

Studying patient-specific *KCNQ1* variants in iPSC-derived cardiomyocytes, NSCs and neurons, holds promise for identifying common mechanisms of comorbidity and assessing individual patient risk. A comparative analysis of our findings in NSCs and neurons with mechanisms observed in cardiac cells will help to determine whether shared or distinct cellular impairments underlie the observed comorbidities.

Notably, our data shows that KCNQ1 has a strong effect on several molecular pathways in neurons and their progenitors — particularly those involving insulin signalling and mitochondrial function — which have also been implicated in the development of insulin resistance and related disease phenotypes^44^. Potassium homeostasis and insulin signalling are intricately connected influencing neuronal excitability and synaptic function^45^. Previous studies showed that dysregulation in brain K⁺ channels impairs neuronal insulin sensitivity, potentially contributing to disorders such as obesity and DM2^46^. Studies in mice even revealed that blocking of voltage-gated potassium channels ameliorated diabetic cognitive dysfunction^45^. Therefore, targeting potassium channels, including the Kv7.1 channel, may be a promising strategy for the treatment of comorbid cognitive impairments and diabetes.

## Methods

### IPSC culturing

IPSCs were cultured in mTeSR Plus medium (100-0276, StemCell Technologies), seeded on Geltrex (A1413302, Thermo Fisher) and either split with Gentle Cell Dissociation Reagent (GCDR; clump splitting; 100-0485, StemCell Technologies) or Accutase (single cell splitting; A1110501, Thermo Fisher), depending on the experiment. For single cell splitting with Accutase, Rho kinase (ROCK) inhibitor (Y27632; HY-10583, Hycultec) was used for 24 h after the split to increase cell survival. Cells were split when reaching 70-80% confluency (every 4-5 days).

### CRISPR/Cas9 editing

A previously published iPSC line (T12) derived from a healthy donor (*Homo sapiens*) was used in this study^31^. Guide RNAs (gRNAs) flanking the exons 4 and 5 of *KCNQ1* (hg38, MANE transcript NM_000218.3) were designed using the CRISPR/Cas9 target online predictor CCTop^47^. Two gRNAs were used in combination to delete both exons (two different gRNA combinations, gRNA 1 + gRNA 2, gRNA 3 + gRNA 4) (Fig. 1a). Target sequences of the used guide RNAs: gRNA 1 – GCAGGGTGTATGCTCTTCC, gRNA 2 – GTTCAGGTACCCCTGCGCCC, gRNA 3 – ATACACCCTGCTCTCGTCTG, gRNA 4 – CCCATCTGAGTGCAGCCCTC. Further details on the gRNAs are provided in the Supplementary materials and methods. To generate the KO cell lines, iPSCs were split with Accutase. TrueCut Cas9 protein v2 (A36497, Thermo Scientific) and gRNAs were introduced into the cells by nucleofection. Subsequently, per reaction 200.000 cells were seeded in a 24-well. When cells reached confluency, single cell cloning was performed by seeding 500 or 1000 cells on a 6 cm dish. Colonies were picked after 7 days and placed into a 96-well plate. After 5-7 days colonies reached 70-80% confluency and were further expanded. During this split, part of the cells was used for DNA isolation and KO-screening by PCR. One homozygous iPSC line (homKO 1) and three heterozygous KO clones (hetKO 1, hetKO 2, hetKO 3) were selected. Two unedited lines were used as isogenic WT controls (WT 1, WT 2). The KO was confirmed by PCR across the deleted region followed by Sanger sequencing to determine the exact position and size of the deletion (gRNA 1 + gRNA 2: 960 bp, gRNA 3 + gRNA 4: 1207 / 1219 bp) (Supplementary Fig. 1). Two heterozygous clones carry the deletion on the active allele (hetKO 1, hetKO 2), and one cell line on the inactive allele (hetKO 3) (Supplementary Fig. 1). Cell lines generated with the same gRNA were compared in the follow-up experiments (Fig. 1b).

### Immunocytochemistry

100.000 cells were seeded on coverslips in a 24-well format and after growing 2-3 days fixed with 4% PFA for 15 min at room temperature (RT). For immunocytochemistry, cells were permeabilized with 0,1% Triton X-100 in PBS for 6-7 min at 4°C. Cells were blocked for 1 h at RT in blocking buffer (10% NGS in PBS). Primary antibody was incubated over night at 4°C. On the next day, cells were washed 3x with PBS, incubated with secondary antibody 1:500 for 1 h at RT, and washed again with 3x PBS. If a Hoechst (H3570, Life Technologies) staining was performed, cells were incubated for 5 min with 1:5000 Hoechst in PBS, then washed again 3x with PBS. Afterwards, cells were washed 3x with water and mounted on a slide with Aqua-Poly/Mount. Primary and secondary antibodies used in this study are listed in detail in the Supplementary Information.

### Karyotyping

Chromosome analysis was performed according to standard cytogenetic methods by treatment with trypsin, followed by Giemsa staining, in the Cytogenetics Laboratory of the Institute of Human Genetics (Heidelberg, Germany). Karyotype description was done according to the International System for Cytogenetic Nomenclature (2014). At least 10 metaphases were analysed for each clone.

### PCR, RT-PCR and Sequencing

DNA was isolated by using the Quick-DNA Miniprep Plus Kit (D4068, Zymo Research). PCR was performed with HotStarTaq polymerase (203205, Quiagen). Primers were designed by using NCBI Primer blast and can be found in the Supplementary information. Samples were sequenced by Azenta Life Science. To analyse the different *KCNQ1* transcripts (Supplementary Fig. 8), a regular PCR was performed. Instead of using DNA, transcribed cDNA was used as PCR input to only amplify expressed transcripts on mRNA and not DNA level.

### Trilineage Differentiation and analysis

To differentiate iPSCs into all three germ layers, a Trilineage Differentiation Kit (05230, StemCell Technologies) was used. After harvesting the cells, RNA was isolated and transcribed into cDNA. This cDNA was used to measure specific marker genes for each germ layer in iPSCs and the respective differentiation. Primer sequences are provided in the Supplementary information. Expression in differentiated cells was normalized to iPSC expression.

### NSC induction

NSCs were generated by using the protocol of Yan et al.^33^. IPSCs were split with Accutase and 300.000 cells/6-well were seeded in mTeSR Plus medium. 24 h after the split, medium was changed to PSC Neural Induction medium (A1647801, Thermo Fisher, 98% neurobasal medium, 2% neural induction supplement (NIS)). On day 7 of induction, cells were split with a high cell density (3 million cells/6-well) in neural expansion medium (NEM) (Neurobasal, Advanced DMEM/F12 (12634010, Thermo Fisher), PenStrep (15140122, Thermo Fisher), NIS) and ROCK inhibitor for 24 h. After this split, these induced NSCs at passage 1 (P1) were further maintained in NEM up to P10 with minimal spontaneous differentiation (Supplementary Fig. 4a). Whenever cells reached 100% confluency (every 4-5 days), they were split with Accutase. ROCK inhibitor was used from P1 to P4. From P5 till P10 onwards cells were used for experiments.

### Neuronal differentiation

Neurons were generated by using the protocol of Yan et al^33^. with some modifications. For differentiation, NSCs were first maturated for 6 days and then differentiated into cortical neurons for 6 weeks (Supplementary Fig. 5a, b). For the maturation, NSCs were seeded in 6-well format on Geltrex coating in NEM medium. After 24 h, medium was changed to maturation medium (MM) (50% Neurobasal medium, 50% Advanced DMEM/F12, 1% Glutamax (35050061, Thermo Fisher), 1% N2 (17502048, Thermo Fisher), 0.5% B27 (17504044, Thermo Fisher) and 1% PenStrep) and after 6 days, cells were seeded for neuronal differentiation on Ornithin- (P4957, Sigma Aldrich) Laminin (L2020, Sigma Aldrich) coating, either in 6-well format or 24-well format with cover slips, depending on the follow-up experiment. Again, 24 h after the split, medium was changed to neuronal differentiation medium (NDM) containing Neurobasal Plus, 2% B-27 plus (both A3653401, Thermo Fisher), 1% Glutamax, 0.1% ascorbic acid (A5960, Sigma Aldrich) and 1% PenStrep. We performed a MitomycinC (M0503, Sigma Aldrich) treatment when cells reached 90-100% confluency (usually on day 6 of differentiation, 10 µg MitomycinC for 10 min). Cells were always differentiated for 42 days in NDM and then used for analysis. This protocol was used for all experiments investigating neurons.

An alternative protocol for neuronal differentiation was used to confirm a role of KCNQ1 in neurite outgrowth which was modified according to Qi et al^34^. Unlike the protocol from Yan et al^33^., which involved the generation and expansion of NSCs prior to neuronal differentiation, the Qi et al. method induces rapid cortical neuron differentiation directly from iPSCs using small-molecule inhibitors, bypassing the NSC stage. Cells differentiated via the Qi protocol were assessed for neurite outgrowth after splitting on day 8 of differentiation. Further details are given in the Supplementary information.

### qPCR analysis

qPCR primer efficiency was determined by performing qPCRs on 3-fold serial dilutions of cDNA (1:1 to 1:81) and calculating efficiency (%) using Quant studio (version 6, Thermo Scientific). Primer pairs with efficiency 90-100% were selected for subsequent qPCR experiments. Data were analyzed using the 2-ΔΔCt method and presented as log2 fold change (log2FC) relative to the WT control (hetKO 3, homKO 1 vs. WT 1, hetKO 1, hetKO 2 vs. WT 2). Log2FC was calculated per replicate against the mean of the respective WT sample. Statistical analysis was carried out on log2FC values. Each dot represents one biological replicate. Bars indicate mean ± SD. Primer sequences can be found in the Supplementary Information.

### Mouse Prefrontal Cortex Samples

For developmental expression analyses, previously collected RNA samples from the prefrontal cortex of male wild-type CD1 mice were used. Samples were obtained at embryonic stages E15 and E17, postnatal stages P1 and P7, and adulthood (12 weeks of age; n = 4 animals per developmental stage) from a previously published developmental cohort48. *Kcnq1* expression was quantified relative to the reference genes *Hprt* and *Sdha*.

For protein expression analyses, dissected prefrontal cortex tissue from adult (25-week-old) wild-type, heterozygous, and homozygous *Kcnq1* knockout mice, all on a C57BL/6 genetic background, was provided by Dr. Jeffrey Glennon (Conway Institute of Biomolecular and Biomedical Research, University College Dublin, Dublin, Ireland). Ethical approval for the breeding and experimentation with the constitutive KCNQ1 knockout mouse was granted by the Animal Research Ethics Committee (AREC) of University College Dublin (protocol code Glennon-21-08), and the Irish national regulatory agency governing procedures with animals, namely the Health Products Regulatory Authority (HPRA) under the authorization number AE18982/P204. Protein lysates were prepared from the supplied tissue samples in our laboratory and subsequently analyzed by Western blotting.

### Neurite outgrowth and treatments

To measure neurite outgrowth, 120.000 NSCs were seeded in a 24-well on Ornithin-Laminin coating in NDM and immediately placed into the Incucyte S3 Live-cell Analysis Instrument system (Sartorius). In case of treatments, NDM contained either JNJ303 (1 µM; 3899, Tocris) or ML277 (1 µM or 2 µM; 4777, Tocris). For the mock control treatment, DMSO was used, as both JNJ303 and ML277 were diluted with DMSO. One hour after cell seeding, the first measurement was conducted. Cells were afterwards imaged every 4 hours for 48 hours. Neurite length was determined with the Neurotrack software and normalized to cell body cluster area. Each measured time point was normalized to the first measurement (time point 0) which was set to 1. When comparing the KO to its respective WT control, each time point of the KO line was normalized to the respective time point of the WT control, which was also set to the value 1. The same procedure was used for treatment experiments, were mock treatment of each cell lines served as control and was set to 1, and treatment conditions were analysed in relation to mock treatment of the same cell line. The area under the curve was calculated based on the normalized data.

### Analysis of proliferation and apoptosis

To assess the proliferation and apoptosis rate of NSCs, cells were split with Accutase and 25.000 cells were seeded in a 96-Well on Geltrex coating in NEM. 16 h after seeding the cells, they were treated with two different dyes simultaneously: Nuclight Rapid Red Dye (4717, Sartorius) to examine proliferation and Annexin V Dye (4642, Sartorius) to examine apoptosis. Additionally, cells were treated either with JNJ303 (1 µM), ML277 (1 µM, 2 µM) or DMSO (mock). Immediately after treatment, cells were placed into the Incucyte system and the recording was started within 30 min. Cells were recorded for 48 h every 2 h. To analyse the apoptosis rate, values were normalized to the number of cells (determined by Nuclight Red Dye). Proliferation and apoptosis rate were normalized to timepoint 0 h. The area under the curve (AUC) of cell numbers and of the apoptosis rate over 48 h was calculated.

### Western blotting

Protein was isolated with Pierce RIPA Buffer (89900, Thermo Scientific) containing protease inhibitor cocktail (PIC) (S8820, Sigma Aldrich) and PhosSTOP (4906845001, Sigma Aldrich). After incubation for 5 min on ice, benzonase (70746-4, Sigma Aldrich) was added and incubated for 10 min on ice. Afterwards, samples were centrifuged at 13.000 rpm at 4°C for 45 min. The supernatant was transferred to a fresh tube, as the final protein lysate. Protein concentration was determined with a BCA Kit (23225, Thermo Scientific). Samples were denatured at 95°C for 5 min and loaded to a 4-12% Tris-Glycin gel (XP04120BOX, Thermo Scientific). Protein was transferred from the gel to a PVDF membrane with the iBlot 2 system (Invitrogen). Membranes were blocked in blocking buffer (Intercept Blocking Buffer by LI-COR (927-70001) + PBS) for 1 h at RT shaking. Primary antibody was incubated in blocking buffer over night at 4 C under constant shaking. On the next day, membranes were washed 3×10 min in PBS-T. Secondary antibody was incubated for 45 min at RT in blocking buffer and constant shaking, and afterwards removed by washing 3x in PBS-T. Primary antibodies used in this study were: Anti-KCNQ1 (abcam, ab84819, clone N37A/10, 1:500), Anti-AKT (Cell Signaling Technologies, #9272, 1:1000), Anti-p44/42 MAPK (ERK1/2)(Cell Signaling Technologies, #4696, 1:1000), Anti-Phospho-p44/42 MAPK (Erk1/2) (Thr202/Tyr204) (Cell Signaling Technologies, #4370, clone D13.14.4E, 1:1000), Anti-ATP5A (Abcam, Ab14748, clone 15H4C4, 1:1000), Anti-GAPDH (NovusBio, NB300-328SS, clone 2D4A7, 1:15000); Secondary antibodies used in this study were: Anti-Rabbit IRDye800CW (LI-COR, Cat. 926-32213, 1:15000), Anti-Mouse IRDye800CW (LI-COR, Cat. 926-32212, 1:15000), Anti-Rabbit IRDye680CW (LI-COR, Cat. 926-32223, 1:15000), Anti-Mouse IRDye680CW (LI-COR, Cat.926-32222, 1:15000). Membranes were imaged using the Odyssey Imaging System (LI-COR).

### Electrophysiology

#### General

Electrophysiological recordings were performed using a BX51 upright microscope (Olympus), equipped with differential interference contrast (DIC) and fluorescent capabilities. Electrical signals were recorded using a Multiclamp 700B amplifier (Axon Instruments) controlled by Clampex 10.1 and Digidata 1440 digitizer (Molecular Devices). Recordings were done at 26 ± 1°C using a dual TC344B temperature control system (Sutter Instruments), with neurons continuously perfused with oxygenated (95% O2 and 5% CO2) artificial cerebro-spinal fluid containing 125 mM NaCl, 2.5 mM KCl, 1 mM MgCl_2_, 2 mM CaCl_2_, 25 mM glucose, 1.25 mM NaH_2_PO_4_, 0.4 mM ascorbic acid, 3 mM myo-inositol, 2 mM Na-pyruvate, and 25 mM NaHCO3, pH 7.4. Neurons were approached and patched under DIC, using 3.0 ± 1.0 MegaOhm pipettes (WPI), pulled with a PC10 puller (Narishige, Japan). For current clamp experiments, pipettes were loaded with an internal solution containing (in mM) 140 K-gluconate, 20 KCl, 10 HEPES, 0.05 BAPTA, 10 ATP-Mg^2^ + , 0.3 GTP-Na + , 10 Na + -Phosphocreatine, osmolarity 312 mOsmol, pH 7.2 adjusted with KOH. For voltage clamp experiments, pipettes were loaded with an internal solution containing 125 mM Cs-gluconate, 20 mM KCl, 4 mM MgATP, 10 mM Na-phosphocreatine, 0.3 mM GTP, 0.5 mM EGTA, 2 mM QX314 (Hello Bio, #HB1030), and 10 mM HEPES, pH 7.2. Data analysis was done using Clampfit 10.5.

#### Intrinsic neuronal properties

To determine the basic electrophysiological properties of control and mutant iPSC-derived neurons, we performed whole cell current clamp recordings. In these experiments, cells were maintained at ∼-70 mV potentials by injecting small (< 30 pA) amounts of current through the recording pipette. Cells in which larger current injections were needed to keep the membrane potential at ∼-70 mV were discarded. To determine the rheobase, i.e. the minimal current needed to trigger an action potential (AP), cells were increasingly depolarized from their resting potential by injections of positive current (1 pA steps) for 500 ms. The AP properties (height, half-width, max speed) were extracted from spikes generated during rheobase measurements. To determine the input resistance and input-output function of the differentiated neurons, we used stepwise current injections through the recording pipette (500 ms pulses) from-100 to 480 pA, with 20 pA steps.

#### Spontaneous and miniature excitatory postsynaptic currents

To measure excitatory currents, we performed whole-cell voltage-clamp recordings at -70 mV holding potentials. Spontaneous excitatory currents were detected as downward deflections from the baseline. Miniature currents were recorded under identical conditions, but after addition of 0.5 μM tetrodotoxin to the bath, to block sodium channels and thus spike-dependent spontaneous excitatory transmission.

### RNA sequencing and data analysis

RNA isolation for RNA sequencing was carried out by GENEWIZ from Azenta Life Sciences. Frozen cell pellets of NSCs (2.5-3 million cells) from homKO 1 and WT 1 samples and cortical neurons from homKO 1, hetKO 1, hetKO 2, hetKO 3, WT 1, and WT 2 were provided.

#### RNA sequencing

Library preparation with poly(A) selection and 150-bp paired-end sequencing on an Illumina NovaSeq, as well as mapping sequence reads to the reference genome and extracting gene counts were performed by GENEWIZ (Germany). Specifically, sequence reads were trimmed to remove possible adapter sequences and nucleotides with poor quality using Trimmomatic v.0.36^48^. The trimmed reads were mapped to the Homo sapiens GRCm38 reference genome available on ENSEMBL using the STAR aligner v.2.5.2b^49^. Distinct gene hit counts were calculated by using featureCounts from the Subread package v.1.5.2^50^. The hit counts were summarized, and only unique reads that fell within exon regions were counted. Furthermore, counts were normalized according to the library size and filtered, keeping only genes having count per million reads (cpm) >0.5 across 4 samples for further analysis, as previously suggested^51^. Exploratory data analysis included principal component analysis (PCA) and correlation analysis to assess variability between samples and hierarchical clustering with heatmap using R package PCAtools^52^. For the differential gene expression analysis, we used R package DESeq2^53^ and performed pairwise comparisons between: (a) NSCs homKO 1 and WT 1 samples, (b) neuronal hetKO 1 and WT samples, (c) neuronal hetKO 2 and WT samples, (d) neuronal hetKO 3 and WT samples, (e) neuronal homKO 1 and WT samples. When testing for differential expression in neuronal cells, we adjusted for the effect of type of gRNA and differentiation batch, as these two factors showed influence on gene expression based on PCA plot Volcano plots and heatmaps were generated using Rstudio with ggplot2 package^54^. Venn diagrams were generated by InteractiVenn^55^.

#### Ingenuity Pathway Analysis (IPA)

We used IPA (Q2-2024) to analyse differentially expressed genes for enriched canonical pathways and networks (with up- and down-regulated genes analysed together). The background gene list included all genes that were detected to be expressed in each of the cell lines. We applied the Benjamini-Hochberg procedure for multiple hypothesis correction and used a *P*-adjusted threshold (FDR) of < 0.05 to denote significant findings.

### Liquid chromatography mass spectrometry (LC-MS)

#### Preparation of beads for SP3 protein digestion

Following vortexing, 80 µl Promega MPSP beads (cat. No CS3325A04) and 720 µl of dH_2_O were combined in a low-binding Eppendorf tube. The tube was placed on a magnetic rack allowing beads to settle for about 2 min. After the supernatant was removed, beads were rinsed three times with 800 µl of dH_2_O. Beads were prepared freshly.

#### Protein reduction, alkylation, and digestion using SP3 method

10 µg of protein extracts were digested using the SP3 method with some modifications^56^. Each protein extract was mixed with reduction/alkylation buffer (100 mM TEAB pH 8.5, 1% SDS, 10 mM TCEP, 40 mM CAA) and incubated at 95 C for 5 min and then 25 min at 70° C. After the samples were cooled down to RT, 2 µl of pre-rinsed beads (see above) were mixed in. Finally, acetonitrile (ACN) was added to reach 80% ACN. The samples were then mixed thoroughly and incubated for 20 min on a shaking incubator. Next, samples were shortly centrifuged, placed on a magnetic rack, and the supernatant was carefully removed. Beads were washed with 80% v/v EtOH for a total of three washes, and once with 80% v/v ACN. Beads were reconstituted in 100 µl of 100 mM TEAB, pH 8.5. Trypsin was added in a ratio of 1:50, followed by overnight incubation at 37° C. Samples were acidified using TFA to reach pH < 2. The supernatant was transferred to fresh vials. Digested proteins were desalted on self-assembled C18 Empore® extraction discs (3 M, Maplewood, MN) STAGE tips^57^.

#### LC-MS analysis and data processing

Samples were suspended in 0.1% TFA and an equivalent of 2.5 µg of peptides was analysed using an Ultimate 3000 liquid chromatography system coupled to an Orbitrap QE HF (Thermo Fisher) as described earlier^58^. Mobile phase solutions were prepared as follows, solvent A: 0.1% formic acid / 1% acetonitrile, solvent B: 0.1% formic acid, 89.9% acetonitrile. Shortly, peptides were separated in a 120 min linear gradient started from 3% solvent B and increased to 23% B over 100 min and then to 38% B over 20 min, followed by washout with 95% B. The mass spectrometer was operated in data-dependent acquisition mode, automatically switching between MS and MS2. MS spectra (m/z 400–1600) were acquired in the Orbitrap at 60,000 (m/z 400) resolution, and MS2 spectra were generated for up to 15 precursors with normalized collision energy of 27 and isolation width of 1.4 m/z.

#### LC-MS data analysis

The MS/MS spectra were searched against the H. sapiens (UP000005640) and a customized contaminant databases using Proteome Discoverer 2.5 with Sequest HT^59^. The fragment ion mass tolerance was set to 0.02 Da, and the parent ion mass tolerance to 5 ppm. Trypsin was specified as an enzyme. The following variable modifications were allowed: Oxidation (M), Deamidation (N, Q), Acetylation (N-terminus), Met-loss (M), and a combination of Met-loss and acetylation (N-terminus), whereas Carbamidomethylation (C) was set as a fixed modification. Peptide quantification was done using a precursor ion quantifier node with the Top N Average (n = 3) method set for protein abundance calculation. Data preprocessing was performed in R (v4.3.1). Protein identifiers were made unique using the “make_unique()” function from the DEP2 package^60^. Normalization was performed using the variance stabilizing normalization (VSN) method implemented in DEP2, adapted from the vsn package^61^. Missing values were imputed using the MinProb method (q = 0.01). Visualization was performed using PCA, volcano plots, k-means clustered heatmaps, and correlation matrices. Gene Set Enrichment Analysis (GSEA) was conducted using the clusterProfiler (v4.10.0) package^62–65^. Enrichment was performed with pvalueCutoff = 0.05 and without *p*-value adjustment (pAdjustMethod = “none”) unless otherwise stated. Visualization of enrichment results was carried out using enrichplot (v1.22.0) and DOSE (v3.28.1)^66^.

#### Ingenuity Pathway Analysis (IPA)

We used IPA (Q3-2025) to analyse differentially expressed proteins for enriched canonical pathways (with up- and down-regulated proteins analysed together). The background protein list included all proteins that were detected to be expressed in each of the cell lines. We used a non-adjusted *p* value threshold of < 0.05 to denote significant findings (for differentially expressed proteins and enriched pathways).

### Mitochondria copy number and deletion

To measure mitochondrial copy number, DNA was isolated with the Quick-DNA Miniprep Plus Kit (D4068, Zymo Research) and 8 ng of DNA was used to quantify mitochondrial DNA (mtDNA) encoding the genes NADH dehydrogenase 2 (*ND2*), cytochrome C oxidase 1 (*CO1*) and beta-2 microglobulin (*β2M*) on genomic DNA with the QuantStudio 3 Real-Time PCR System. We calculated mitochondrial DNA copy numbers using the mtDNA/nDNA (nuclear DNA) ratio. To measure mitochondria deletion, the quantity of the mitochondrial genes *ND2* and *CO1* were analysed in relation to NADH dehydrogenase 4 (*ND4*), because *ND4* is known to have a high susceptibility to deletions, while *ND2 and CO1* have not. Primer sequences are available in the supplementary information.

### Mitochondria morphology and area

Neural stem cells were stained with MitoTracker™ Red CMXRos (M7512, Thermo Fisher Scientific) and imaged by confocal fluorescence microscopy using identical acquisition settings for all samples. Mitochondrial area per nucleus, foreground length per nucleus, aspect ratio, and circularity were quantified using FIJI/ImageJ. Statistical analyses were performed in R using linear mixed-effects models with genotype as a fixed effect and biological replicate as a random intercept. Significance was assessed by Type III ANOVA with Satterthwaite’s approximation, followed by Tukey-adjusted pairwise comparisons.

### nCounter® gene expression profiling

To validate the RNA Sequencing results, 16 DEGs were selected for follow-up analysis using nCounter direct RNA quantification (selected genes were highlighted in bold in Supplementary Fig. 23c). Genes were selected based on the following criteria: differential expression in all or at least three *KCNQ1*-KO lines, or differential expression in two cell lines with highest and lowest log2fold changes. Genes with very similar or unclear function were excluded (Suppl. Table 1 Tab 7).

The same batch of RNA as for RNA sequencing was used. After quantity and quality control of RNA samples, an nCounter® target gene expression analysis was performed at the nCounter Core Facility (Institute of Human Genetics, Heidelberg University Hospital, Germany, https://t1p.de/nCounter-Core-Facility) using a customized nCounter® Elements TagSet (https://nanostring.com/products/

ncounter-assays-panels/ncounter-custom-solutions/elements-tagsets/) containing 16 target genes and 6 reference genes (TagSet probe design, detailed probe information is provided in the Supplementary Information). Briefly, for each hybridization reaction, 50 ng of total RNA was used to hybridize the probe set at 65°C. Up to 7 μl of total RNA samples were combined with 2 μl nCounter customized TagSet, 5 μl hybridization buffer, and 0.5 μl probe A plus 0.5 µl probe B for a total reaction volume of 15 μl. Samples were incubated for 20 h, cooled to 4°C and then purified, immobilized on a cartridge, and measured using the nCounter® SPRINT profiler (NanoString a Bruker Company, Seattle, WA, USA, www.nanostring.com).

Normalization and analysis of the raw data was performed using the internal positive controls and the stably expressed reference genes with the freely available nSolverTM Analysis Software (version 4.0, https://www.nanostring.com/products/analysis-software/nsolver). Stably expressed reference genes were selected based on the Normfinder method (v0.953, MS Excel Add-in66). To determine the background and detection limit, the threshold was set as the geomean of negative controls (NEG_A-F, included in the assay by default) + 2*standard deviation. Gene numbers below this threshold were considered as background, those above as specific signal. Genes exceeding this threshold in at least 50% of the samples within a group were considered to be present in the respective group. Genes not present within all groups were removed from the downstream analysis.

### Statistics and Reproducibility

Data normality was assessed using the Shapiro–Wilk test. Homogeneity of variances was evaluated by visual inspection and comparison of standard deviations between groups. For datasets with equal variances, one-way ANOVA followed by Bonferroni-adjusted post hoc testing was applied. When variances were unequal ( > 3-fold difference in standard deviations), Welch’s ANOVA with Dunnett’s T3 post hoc correction was used. Pairwise comparisons were performed using two-tailed Student’s t-tests or Welch’s t-tests, as appropriate. For the qPCR gene panel, p-values from pairwise comparisons (two-sided Welch’s t-tests; n = 36 tests) were adjusted using the Benjamini–Hochberg false discovery rate (FDR). Neurite length was normalized to baseline per well and analyzed using linear mixed-effects models (LMM). Fixed effects included genotype, treatment, time, and their interactions. Well identity (random intercept and slope for time) and experiment (random intercept) were included as random effects. Genotype-dependent effects were assessed via genotype × time interactions, and treatment effects via genotype × treatment × time interactions. LMM analyses were performed in Python (v3.11) using the statsmodels MixedLM module. AUC of neurite outgrowth was analyzed using a one-sample t-test against 1, followed by Bonferroni correction. To analyse neuronal activity (voltage responses upon injections of hyperpolarizing current injections, spiking activity upon injections of depolarizing current injections), a two-way ANOVA was used with the factors cell line and injected current followed by a Bonferroni-adjusted post hoc test. To analyse AP properties, rheobase, and EPSC properties, an unpaired, two-tailed Student’s *t*-test was performed. For nCounter analysis, multiple unpaired two-tailed Student’s *t*-test were used with two-stage step-up method of Benjamini, Krieger and Yekutieli (FDR = 0.05) to correct for multiple testing. Statistical analysis was performed using GraphPad Prism 10.3.1 (GraphPad Software, San Diego, CA, USA), except for the LMM.

For the transcriptomic data analysis, *p*-values were adjusted for multiple testing by DESeq2 using the Benjamini-Hochberg procedure with independent filtering disabled. Genes having an adjusted *p* < 0.05 were considered differentially expressed.

For the proteomic data analysis, differential expression analysis was carried out using linear modelling via the “test_diff()” function in DEP2, which internally applies empirical Bayes statistics based on the limma package^67^. Adjusted p-values were calculated using the Benjamini-Hochberg false discovery rate (FDR) correction. Proteins were considered significantly differentially abundant using thresholds of adjusted *p*-value < 0.1 and absolute log2 fold-change >0.58.

## Supplementary information

Supplementary Information

Supplementary Table 1

Supplementary Table 2

Supplementary data table

## Data Availability

The source data underlying Figs. 1–6 are provided in the Supplementary Data Table containing the source data for the main Figures, in the Supplementary Information, in Supplementary Table 1**. Differential gene expression, pathway analysis, and nCounter validation in** ***KCNQ1*****-KO NSCs and neurons**, and in Supplementary Table 2**. Differential protein expression and pathway enrichment analyses in** ***KCNQ1*****-KO NSCs and neurons**. The original western blot images are provided in the Supplementary Information. For each western blot presented in the main manuscript, the corresponding uncropped image is indicated by its respective Supplementary Fig. number. The processed and raw FASTQ gene-expression data generated from NSCs and neurons are available in the Gene Expression Omnibus under accession number GSE337397. The corresponding NSC and neuronal proteomics data have been deposited in the PRIDE database via ProteomeXchange under accession number PXD068563.
