# Supplementary material for: KCNQ1 disruption alters human neuronal development through mitochondrial and insulin signalling pathways
Source: Commun Biol. 2026 Oct 13;9:1341. doi: 10.1038/s42003-026-11111-0 (PMC13661531; doi:10.1038/s42003-026-11111-0)
Supplement: Supplementary file 1 [file 42003_2026_11111_MOESM1_ESM.pdf]

Supplementary Information

Supplementary Figures

Supplementary Materials and Methods

KCNQ1 disruption alters human neuronal development through mitochondrial and insulin signalling pathways

Dorothea Schall (1), Baran E. Güler (1), Siamand Alibrahim (2), Mahmudul Hasan (1), Naeem Ahmad (1, 3), Joanna Widomska (4, 5), Claudio Acuna (6), Mairéad Sullivan (7), Ralph Röth (1, 8), Chang Liu (1), Linda Blaier (1), Katalin Vincze (9, 10), Karin Burau (1), Simone Macri (11), Daniela Mauceri (12, 13), Marcin Luzarowski (14), Ágota Apáti (10), János M. Réthelyi (9), Geert Poelmans (4, 5), Gudrun A. Rappold (1), Barbara Franke (4, 5, 15), Jeffrey C. Glennon (7), Simone Berkel (1, 16)

Affiliations
(1) Institute of Human Genetics, University Hospital Heidelberg, Heidelberg, Germany
(2) Pharmaceutical Biotechnology, Department of Pharmacological and Biomolecular Sciences, University of Milan La Statale, Milan, Italy
(3) National Institute for Biotechnology and Genetic Engineering-College, Pakistan Institute of Engineering and Applied Sciences (NIBGE-C, PIEAS), Jhang Road, Faisalabad, Pakistan
(4) Department of Medical Neuroscience, Radboud University Medical Center, Nijmegen, The Netherlands
(5) Donders Institute for Brain, Cognition and Behaviour, Radboud University, Nijmegen, The Netherlands
(6) Chica and Heinz Schaller Foundation, Institute of Anatomy and Cell Biology, Heidelberg University, Germany
(7) Conway Institute of Biomolecular and Biomedical Research, School of Medicine, University College Dublin, Dublin, Ireland
(8) nCounter Core Facility, Institute of Human Genetics, Heidelberg University Hospital, Germany
(9) Department of Psychiatry and Psychotherapy, Semmelweis University, Budapest, Hungary
(10) HUN-REN, Research Centre for Natural Sciences, Budapest, Hungary
(11) Centre for Behavioural Sciences and Mental Health, Istituto Superiore di Sanità, Rome 00161, Italy
(12) Department of Neurobiology, Interdisciplinary Centre for Neurosciences (IZN), Heidelberg University, Germany
(13) Institute of Anatomy and Cell Biology, Dept. Molecular and Cellular Neuroscience, University of Marburg, Germany
(14) Core Facility for Mass Spectrometry and Proteomics, Center for Molecular Biology of Heidelberg University (ZMBH), DKFZ-ZMBH Alliance, 69120 Heidelberg, Germany
(15) Department of Human Genetics, Radboud University Medical Center, Nijmegen, The Netherlands
(16) Institute of Human Genetics, Heidelberg University, Heidelberg, Germany

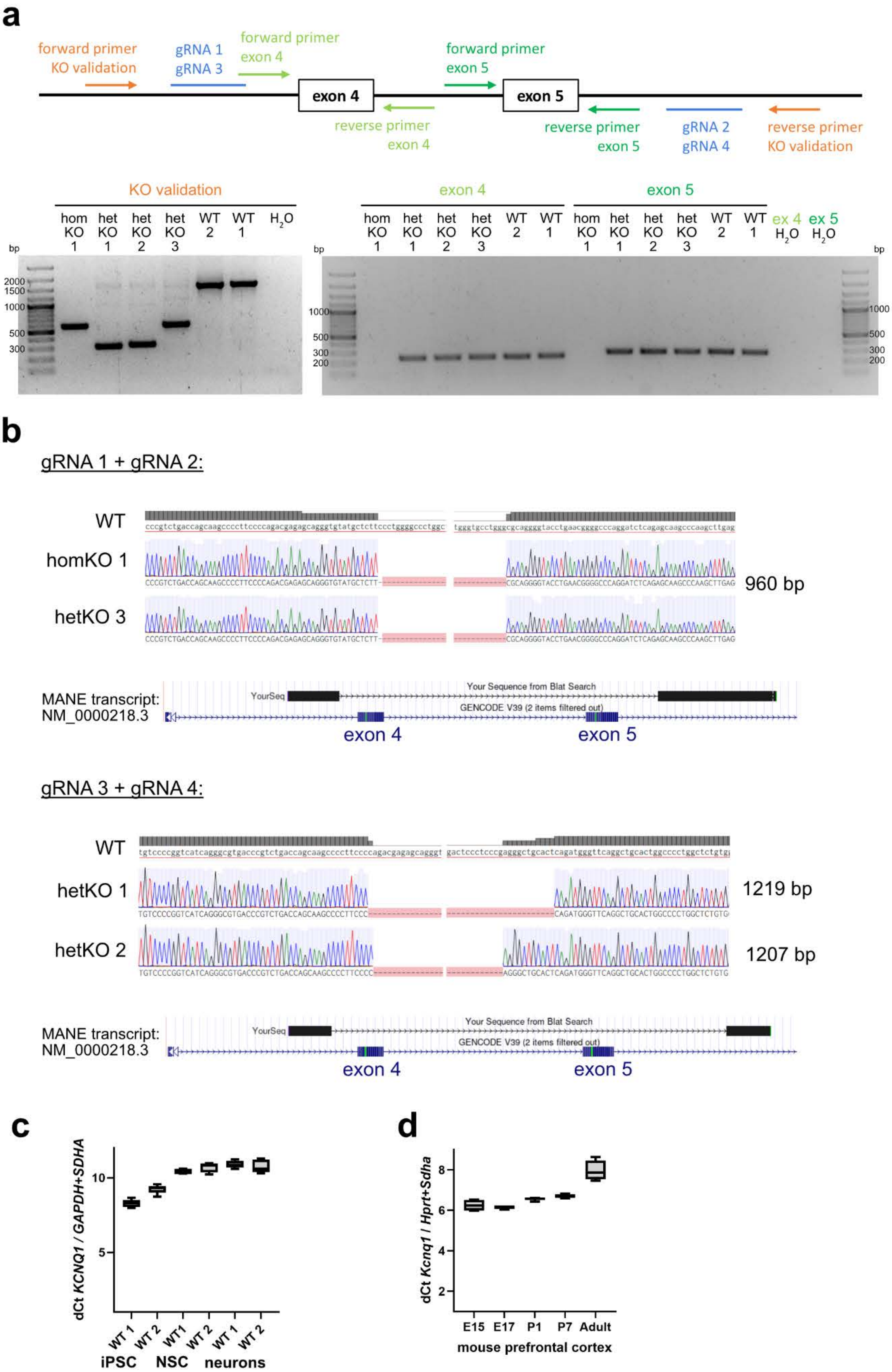
a
forward primer KO validation
gRNA 1
gRNA 3
forward primer exon 4
exon 4
reverse primer exon 4
forward primer exon 5
exon 5
reverse primer exon 5
gRNA 2
gRNA 4
reverse primer KO validation
KO validation
hom KO 1
het KO 1
het KO 2
het KO 3
WT 2
WT 1
H2O
bp
2000
1500
1000
500
300
exon 4
exon 5
ex 4 H2O
ex 5 H2O
200
b
gRNA 1 + gRNA 2:
WT
homKO 1
hetKO 3
960 bp
MANE transcript: NM_0000218.3
YourSeq
Your Sequence from Blat Search
GENCODE V39 (2 items filtered out)
exon 4
exon 5
gRNA 3 + gRNA 4:
WT
hetKO 1
hetKO 2
1219 bp
1207 bp
MANE transcript: NM_0000218.3
c
dCt KCNQ1 / GAPDH+SDHA
10
5
WT 1
WT 2
iPSC
NSC
neurons
d
dCt Kcnq1 / Hprt+Sdha
8
6
4
2
E15
E17
P1
P7
Adult
mouse prefrontal cortex

Supplementary Figure 1. Validation of *KCNQ1*-KOs in iPSCs. a PCR was used to screen and validate the *KCNQ1*-KO in the iPSC lines. The upper scheme shows where primers bind in relation to exons 4 and 5 and the gRNAs. For a first KO validation, a primer pair flanking the gRNAs upstream and downstream were used. Without a deletion the PCR product results in 1520 bp (WT cell lines). With a deletion, the PCR product for gRNA 1 + gRNA 2 (homKO 1, hetKO 3) results in a product size of 558 bp; and gRNA 3 + gRNA4 (hetKO 1, hetKO 2) results in a product size of 312 bp. In heterozygous KOs WT bands were faint as the smaller PCR products were preferentially amplified, therefore we additionally performed a PCR with specific primers for either exon 4 or 5. The homozygous KO does not show any band, whereas all heterozygous KOs and WT lines show a band for both exons, as expected. Band sizes: exon 4: 235 bp, exon 5: 268 bp. **b** Sanger sequencing results of KO cell lines. For each gRNA combination the upper picture shows sequencing alignment of KO compared to WT sequence to show the exact cutting side of each gRNA and the deletion size. The lower picture visualizes the deletion position on the *KCNQ1* gene by comparing the sequence to the MANE *KCNQ1* transcript (NM_000218.3, UCSC browser, hg38). Chromosome positions of deletions: homKO 1/hetKO 3: 2.571.269-2.572.229, hetKO 1: 2.571.424-2.572.461, hetKO 2: 2.571.243-2.572.450. **c** *KCNQ1* transcript levels relative to *GAPDH* and *SDHA* in iPSCs, NSCs and neurons. **d** Expression analysis of *Kcnq1* in the developing mouse (CD1) prefrontal cortex. *Kcnq1* expression relative to the reference genes *Hprt* and *Sdha*. For expression analyses during brain development, previously collected RNA from male wild-type CD1 mouse prefrontal cortex samples was used. Samples originated from an earlier study (PMID: 30319350) and comprised embryonic stages E15 and E17, postnatal stages P1 and P7, and adult animals (12 weeks; n = 4 per stage).

Unmodified gel picture 1a (left):

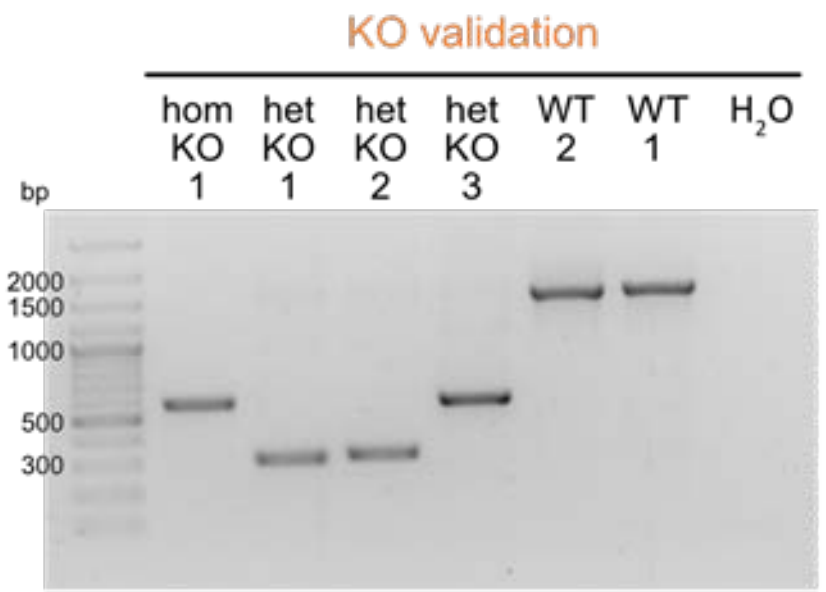

The gel image was processed by applying a uniform linear adjustment to the entire image, consisting of a 45% reduction in brightness and a 75% increase in contrast.

Experiments 1 - 3 for WT1, hetKO 3 & homKO1

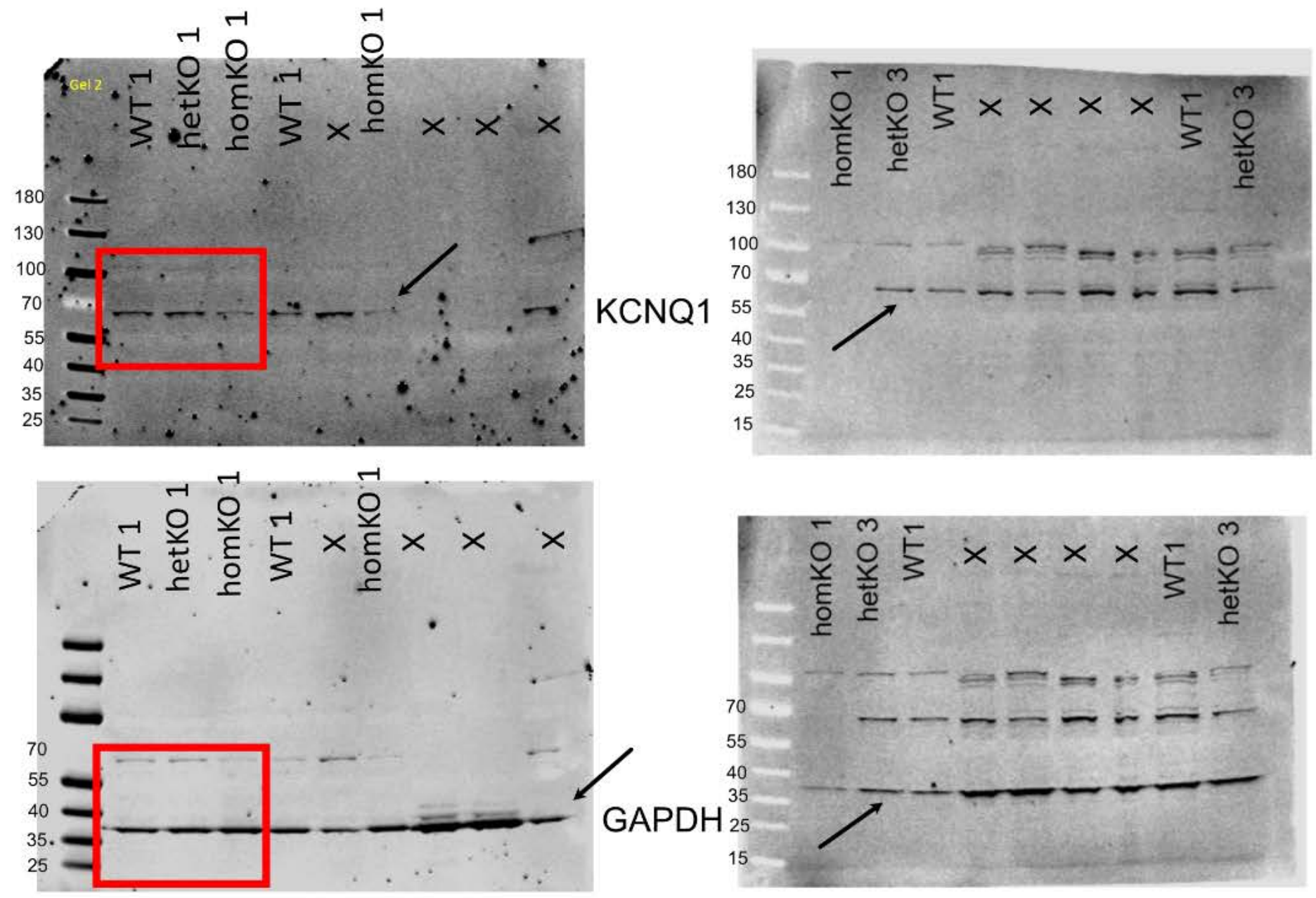

Experiments 1 - 3 for WT2, hetKO 1 & hetKO 2

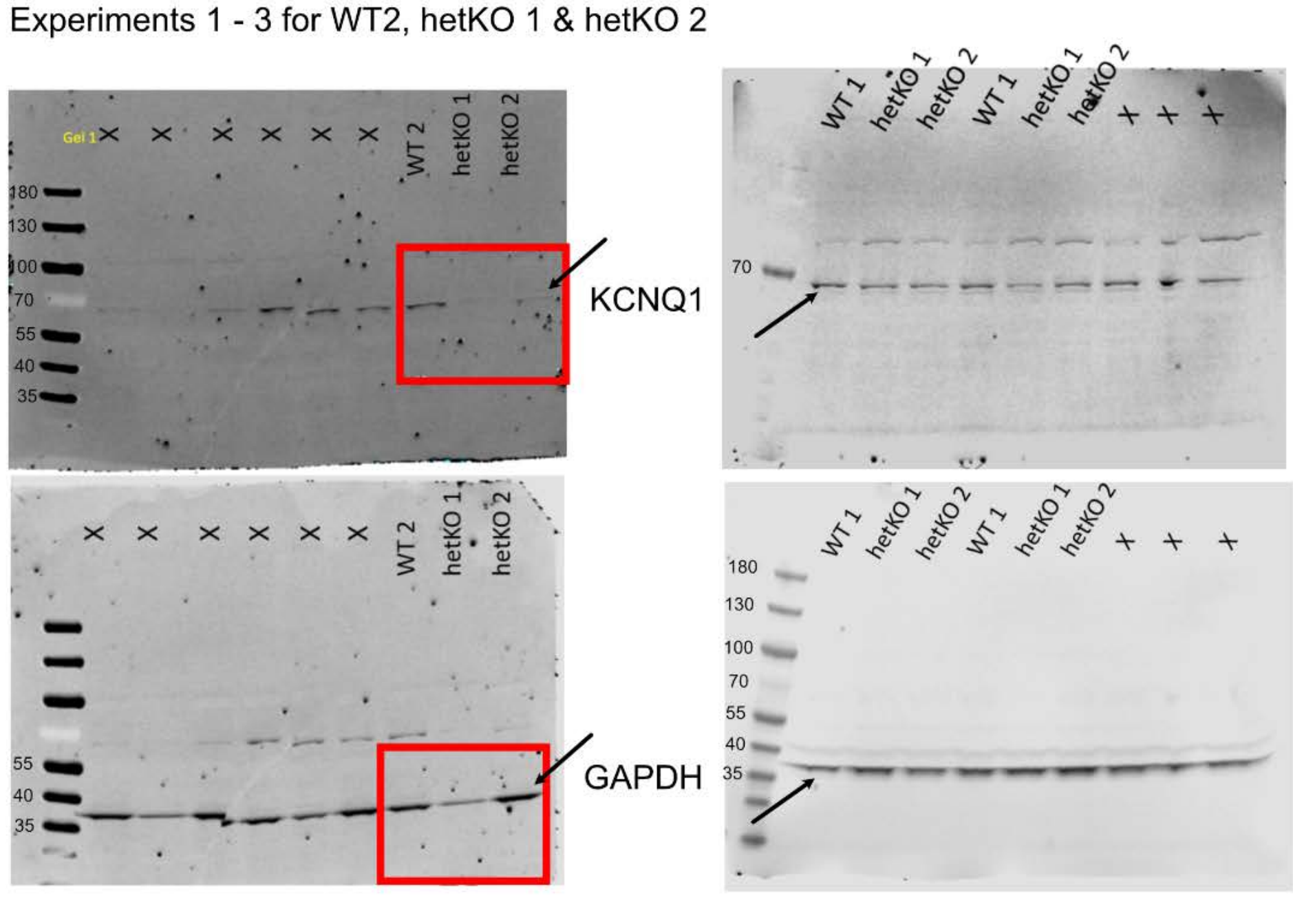

Supplementary Figure 2. Uncropped Western blot pictures linked to Figure 1d. Quantification of KCNQ1 and GAPDH. Molecular weight marker: page ruler prestained protein ladder (10-180 kDa, Thermo scientific). X – not used cell lines. Red boxes – cropped data in main figure.

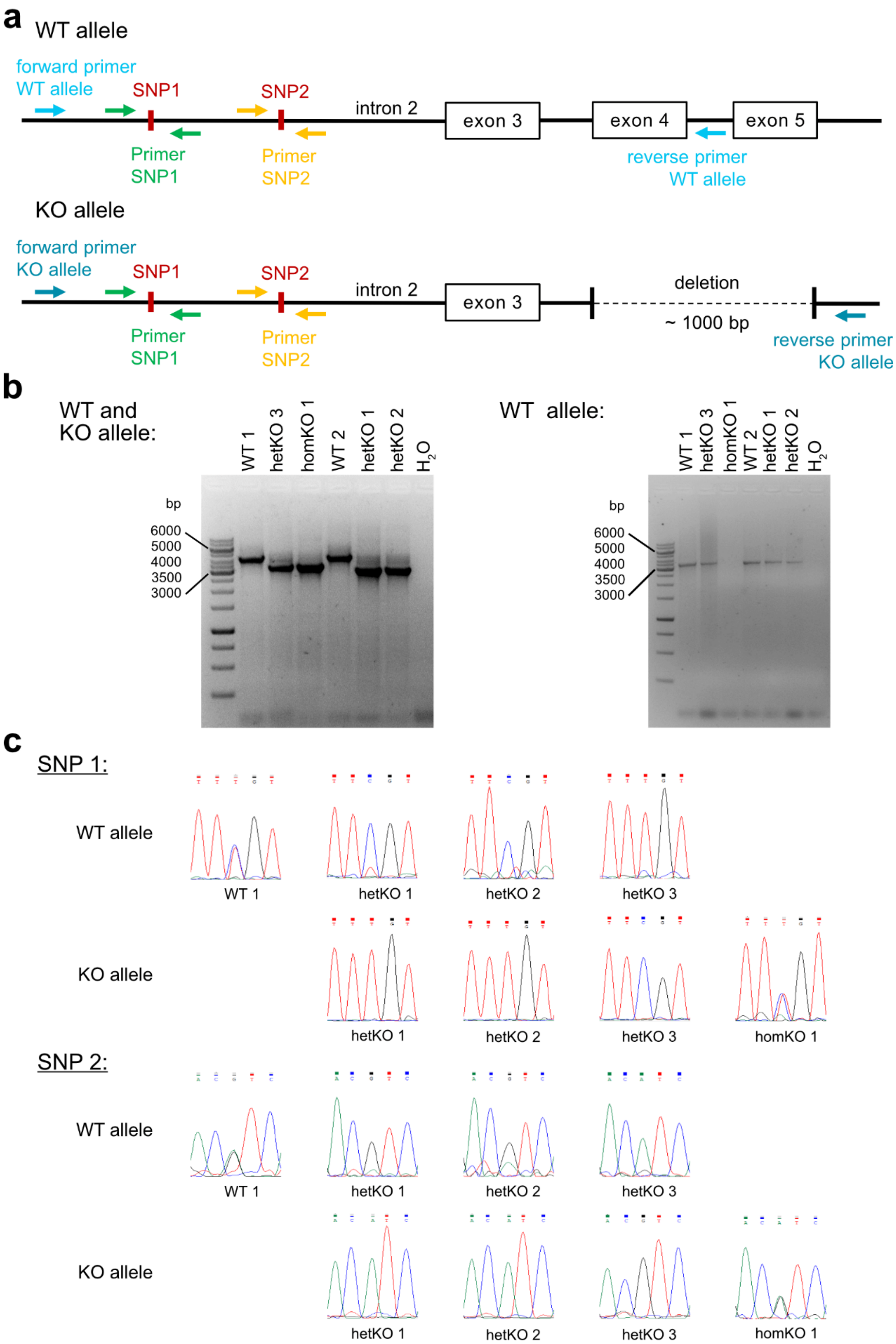

Supplementary Figure 3. Assignment of deletion to allele of the heterozygous KO cell lines. To distinguish which allele was edited in the heterozygous *KCNQ1*-KO iPSC lines, two heterozygous SNPs

in intron 2 (rs179484, rs179486) of *KCNQ1* were genotyped by Sanger sequencing. **a** Primer design for PCR and sequencing. For the specific amplification of the WT allele, the reverse primer was set within the region of the deletion. To amplify the KO allele, the reverse primer was set downstream of the deletion. Two specific primer pairs were designed to amplify and sequence the region of the two SNPs. **b** PCR amplification using primers designed for the WT allele amplified both WT (4191 bps) and knockout allele (~3100 bps) bands in heterozygous KO lines with preferential amplification of the shorter fragment. Specific amplification of the WT allele yielded a band of 3384 bp. Representative Sanger sequencing results are shown for PCR products detecting the WT allele or the KO allele in the respective iPSC lines. In WT lines and in the homozygous KO line, the SNPs remain heterozygous, indicating that both alleles are amplified under these conditions (in WT lines only WT alleles, in homozygous KO only KO alleles). In all heterozygous cell lines only one SNP variant per allele is visible, proving that with each PCR one allele is predominantly amplified, either WT or KO allele. SNP 1 (rs179484) displays a shift from C to T with C as the major and T as the minor allele. SNP 2 (rs179486) shifts from G to A with G as the major and A as the minor allele. The major allele was defined as allele A, the minor allele was defined as allele B. HetKO 1 and 2 carry the deletion on allele A, hetKO 3 carries the deletion on allele B.

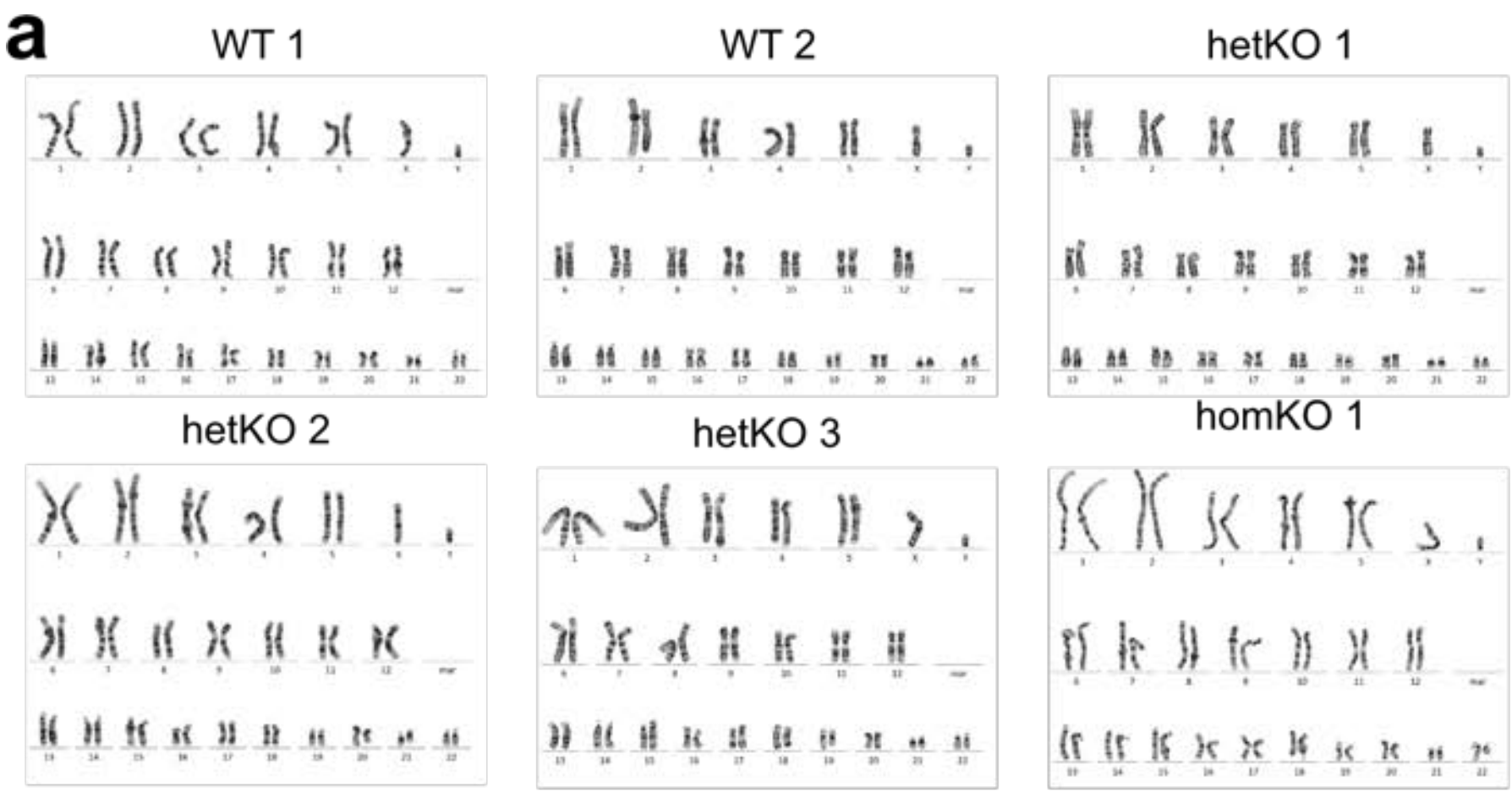

b

gRNA	Predicted Gene	Predicted Position	Off-target Editing
WT 1, homKO 1, and hetKO 3			
gRNA 1	*RPS19*	Exonic	no
	ELAV4	Exonic	no
	NES	Exonic	no
	CLDN9	Intronic	no
gRNA 2	*PCBD1*	Exonic	no
WT 2, hetKO 1, and hetKO 2			
gRNA 3	*FAM20C*	Exonic	no
	ADAM15	Intergenic	no
	ELOCP20	Intergenic	no
	MCF2L2	Intronic	no
	LARGE	Intronic	no
	PKHD1	Intronic	no
gRNA 4	*RAD51B*	Exonic	no
	PRR5L	Exonic	no
	CCDC57	Exonic	no
	THEM6	Intronic	no

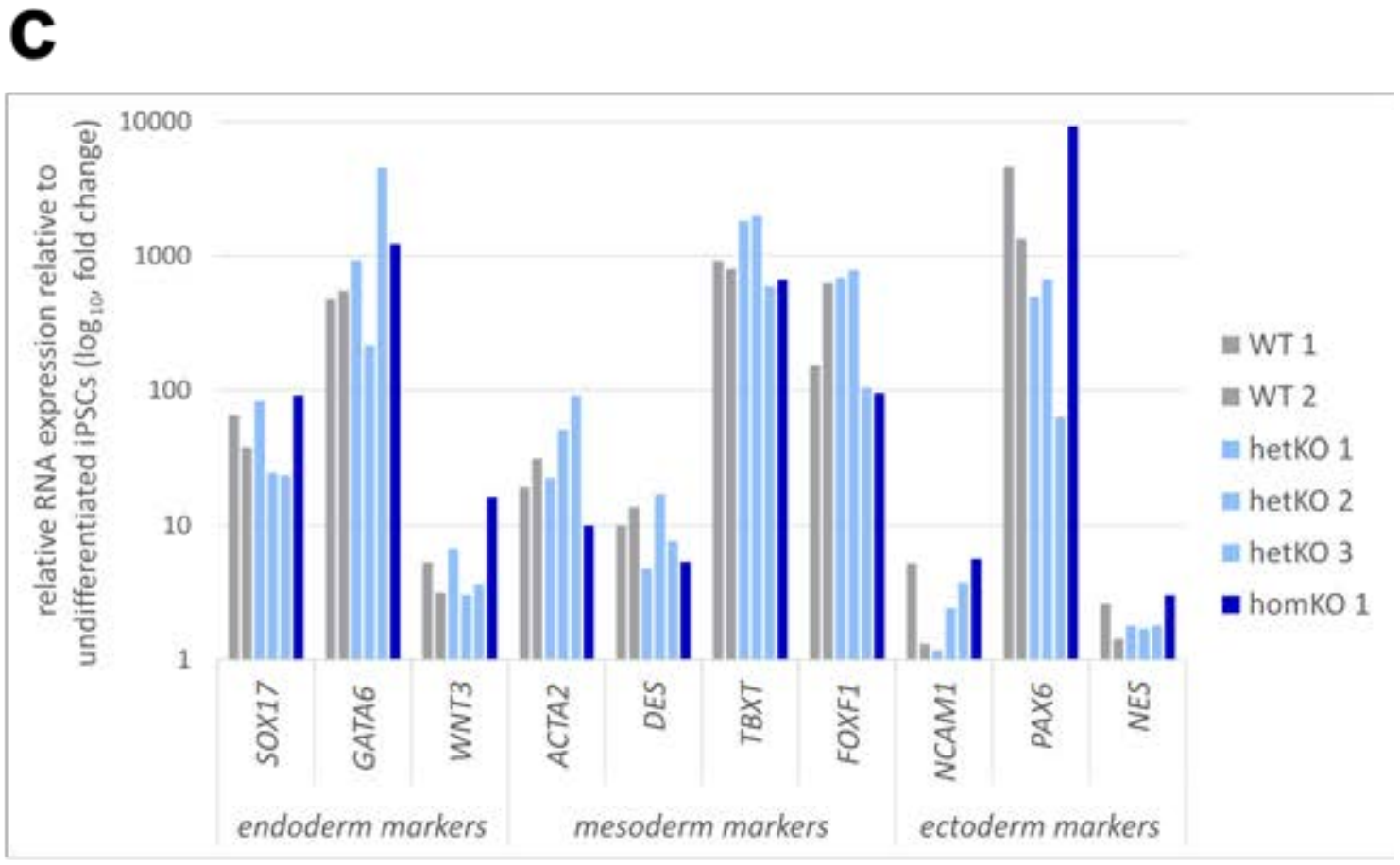

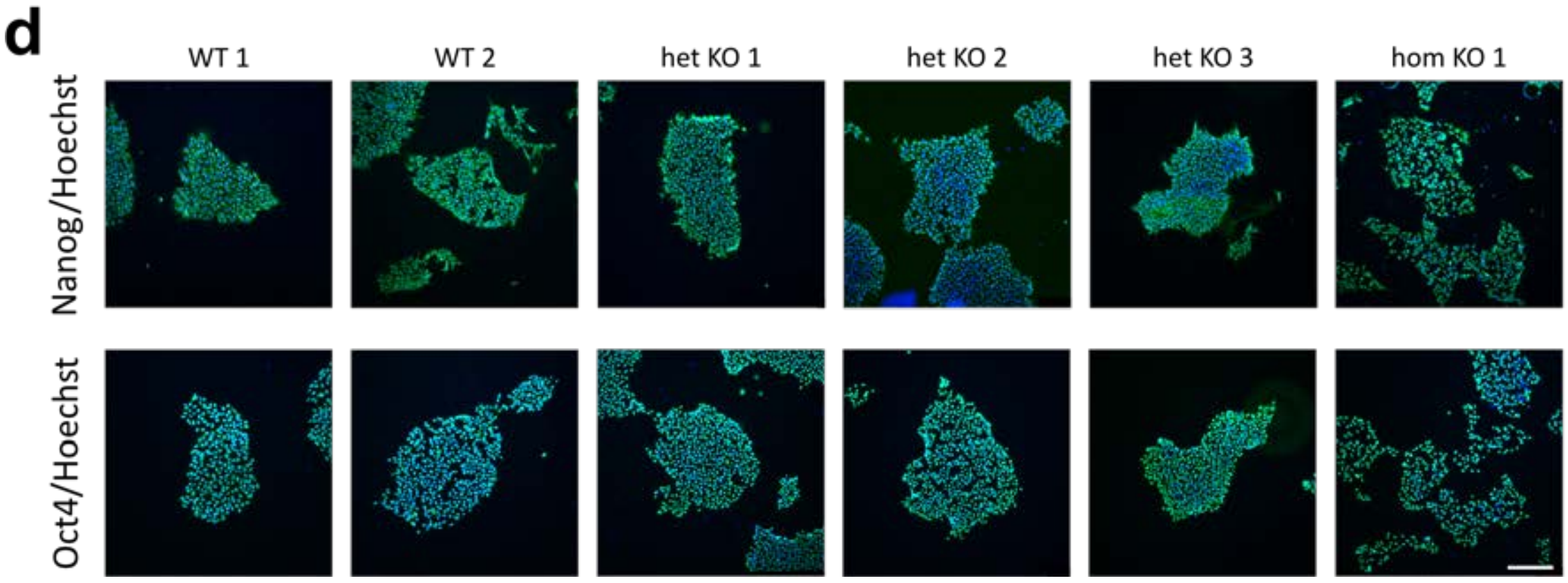

Supplementary Figure 4. Quality assessment of generated iPSC lines. a Karyotype images of all iPSC lines. **b** Sequencing of predicted putative off-target sites revealed no evidence for unspecific editing. **c** Relative RNA expression of endoderm, mesoderm, and ectoderm markers after trilineage differentiation of iPSCs. All generated cell lines show a higher expression of the germ layer specific markers relative to their respective undifferentiated iPSCs. **d** Immunofluorescence images of pluripotency markers. All iPSC lines express both pluripotency markers, Nanog or OCT4 (green); nuclei staining: Hoechst (blue). Scalebar = 100 µm. *RPS19*: ribosomal protein S19, *ELAV4*: ELAV like RNA binding protein 4, *NES*: nestin, CLDN9: claudin9, *PCBD1*: pterin-4 alpha-carbinolamine dehydratase 1, *FAM20C*: FAM20C Golgi Associated Secretory Pathway Kinase, *ADAM15*: ADAM metallopeptidase domain 15, *ELOCP20*: elongin C pseudogene 20, *MCF2L2*: MCF.2 cell line derived transforming sequence-like 2, *PKHD1*: PKHD1 ciliary IPT domain containing fibrocystin/polyductin, *RAD51B*: RAD51

paralog B, *PRR5L*: proline rich 5 like, *CCDC57*: coiled-coil domain containing 57, *THEM6*: thioesterase superfamily member 6, *SOX17*: SRY-box transcription factor 17, *GATA6*: GATA binding protein 6, *WNT3*: Wnt family member 3, *ACTA2*: actin alpha 2, smooth muscle, *DES*: desmin, *TBXT*: T-box transcription factor T, *FOXF1*: forkhead box F1, *NCAM1*: neural cell adhesion molecule 1, *PAX6*: paired box 6.

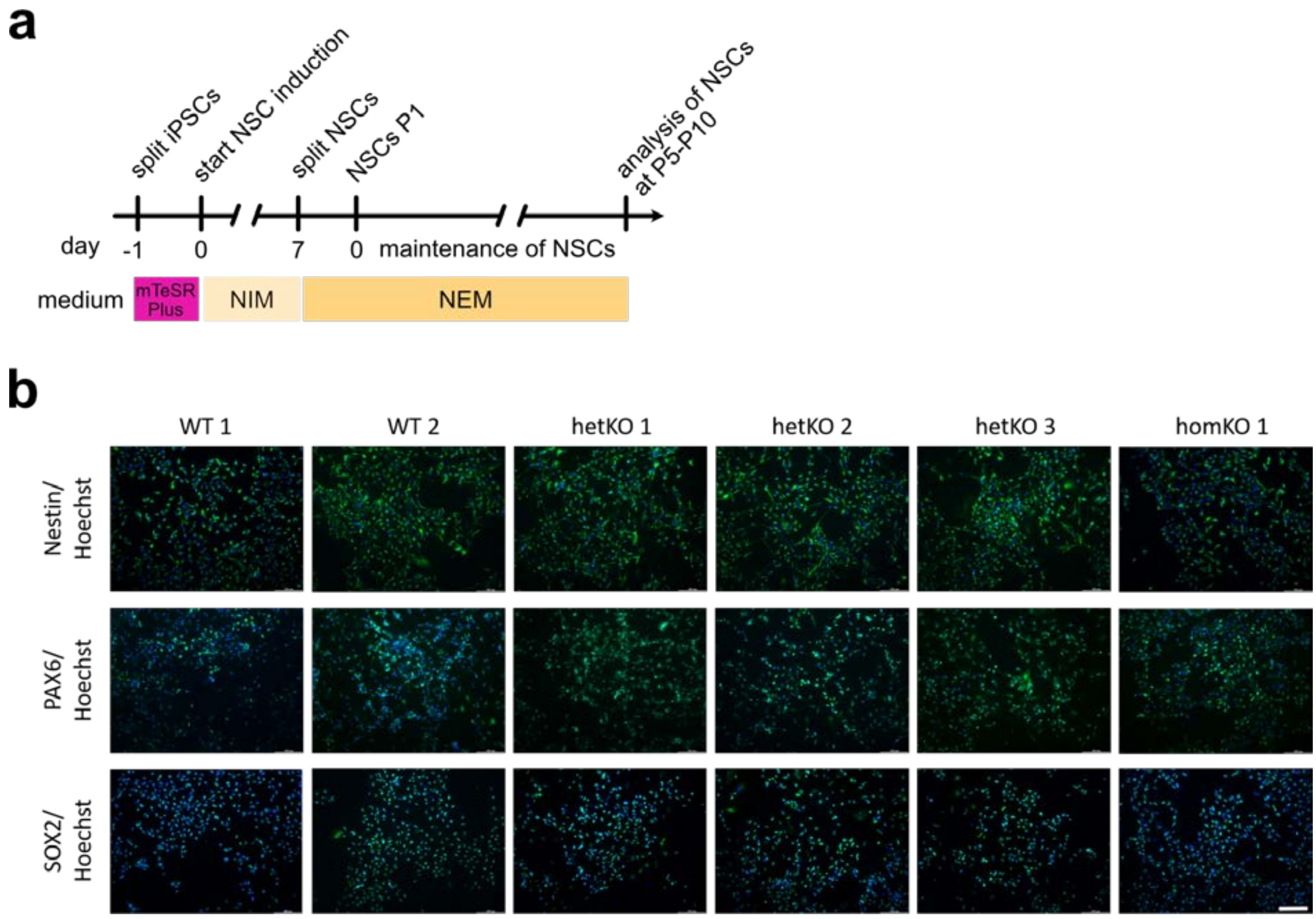

Supplementary Figure 5. Characterization of induced NSCs. a Scheme of NSC induction from iPSCs according to Yan et al. (PMID: 24113065). NIM: neural induction medium, NEM: neural expansion medium. **b** Immunofluorescence images of NSC markers in green (Nestin, PAX6, SOX2) and nucleus staining in blue (Hoechst). All cell lines express all three NSC marker. Scale bar = 100 µm.

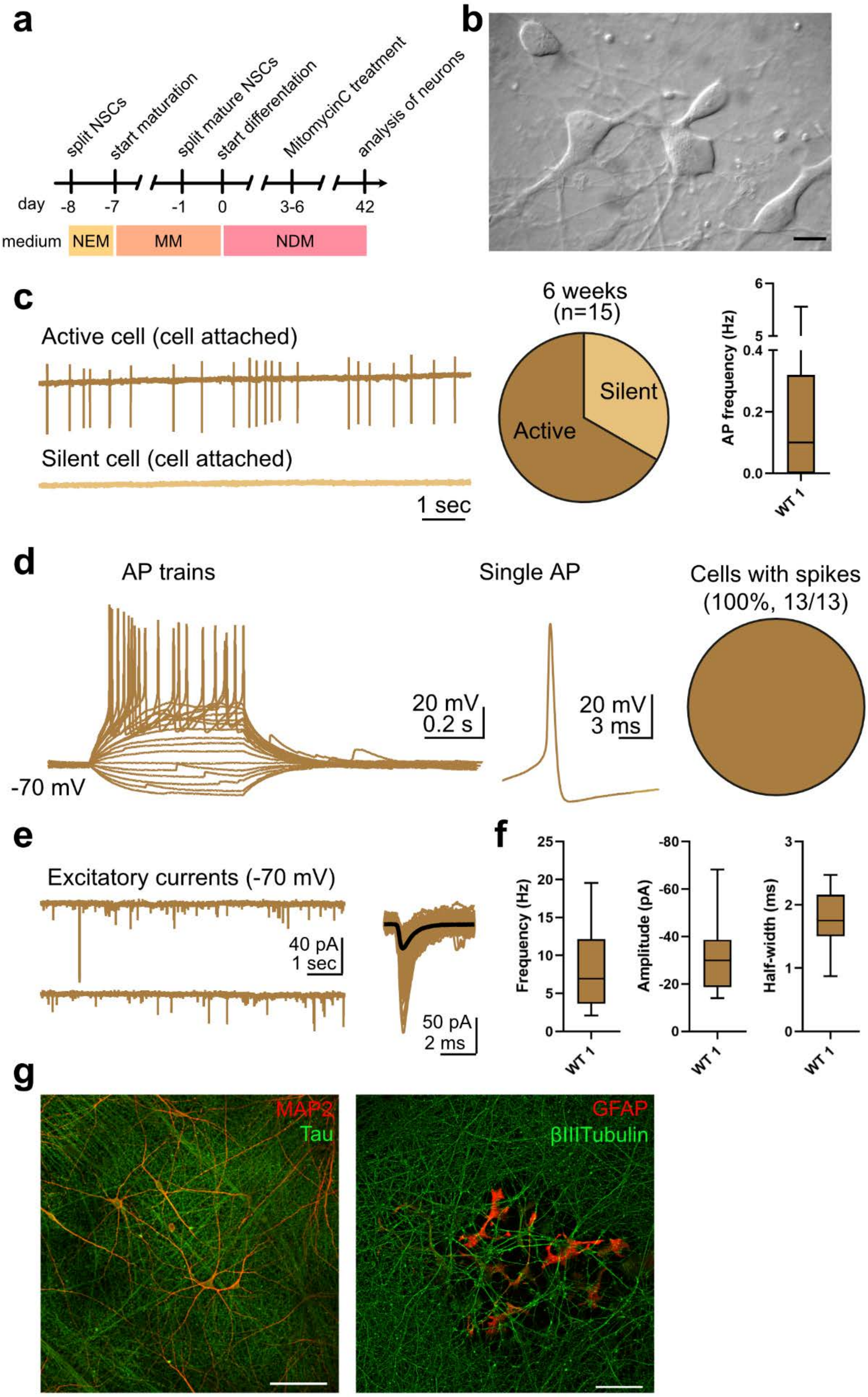

Supplementary Figure 6. Generation and basic characterization of differentiated forebrain neurons. **a** Scheme of differentiation protocol according to Yan et al. (PMID: 24113065). NEM: neural expansion medium, MM: maturation medium, NDM: neuronal differentiation medium. **b** Differential-infrared

contrast image of 6-week-old neurons generated as indicated in (a). Scale bar: 20 µm. **c** Cell-attached recordings of 6-week-old neurons. Under physiological recording conditions, most neurons fired spontaneous spikes at ~0.7 Hz frequencies. **d** Whole-cell current clamp recordings of 6-week-old neurons. All recorded cells fired action potentials upon positive current injections. **e** Whole-cell voltage clamp recordings of 6-week-old neurons, which displayed prominent EPSCs under physiological conditions. **f** Summary graphs for EPSC frequency (left), amplitude (middle), and half-width (right). Summary graphs in c (right) and f represent mean ± SEM. Number of experiments (c:15 cells; f: 16 cells). AP: action potential. **g** Representative immunofluorescence microscopy images with neuronal (MAP2, Tau, and β3-Tubulin) and glial (GFAP) markers after 6 weeks of differentiation. MAP2: microtuble-associated protein 2; GFAP: glial fibrillary acid protein. Scale bar: 100 µm.

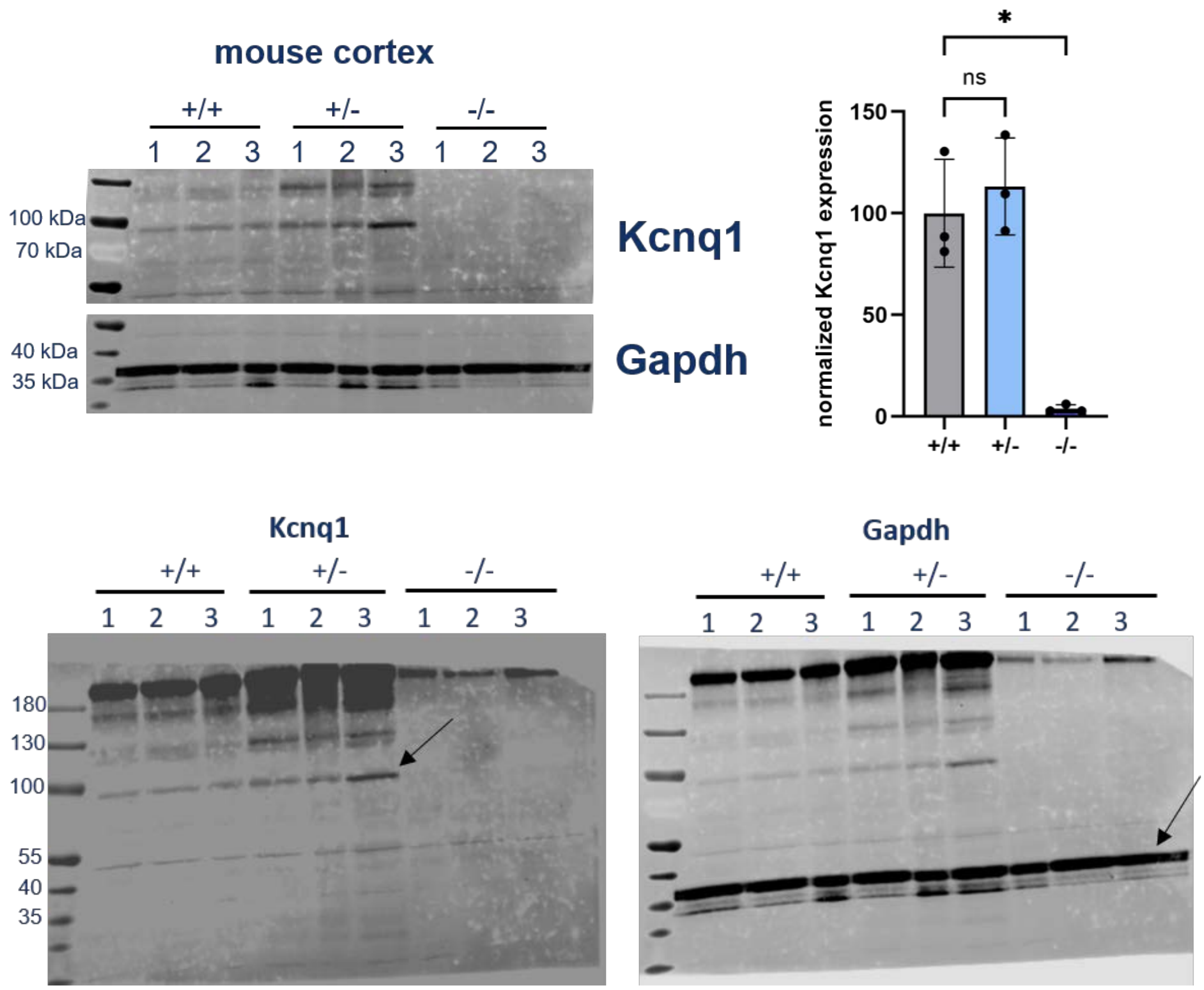

Supplementary Figure 7. Kcnq1 expression in mouse cortex. Western blot analysis using prefrontal cortex tissue from adult (25 weeks old) wildtype, heterozygous and homozygous *Kcnq1* knockout mice (all with a C57BL/6 genetic background). The constitutive *Kcnq1* knockout model was generated by crossing a conditional *Kcnq1* floxed line, in which exons 4-5 are flanked by loxP sites, with a CMV-Cre line, resulting in deletion of the voltage-sensor region and loss of channel function (PMID: 16287990). Knockout animals were included as negative controls to confirm the specificity of the Kcnq1-immunoreactive signal detected at the expected molecular weight of approximately 90 kDa. Upper panel: Representative cropped Western blots and densitometric quantification. Data are shown as mean ± SD (n = 3 animals). Statistical significance was assessed using Welch's one-way ANOVA with Dunnett's T3 post hoc test. Welch's ANOVA: F = 40.55, p = 0.0097; post hoc comparisons: +/+ vs. +/-: p = 0.7851, +/+ vs. -/-: p = 0.0401. +/+, wild type; +/-, heterozygous knockout; -/-, homozygous knockout. Lower panel: Full, uncropped membrane images. Molecular weight standard: PageRuler™ Prestained Protein Ladder (10-180 kDa; Thermo Fisher Scientific). * p <0.05

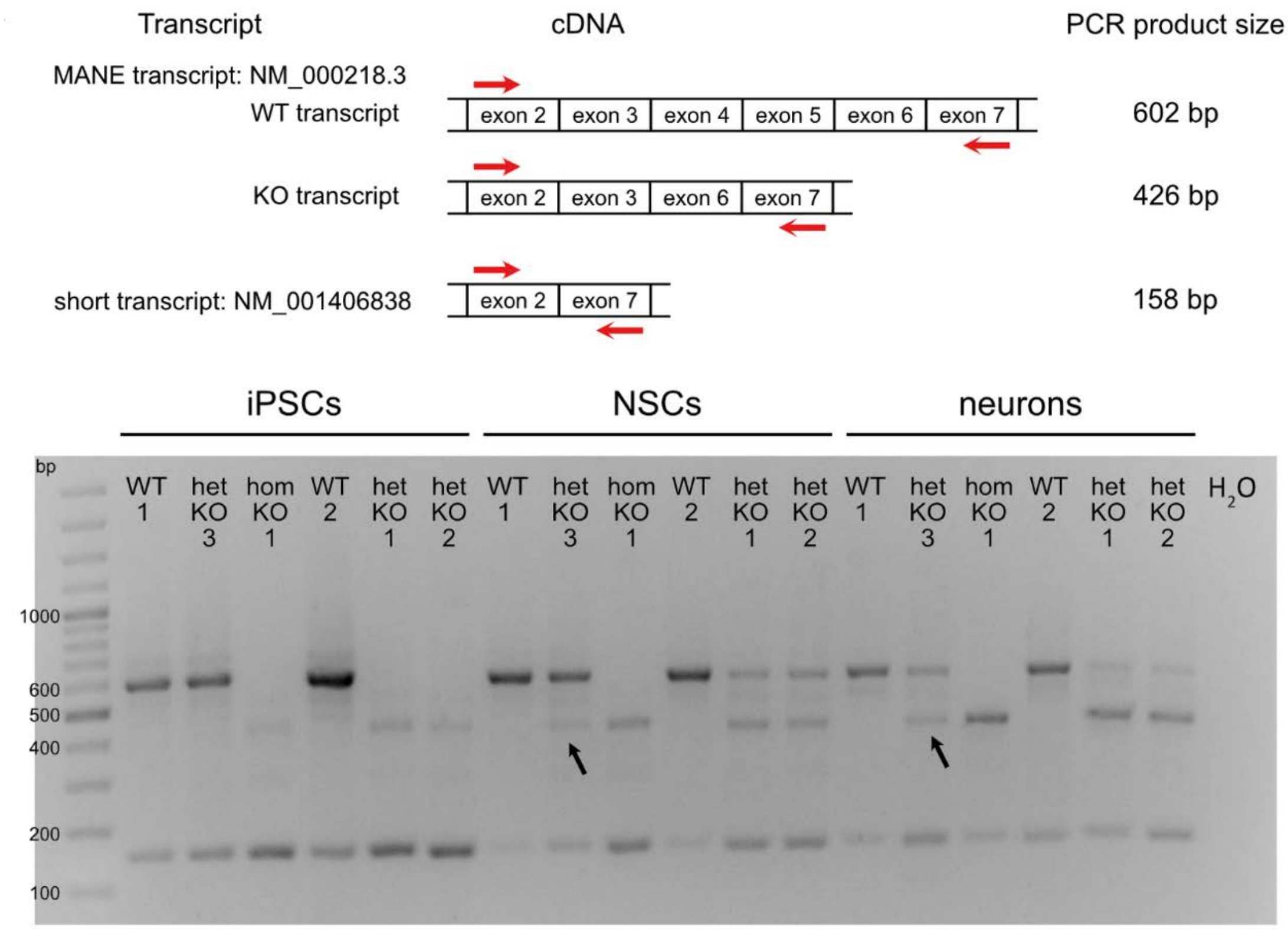

Supplementary Figure 8. Expression analysis of *KCNQ1* isoforms and long non-coding RNA *KCNQ1OT1*. Different *KCNQ1* isoforms have been annotated in the UCSC Human Genome Browser (hg 38) with the MANE (Matched Annotation from the NCBI and EMBL-EBI) transcript, containing exons 2 to 7, and a more recently published short transcript lacking exons 3 to 6 and 8. To date, this isoform has only been reported in RNA sequencing data, and its translation into a functional protein remains unconfirmed. If translated, the resulting protein would lack transmembrane segments 2 to 4 (part of the voltage-sensing domain) and segment 5 (part of the pore domain), making it unlikely to form a functional potassium channel. To elucidate imprinting differences between cell types, we performed PCR on cDNA (RT-PCR) to identify transcription of WT and KO alleles in iPSCs, NSCs, and neurons for the different genotypes. Top: Illustration of the different *KCNQ1* transcripts and the selected primers targeting exon 2 (forward) and exon 7 (reverse) which result in different product sizes based on the number of exons included in the transcript. Bottom: PCR results showing expression of the MANE-WT (band size 602 bp) and -KO transcript (band size 426 bp), and of the short transcript (band size of 158 bp). In iPSCs, WT 1, WT 2, and hetKO 3 (carrying the deletion on the inactive allele) show the WT band. HomKO 1, hetKO 1, and hetKO 2 (both hetKO carrying the deletion on the active allele) show the KO band. In NSCs, both WT lines show the WT band whereas all three heterozygous KO lines show both bands, WT and KO. The homKO 1 NSCs show expression of only the MANE-KO transcript. In neurons, the WT cell lines show the WT band and all heterozygous KO lines show WT and KO expression. The homKO 1 neurons show the KO band of the MANE transcript. All cell lines and cell types express the short *KCNQ1* transcript NM_001406838.1 lacking exon 3-6 (band size: 158 bp). Even though hetKO 3 carries the KO on the inactive allele, imprinting patterns change and both alleles become transcribed in NSCs and neurons (indicted by arrows).

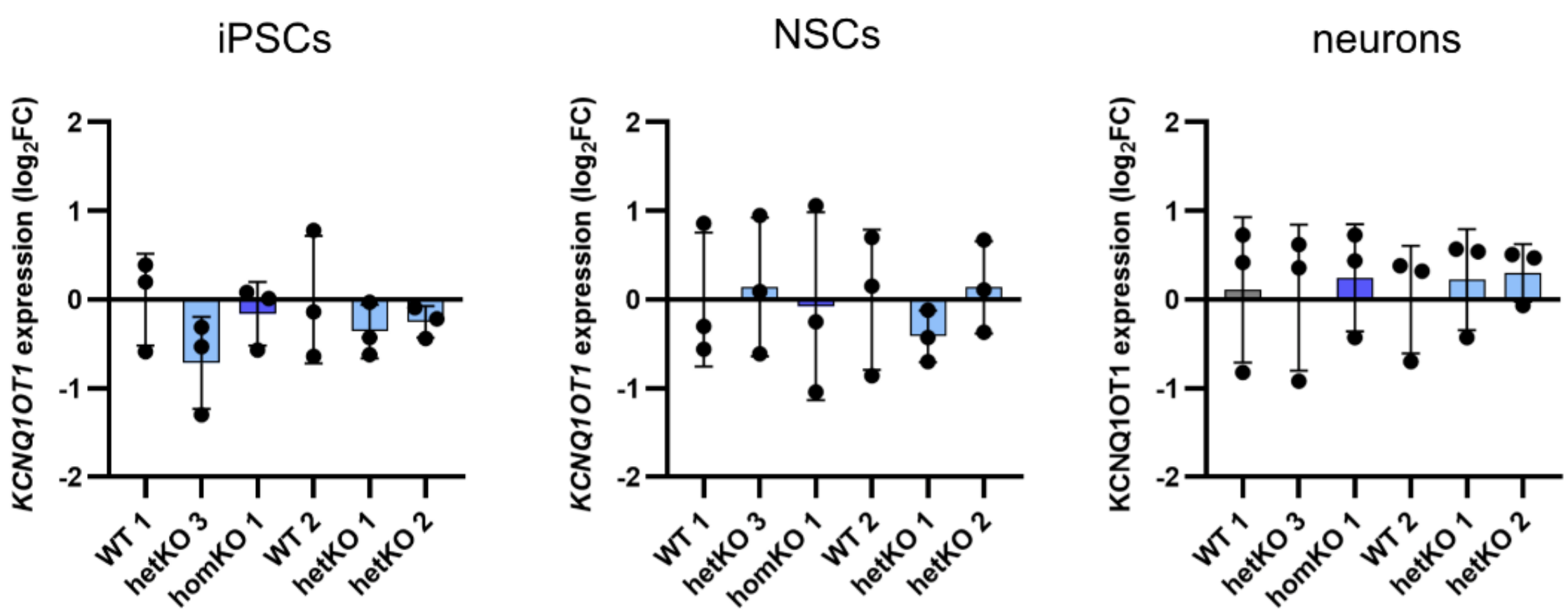

Supplementary Figure 9. Expression analysis of the long non-coding RNA *KCNQ1OT1*. Relative expression of *KCNQ1OT1* in iPSCs, NSCs, and neurons. Expression levels are shown as log2 fold change (log2FC), with knockout (KO) samples normalized to their respective wild-type (WT) controls. Statistical analysis was performed using Welch's one-way ANOVA. No statistically significant differences were observed between groups (all p >0.05). n=3.

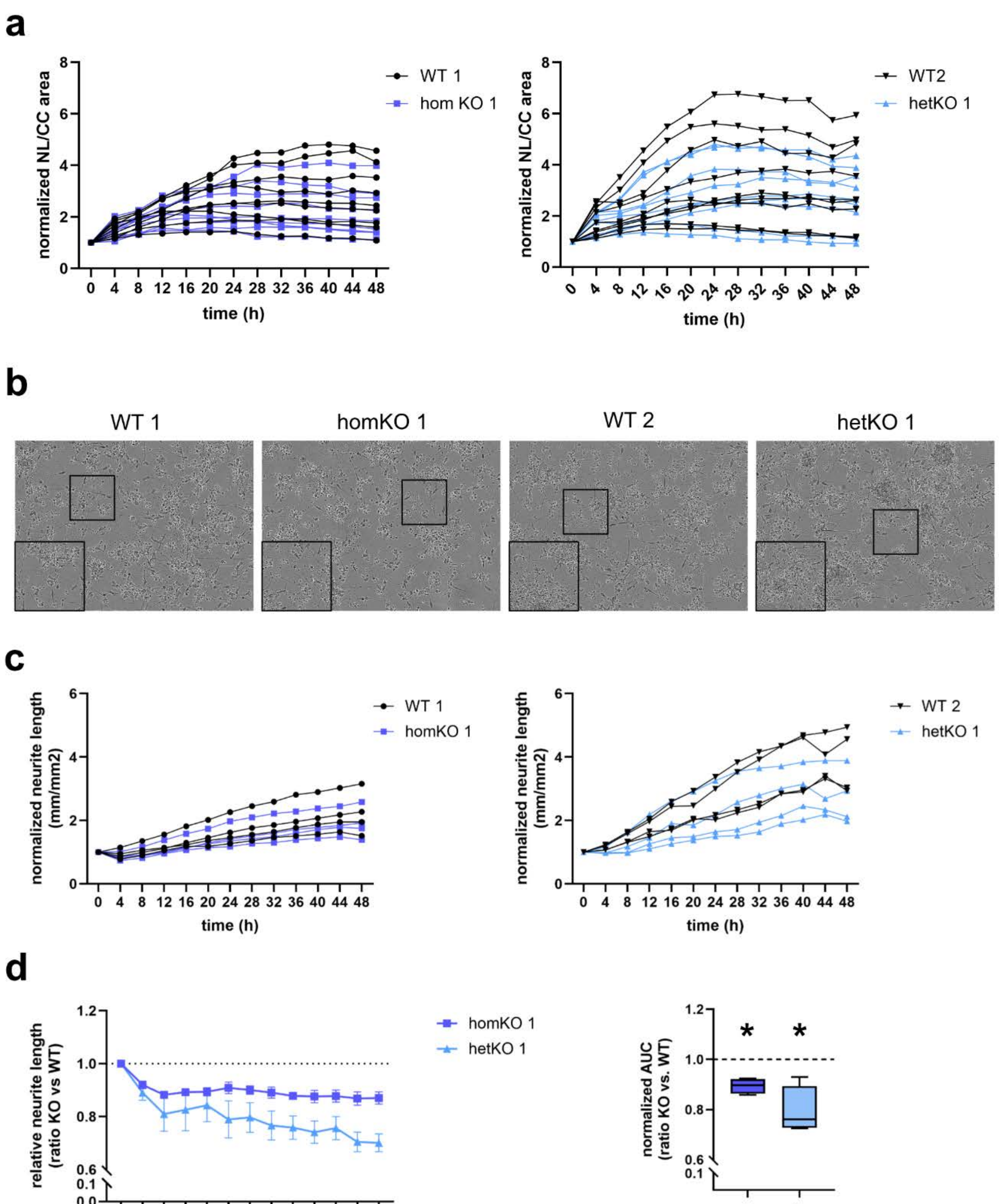

Supplementary Figure 10. Neurite outgrowth measurement during early neuronal differentiation.
a Neurite outgrowth was monitored using the Incucyte system during the first 48 h of neuronal differentiation. The graphs show the neurite length per cell body cluster area (NL/CC) normalized to the first time point, comparing WT 1 with homKO 1 and WT 2 with hetKO 2. Data represent n = 9 biological replicates. NL/CC area was analyzed using a linear mixed-effects model with genotype and time as fixed effects, and experiment as a random effect. While neurite outgrowth increased significantly over time in both datasets ($p < 0.001$), no differences in growth dynamics were observed between WT 1 and homKO 1 cells ($p = 0.258$) or between WT 2 and hetKO 1 cells ($p = 0.458$), as indicated by non-significant genotype × time interactions. **b** Cells were differentiated with an alternative protocol modified according to Qi et. al. (PMID: 28112759) which is described in detail in

the Supplementary materials and methods. Neurite outgrowth was monitored using the Incucyte system during the first 48 h of neuronal differentiation. Representative pictures of all cell lines at time point 48h. **c** The graphs show the neurite length (mm/mm2) normalized to the first time point, comparing WT 1 with homKO 1 and WT 2 with hetKO 2. Data represent n = 4 biological replicates. Neurite length was analyzed using a linear mixed-effects model with genotype and time as fixed effects, time per well as repeated measure, and experiment as a random effect. No differences in growth dynamics were observed between WT 1 and homKO 1 cells (p =0.491) or between WT 2 and hetKO 1 cells (p =0.878), as indicated by non-significant genotype × time interactions. **d** Relative neurite length was calculated for each KO line and normalized to its respective WT control (set to 1; homKO 1 vs WT 1, hetKO 1 vs WT 2). Data are presented as mean ± SEM (n = 4). The area under the curve (AUC) was calculated from normalized time-course data and analyzed using a one-sample t-test with Bonferroni correction. Adjusted p-values: homKO 1, p =0.0114; hetKO 1, p =0.0446. The dotted line indicates the normalized WT controls.

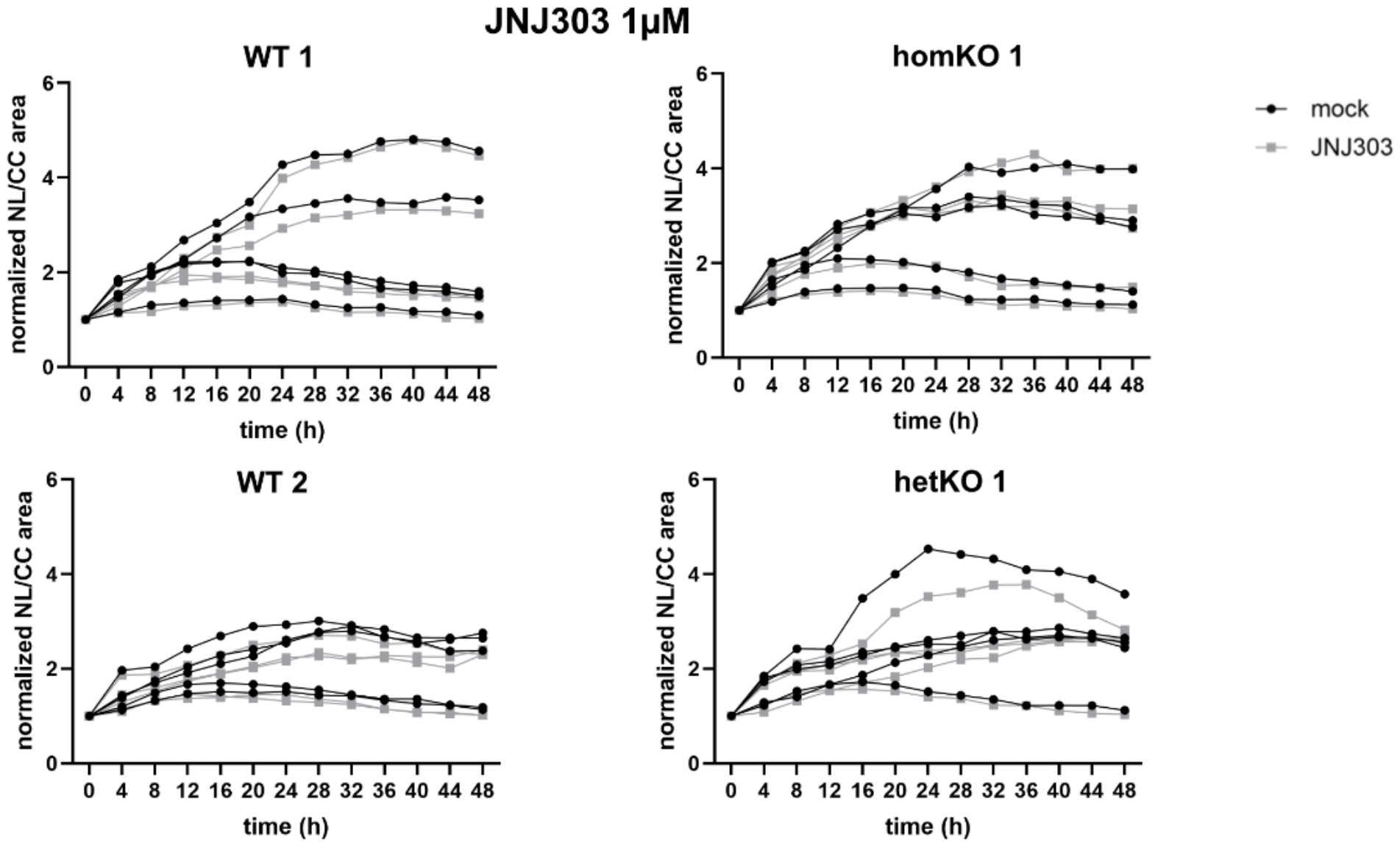

Supplementary Figure 11: Neurite outgrowth JNJ303 treatment. 1 µM, n=5. NL/CC area (normalized to the initial time point) was measured over time in WT 1, WT 2, homKO 1 and hetKO 1 cells under mock and treatment conditions. Linear mixed-effects modelling (neurite length per cell body cluster area ~ genotype × treatment × time, with well and experiment as random effects) revealed a significant effect of time ($p < 0.05$), but no significant effects of genotype, treatment, or their interaction on neurite growth dynamics (all interaction terms $p > 0.1$).

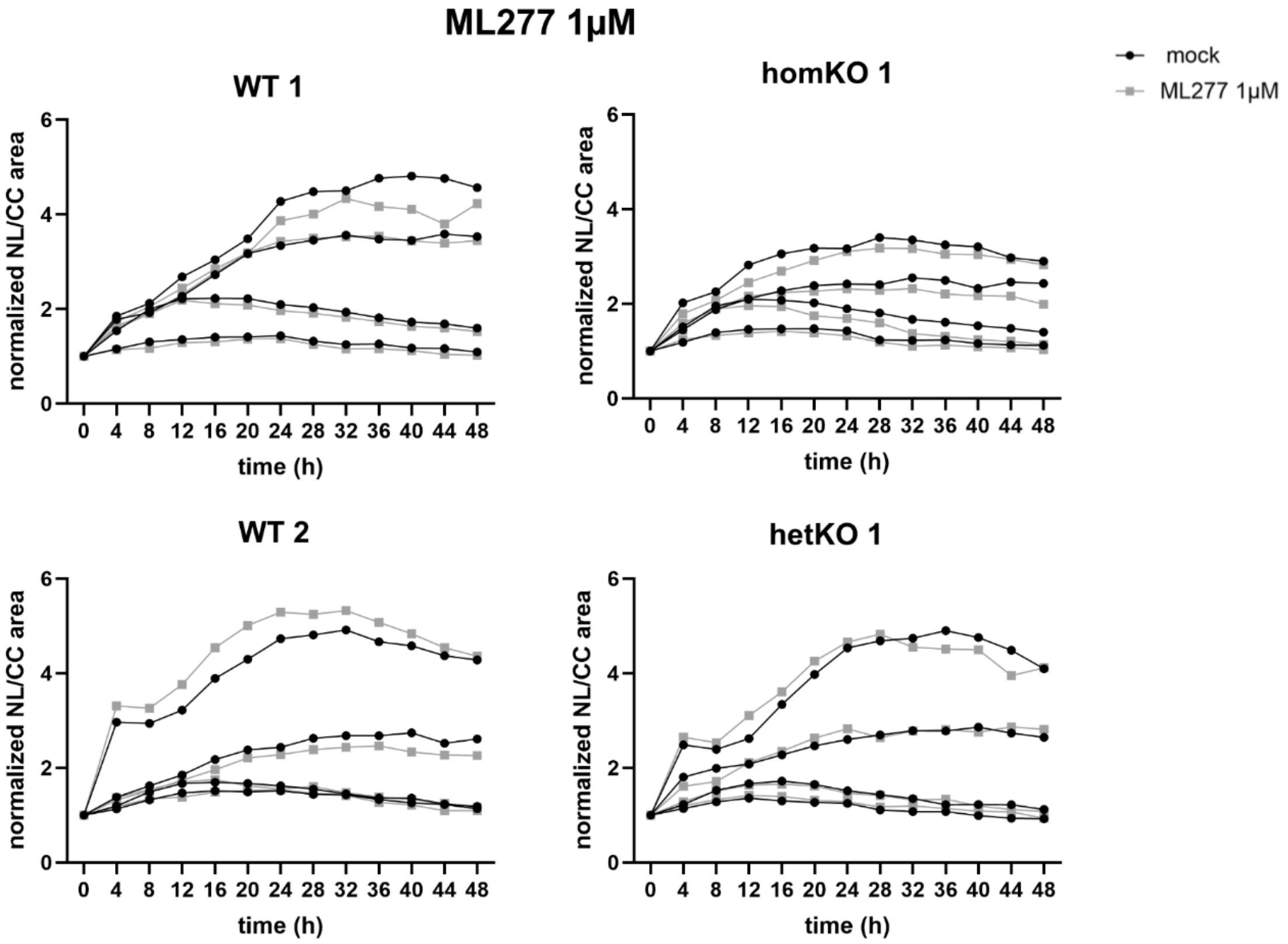

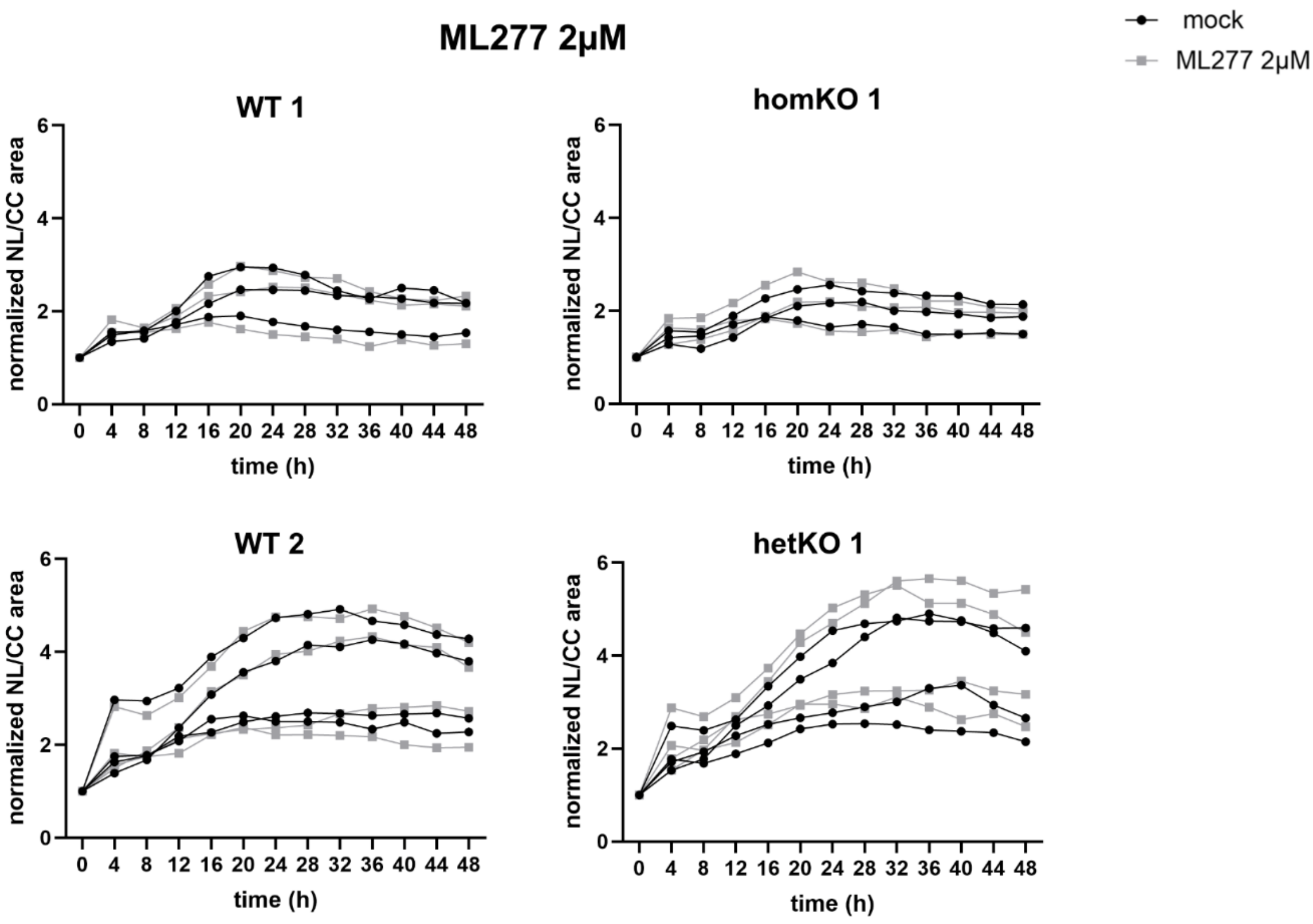

Supplementary Figure 12. ML277 treatment. **a** ML277 (1 µM) n=4. NL/CC area values were normalized to baseline and analyzed using a linear mixed-effects model. Neurite outgrowth increased modestly

over time (p =0.028), but no significant effects of genotype or treatment were observed. In particular, no differences in growth dynamics were detected between WT 1 and homKO 1 or between WT 2 and hetKO 1 cells, and no genotype-dependent response to treatment was identified (all interaction terms $p > 0.3$). **b** ML277 (2 µM**)** WT 1/homKO 1 n=3 experiments, WT 2/hetKO 1 n= 4**.** NL/CC area values were normalized to baseline and analyzed using the linear mixed-effects model. Neurite outgrowth over time was not statistically significant (p >0.05) in either WT 1/homKO 1 or WT 2/hetKO 1 datasets. Consistently, no effects of genotype, treatment, or genotype-dependent treatment responses were detected (all interaction terms p >0.7). The absence of increased NL/CC area over time contrasts with previous experiments and may be attributable to the higher DMSO concentration used to dissolve ML277 at 2 µM, which was also present in mock-treated controls.

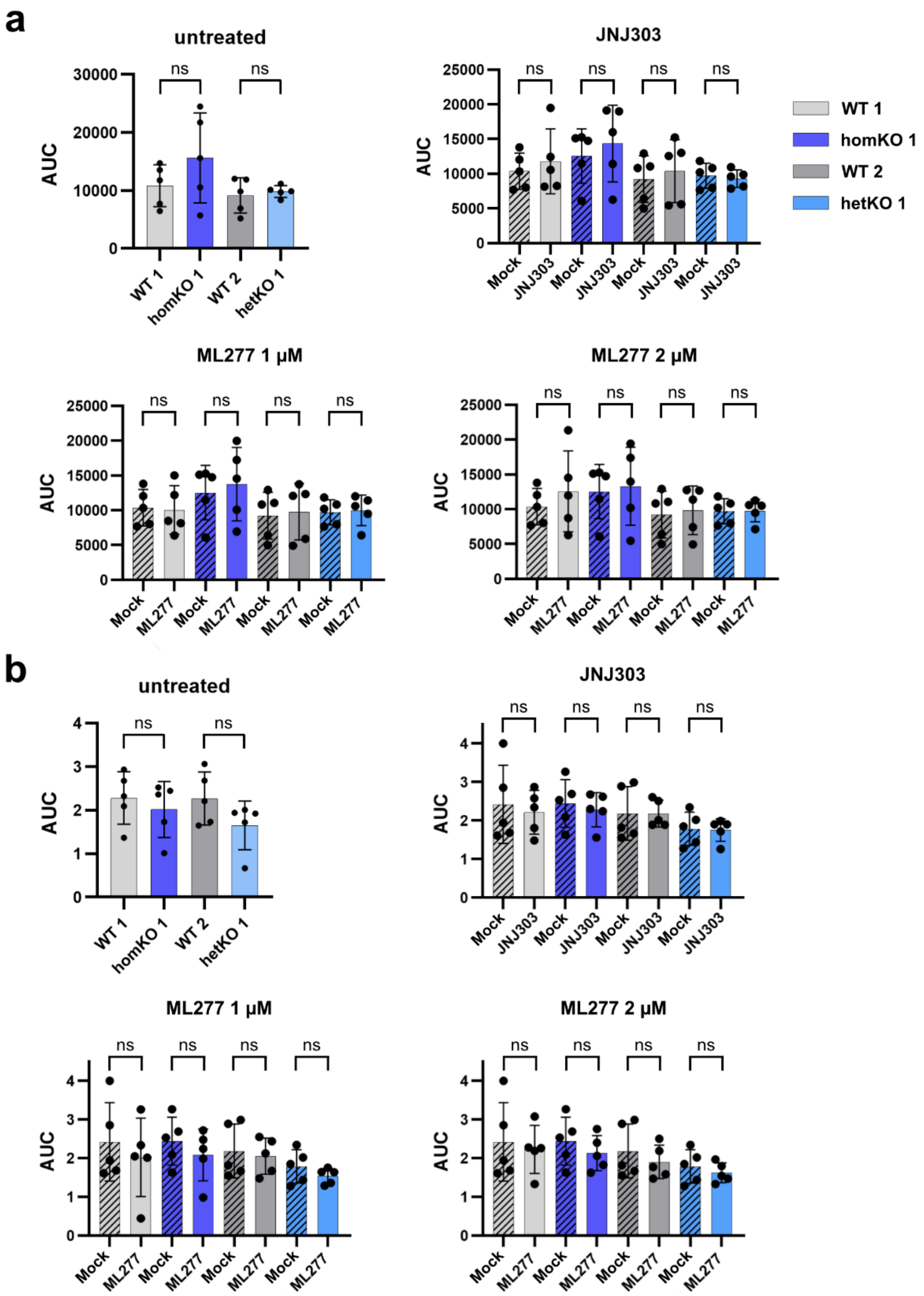

Supplementary Figure 13. Analysis of cell proliferation and apoptosis. Proliferation and apoptosis were either measured untreated or treated with KCNQ1 antagonist JNJ303 (1 µM) or KCNQ1 agonist ML277 (1 µM, 2 µM) with the Incucyte system. The hatched bars represent the mock treated samples. **a** Proliferation analysis and **b** apoptosis analysis revealed no differences. The area under the curve (AUC) of cell numbers and of the apoptosis rate over 48h was calculated. Welch´s one-way ANOVA was used as AUC values showed different variance, mean ± SD n=5. all *p*-values > 0.05.

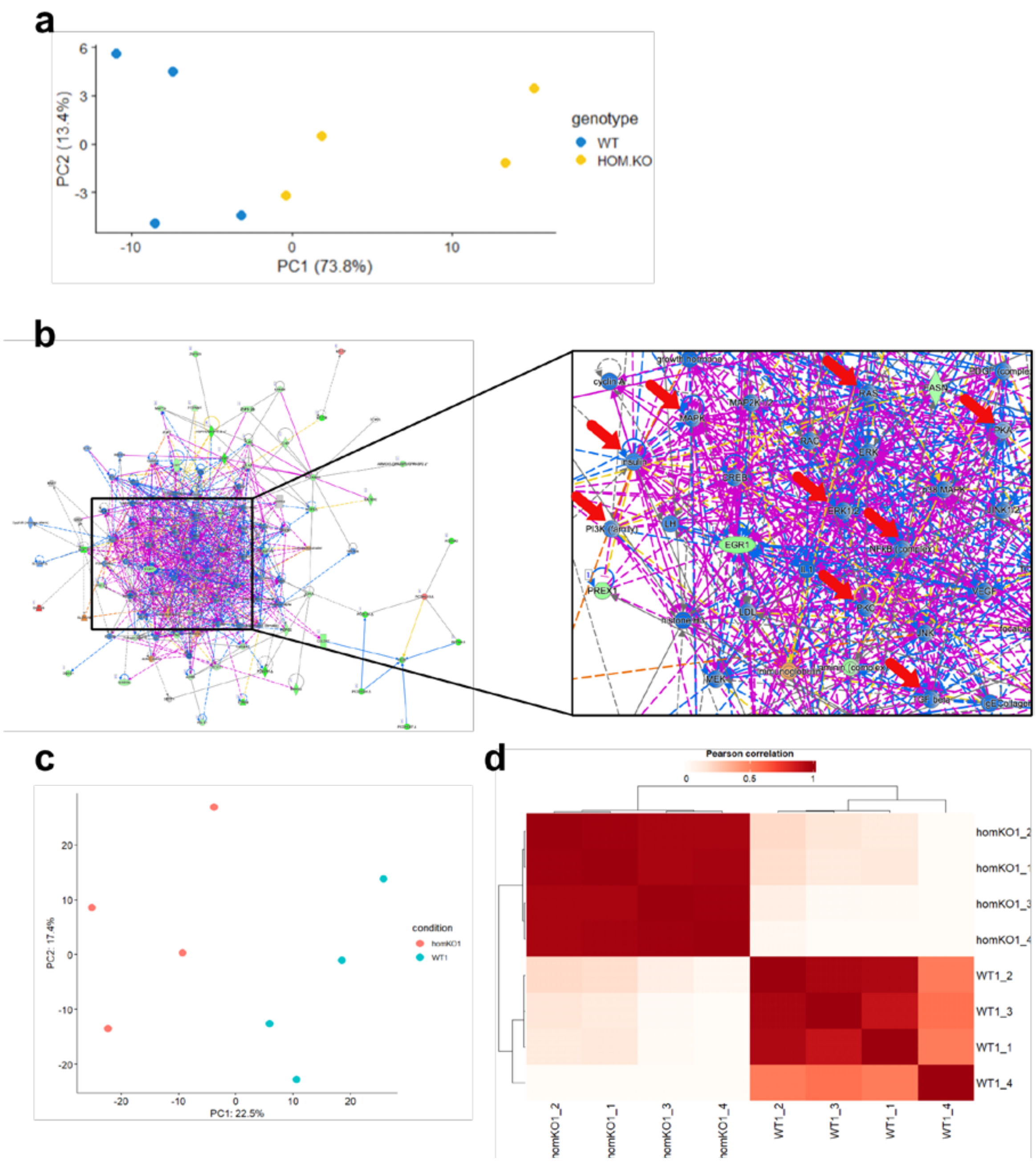

Supplementary Figure 14. Analysis of RNA sequencing data and mass spectrometry data from WT 1 and homKO 1 NSCs. a PCA of RNA sequencing data. PC1 and PC2 account for 73% and 13.4% of the variance in the data, respectively. Samples cluster according to the genotype conditions separating along PC1. Color-coding by condition reveals distinct clustering, suggesting that the genotype is a major driver of variance. **b** Network enrichment analysis of the transcriptomic data in NSCs (IPA). The three most significantly enriched networks for all DEGs were merged. The network enrichment analysis points towards an involvement of insulin signalling (including downstream molecules RAS, MAPK, ERK, PI3K), TGFβ, NFκB, PKC and PKA signalling (indicated by red arrows). Genes: green: downregulated in expression, red: upregulated in expression, blue: predicted to be inhibited, orange: predicted to be activated, grey: differentially expressed but not significantly, white: not present; arrows: purple: overlap between networks, orange: predicted positive effect, blue: predicted negative effect, yellow: inconsistent/unpredicted effect, grey: no predicted effect. **c** PCA of proteomic data from WT 1 and homKO 1 NSCs. PC1 and PC2 explain 22.5% and 17.4% of the data variance. According to the separation

of genotypes in PC1, the genotype seems to be the main cause of variance between groups. **d** Pearson correlation analysis revealed clustering of the NSCs according to the different genotypes.

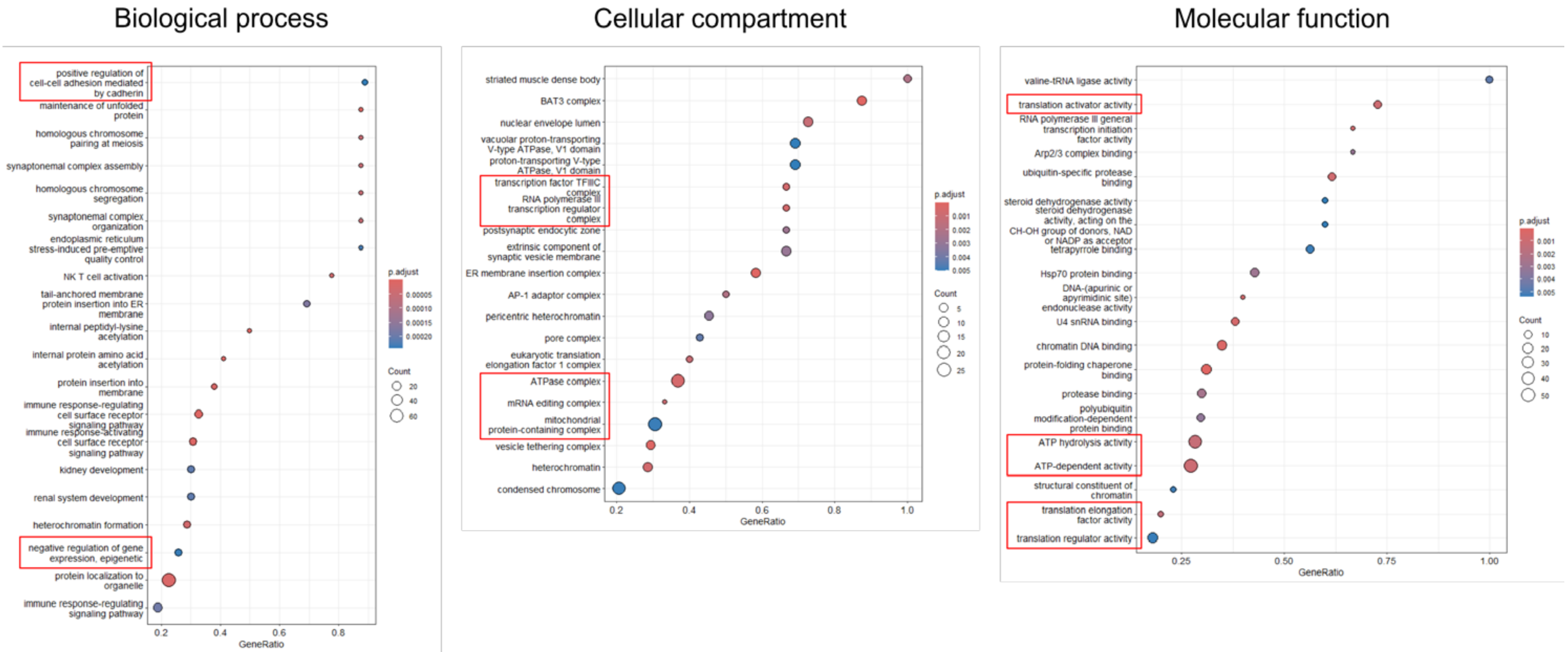

Supplementary Figure 15. GSEA comparing homKO 1 to WT 1 in NSCs. All nominal significant proteins with their log2fold changes were considered for the analysis. The top 20 affected biological processes, cellular compartments, and molecular functions are shown. Most interesting findings were labelled in red boxes and are summarized in the main Table 1.

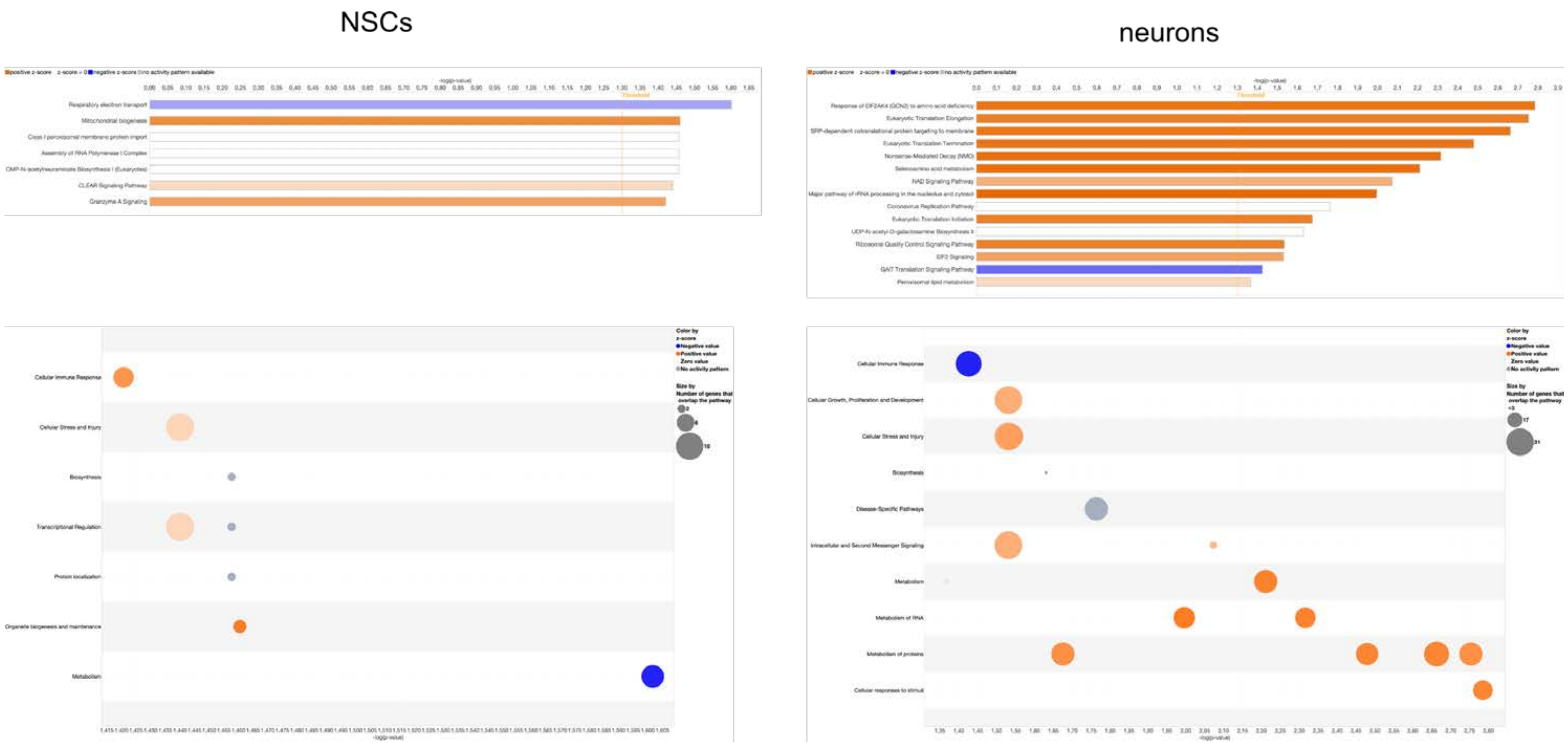

Supplementary Figure 16. IPA canonical pathway enrichment analyses of proteomic data in NSCs and neurons comparing homKO 1 to WT 1. All nominally significant proteins were considered for the analysis together with their log2fold changes. The bar graph at the top shows the significantly enriched canonical pathways (i.e., with nominally significant *p*-value), with orange (positive z-scores) and blue (negative z-cores) indicating predicted activation and inhibition of the pathway, respectively. The bubble blot at the bottom provides a holistic view of all identified cellular mechanisms.

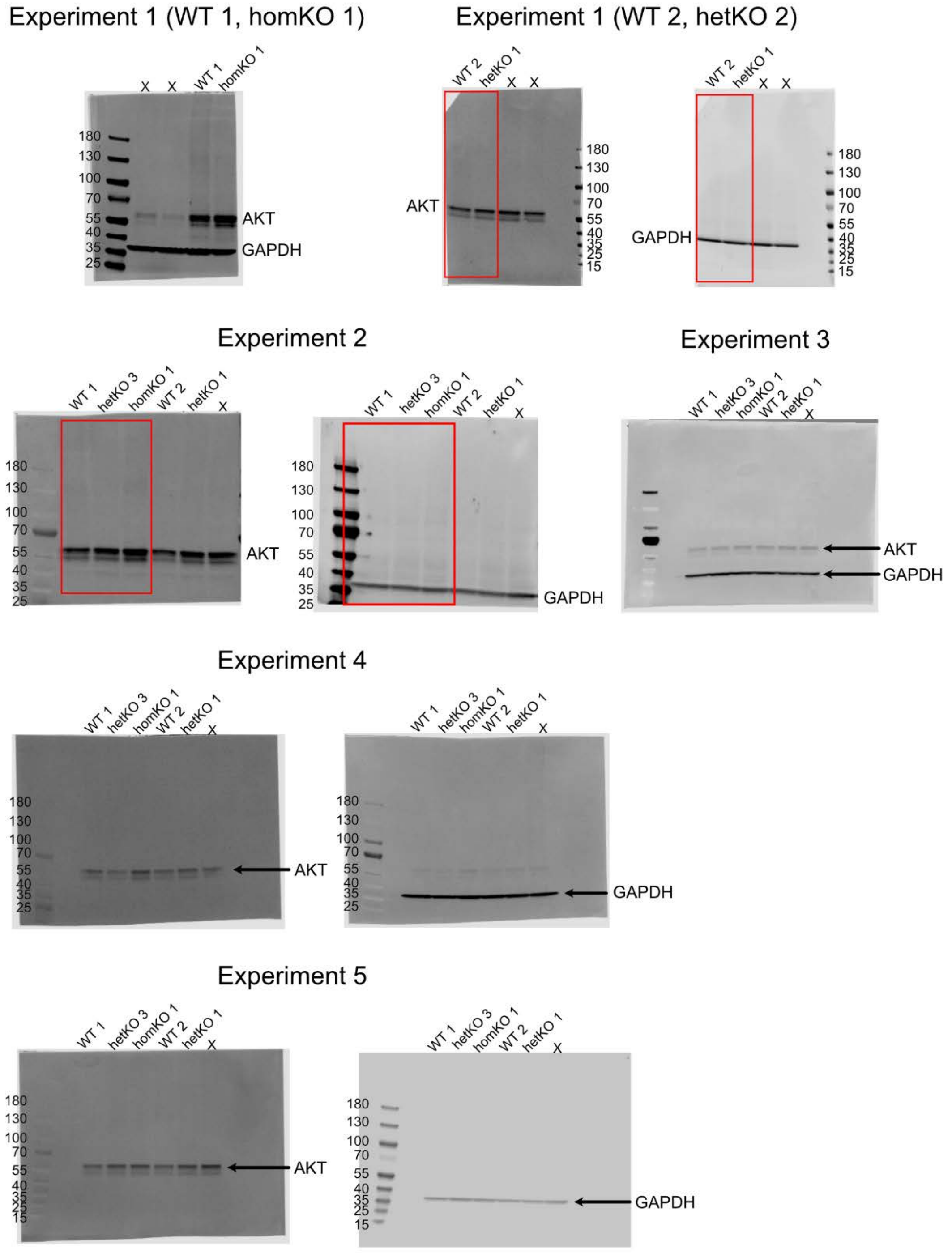

Supplementary Figure 17. Uncropped Western blot pictures linked to Figure 3 f. Quantification of AKT and GAPDH. Molecular weight marker: page ruler prestained protein ladder (10-180 kDa, Thermo scientific). X – not used cell lines. Red boxes – cropped data in main figure.

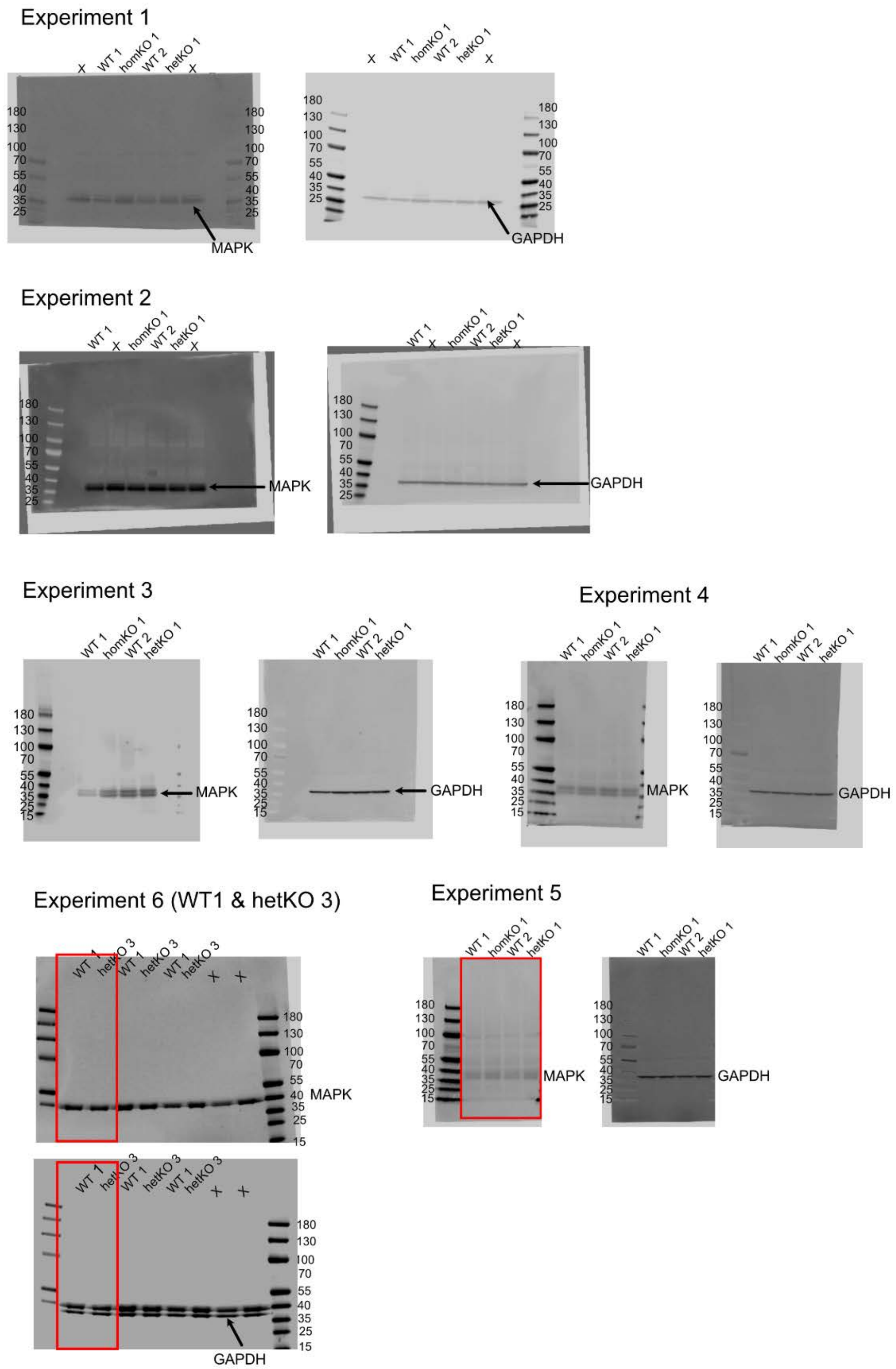

Supplementary Figure 18a. **Uncropped Western blot pictures linked to Figure 3 g.** Quantification of MAPK and GAPDH. Molecular weight marker: page ruler prestained protein ladder (10-180 kDa, Thermo scientific). X – not used cell lines. Red boxes – cropped data in main figure.

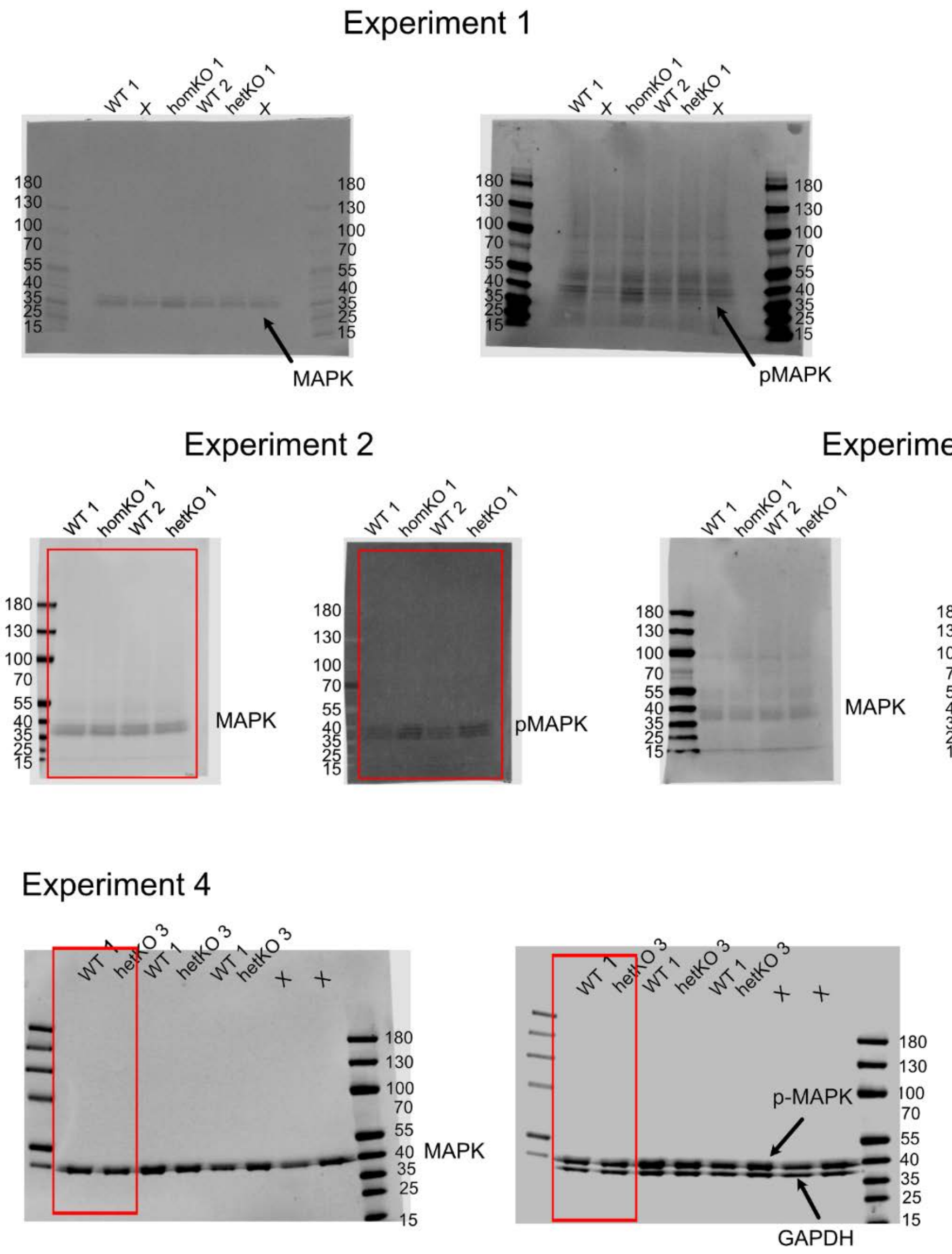

Supplementary Figure 18b. **Uncropped Western blot pictures linked to Figure 3 h.** Quantification of p-MAPK and MAPK. Molecular weight marker: page ruler prestained protein ladder (10-180 kDa, Thermo scientific). X – not used cell lines. Red boxes – cropped data in main figure.

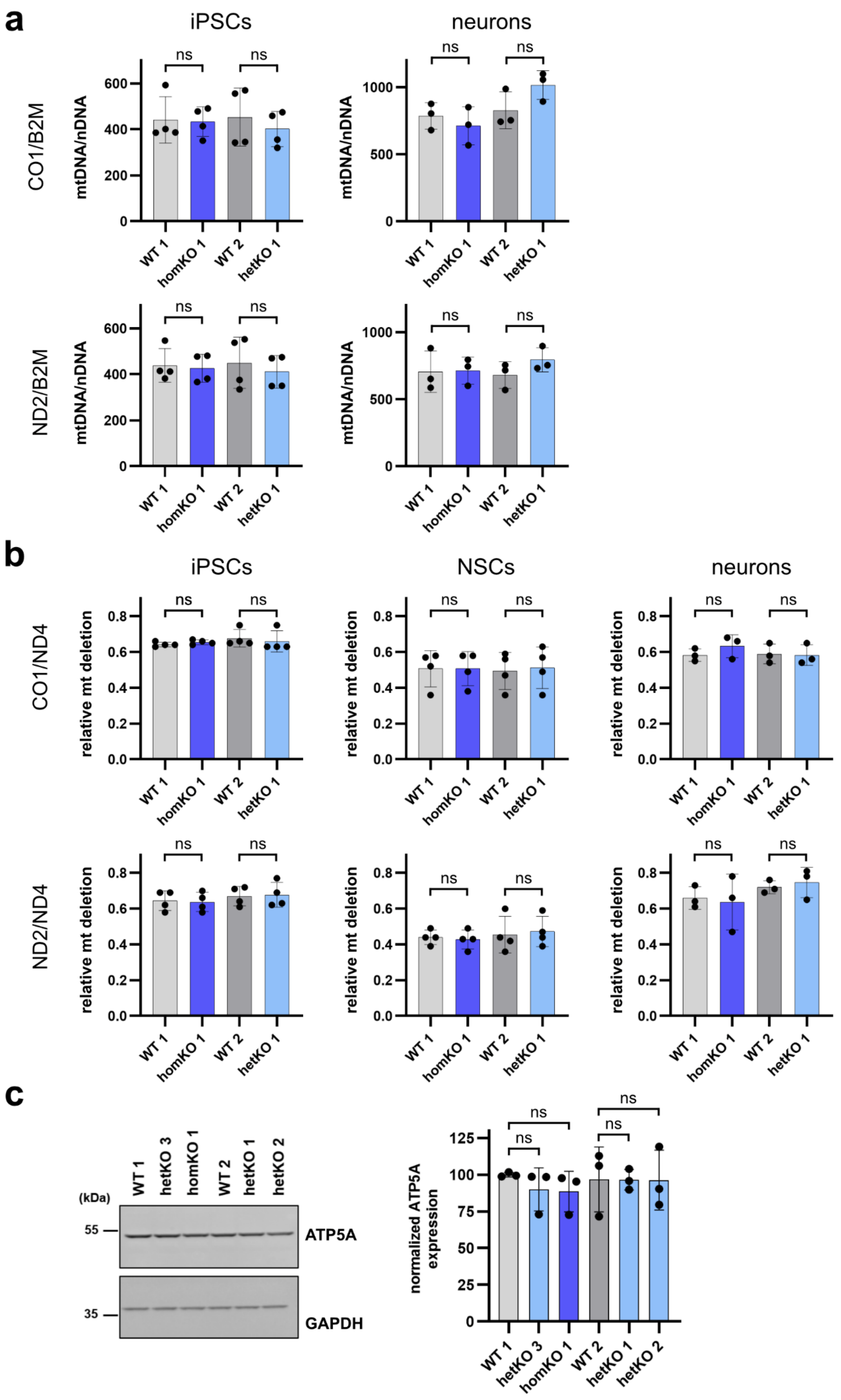
a
iPSCs
neurons
CO1/B2M
ND2/B2M
mtDNA/nDNA
ns
WT 1
homKO 1
WT 2
hetKO 1
b
iPSCs
NSCs
neurons
CO1/ND4
ND2/ND4
relative mt deletion
c
(kDa)
55
35
WT 1
hetKO 3
homKO 1
WT 2
hetKO 1
hetKO 2
ATP5A
GAPDH
normalized ATP5A expression

Supplementary Figure 19: Analysis of mitochondria in iPSCs, NSCs, and neurons. a Quantification of mitochondrial DNA (mtDNA, genes: *CO1,ND2*) against nuclear DNA (nDNA, gene: *B2M*) in iPSCs and neurons. No difference was observed in any cell line or cell type. iPSCs: n=4, neurons: n=3. **b** Analysis of mitochondrial DNA deletion in iPSCs, NSCs, and neurons by quantification of the amount of mitochondrial genes *CO1*, *ND2* against *ND4*, showed no difference for both KO lines in all three cell types. iPSCs: n=4, neurons: n=3 biological replicates. **c** Western blot analysis of ATP5A expression in neurons (left: representative membrane image, right: quantification). ATP5A shows similar expression levels for all cell lines. n=3 biological replicates. **a, b** One-way ANOVA, all *p* >0.05, **c** Welch´s ANOVA, all *p* >0.05, mean ± SD. *ND2*: *NADH dehydrogenase 2*; *CO1*: *cytochrome C oxidase 1*; *ND4*: *NADH dehydrogenase 4*; *β2M*: *beta-2 microglobulin*; ATP5A: ATP synthase F1 subunit alpha.

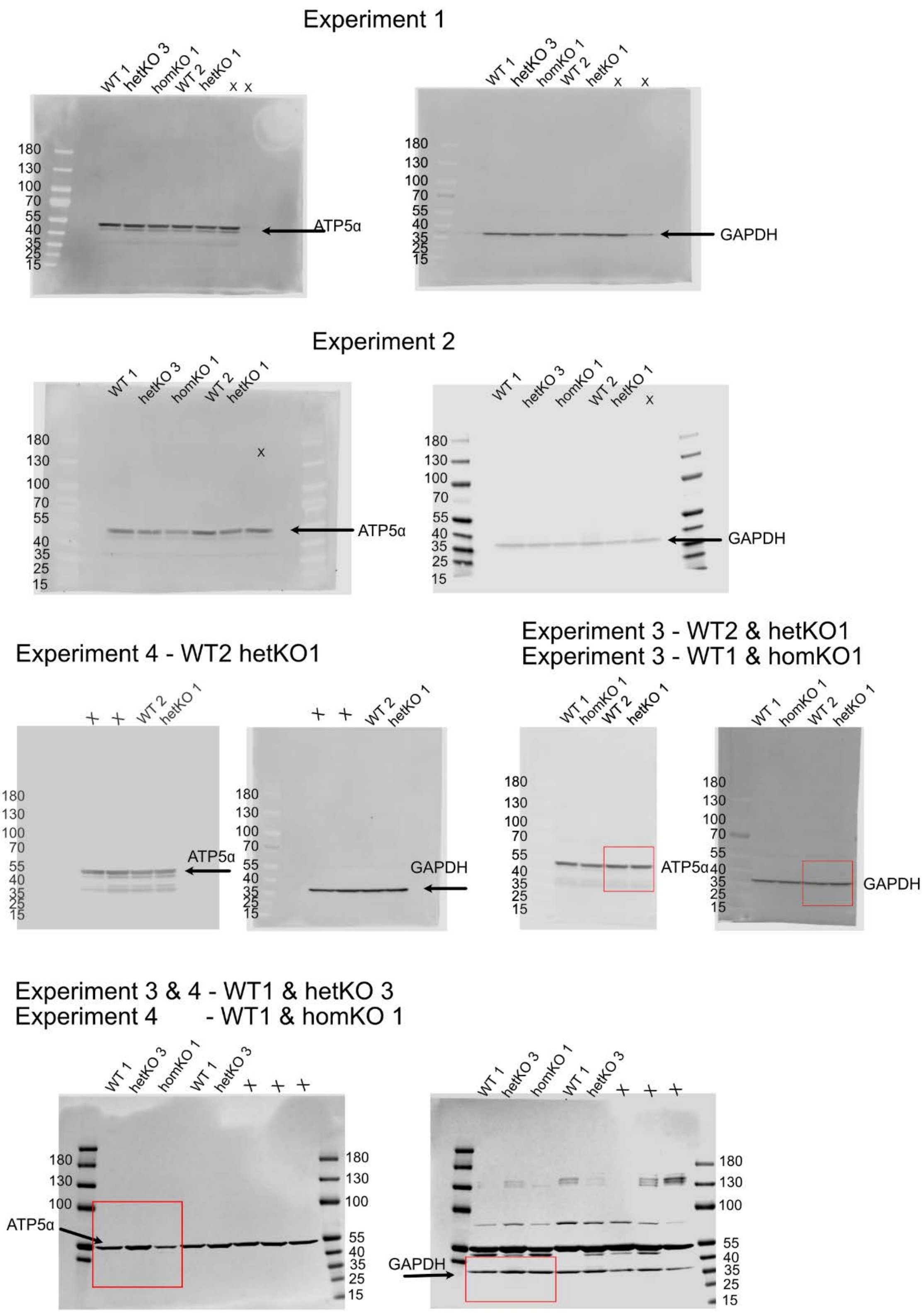

Supplementary Figure 20. **Uncropped Western blot pictures linked to Figure 4b.** Quantification of ATP5A and GAPDH. Molecular weight marker: page ruler prestained protein ladder (10-180 kDa, Thermo scientific). X – not used cell lines. Red boxes – cropped data in main figure.

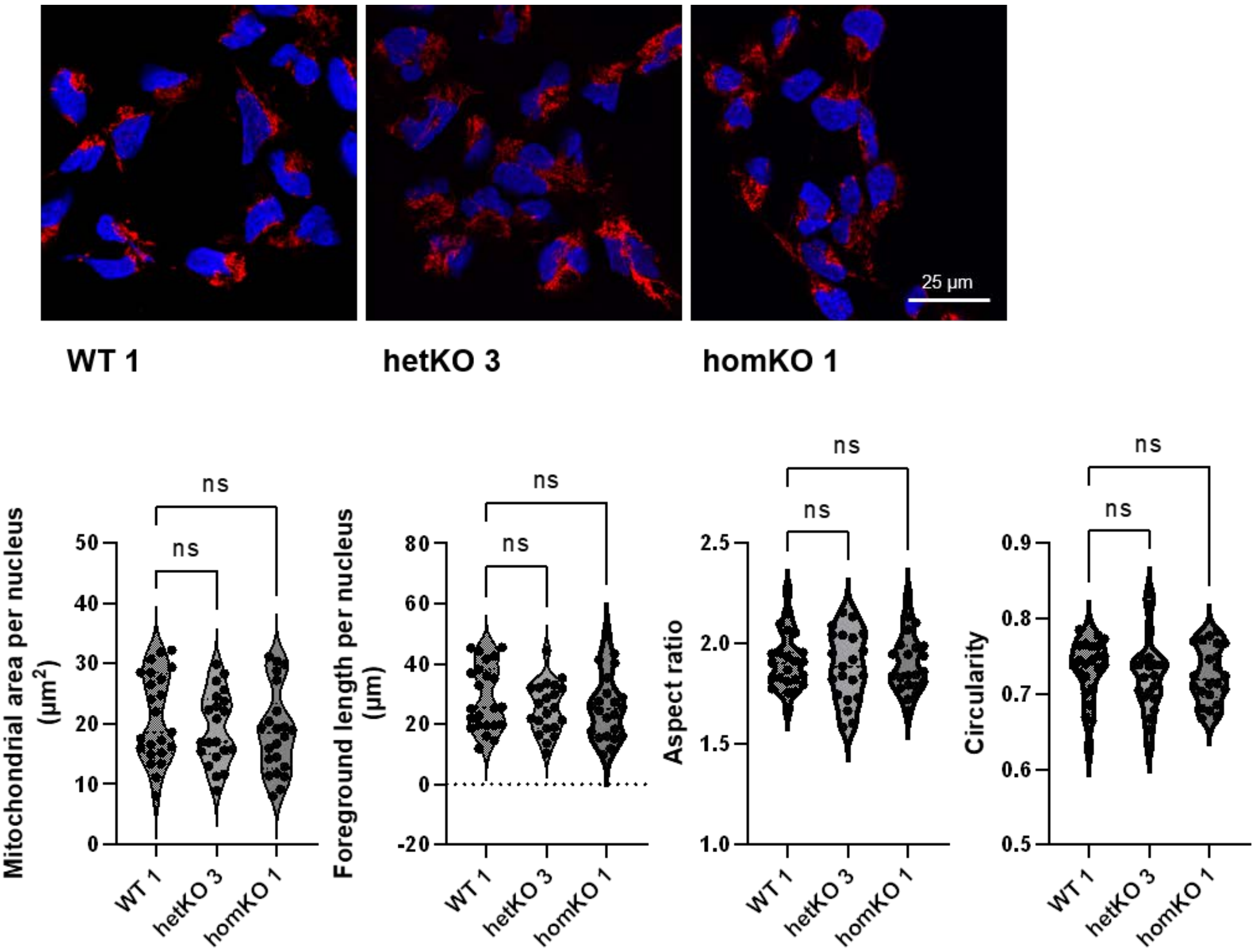

Supplementary Figure 21. Mitochondrial morphology analysis in WT 1, hetKO 3, and homKO 1 NSCs. Representative fluorescence images of WT 1, hetKO 3, and homKO 1 NSCs stained with MitoTracker Red to visualize the mitochondrial network. Morphological parameters were quantified from MitoTracker Red images using FIJI/ImageJ. Quantification of mitochondrial area per nucleus, foreground length per nucleus, aspect ratio and circularity. Mitochondrial area per nucleus reflects the total mitochondrial content per cell, whereas foreground length per nucleus represents the extent of the mitochondrial network. Aspect ratio and circularity were used to assess mitochondrial shape and elongation. Data were analyzed using linear mixed-effects models with genotype as a fixed effect and biological replicate as a random effect. Pairwise comparisons were performed using Tukey-adjusted estimated marginal means. No significant differences were detected between genotypes for any of the quantified mitochondrial morphology parameters (all p >0.35). Each data point in the violin plot represents one cell. n = 3 experiments.

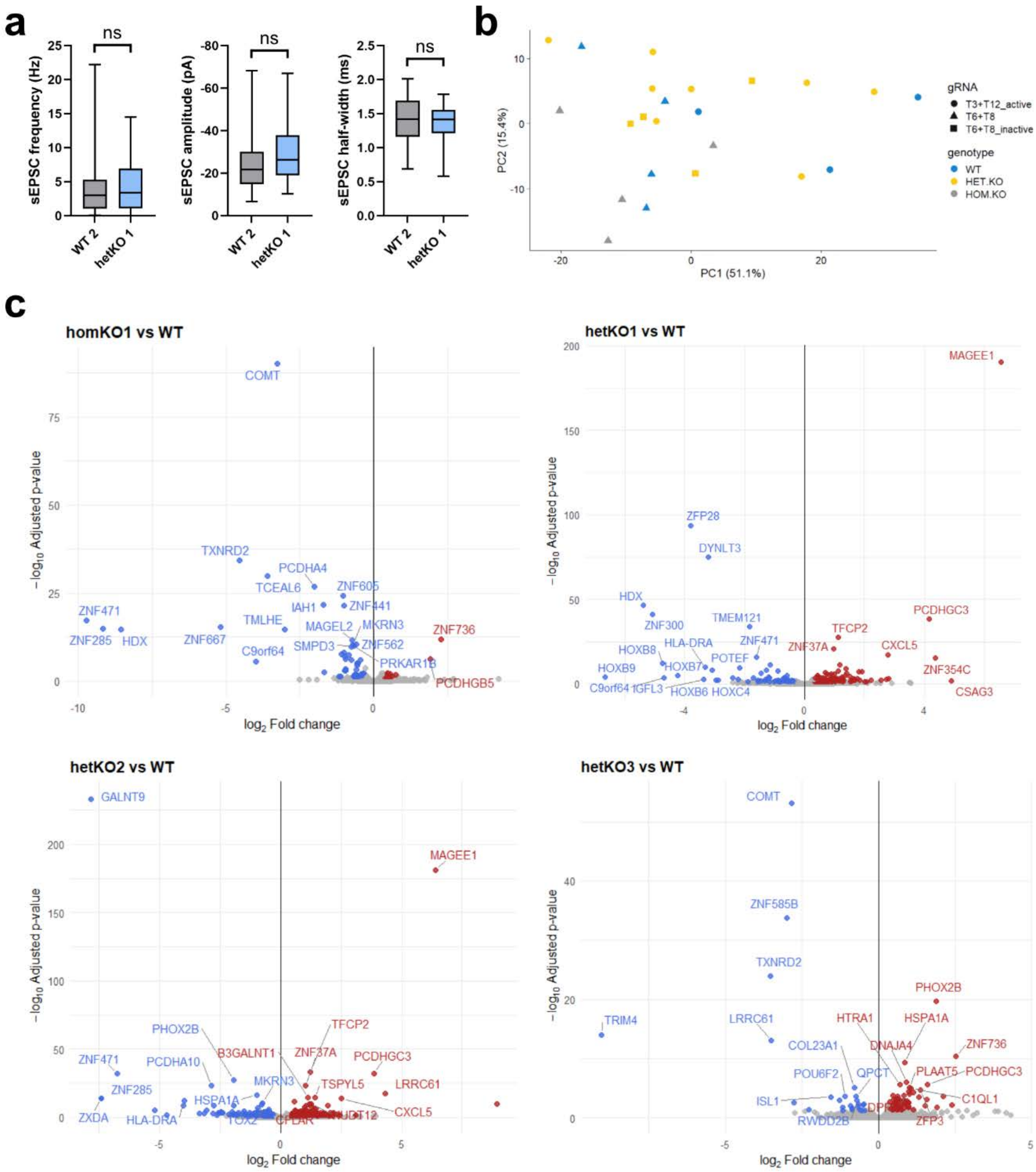

Supplementary Figure 22. Electrophysiology measurements of hetKO 1 and WT 2, and transcriptomic analysis in *KCNQ1*-KO neurons. a Measurements of sEPSC frequency, amplitude, and half-width of hetKO 1 and WT 2 does not show any differences between WT and KO (all p >0.05). Unpaired two-tailed Student's t-test, mean ± SEM. **b** PCA of RNA sequencing data from WT 1, WT 2, hetKO 1, hetKO 2, hetKO 3, and homKO 1 neurons did not reveal distinct clustering among the different cell lines. PC1 and PC2 explain 51.1 % and 15.4% of the variance, respectively, but this variation does not align with the experimental groups. Samples from different genotypes are interspersed across the PCA plot, with only slight separation for homKO 1. The overlapping distribution suggests either minimal transcriptional differences across conditions or that relevant variation lies outside the first two components. **c** Volcano plots showing differentially expressed protein coding genes in neurons. Genes with FDR < 0.05 and log2fold change >1.3 are shown (red: significantly upregulated genes, blue: significantly downregulated genes).

a

Comparison	Total	Up	Down
homKO 1 vs WT	64	14	50
hetKO 1 vs WT	189	125	64
hetKO 2 vs WT	377	226	151
hetKO 3 vs WT	100	74	26

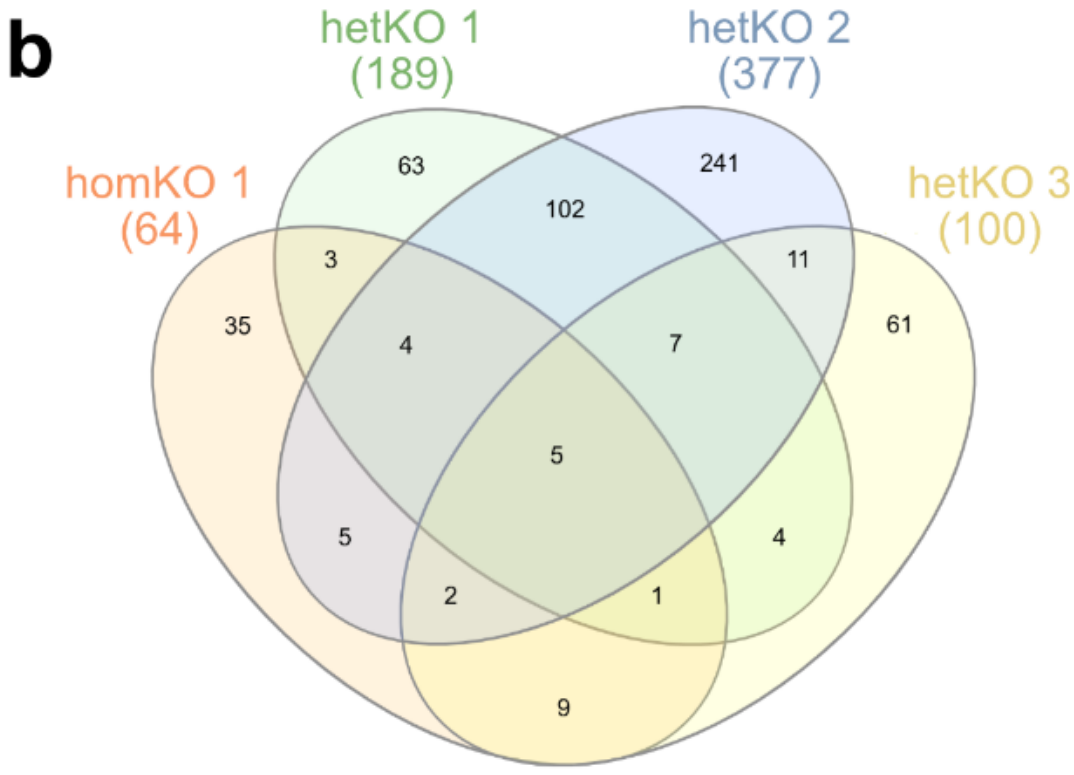

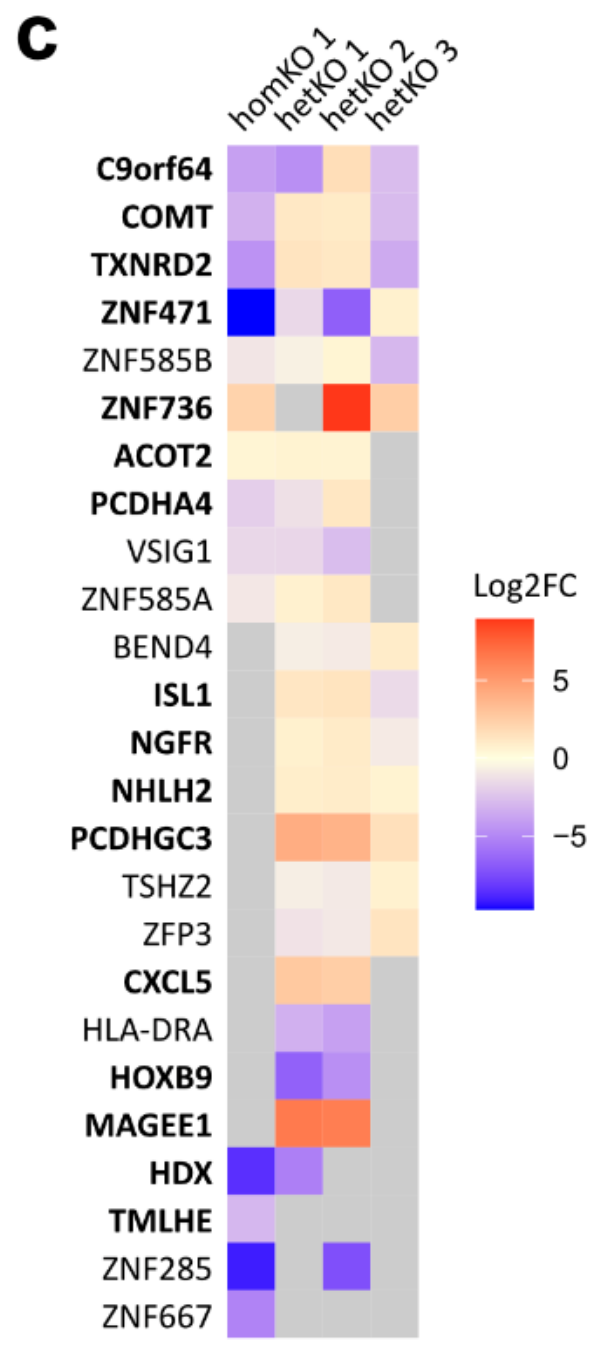

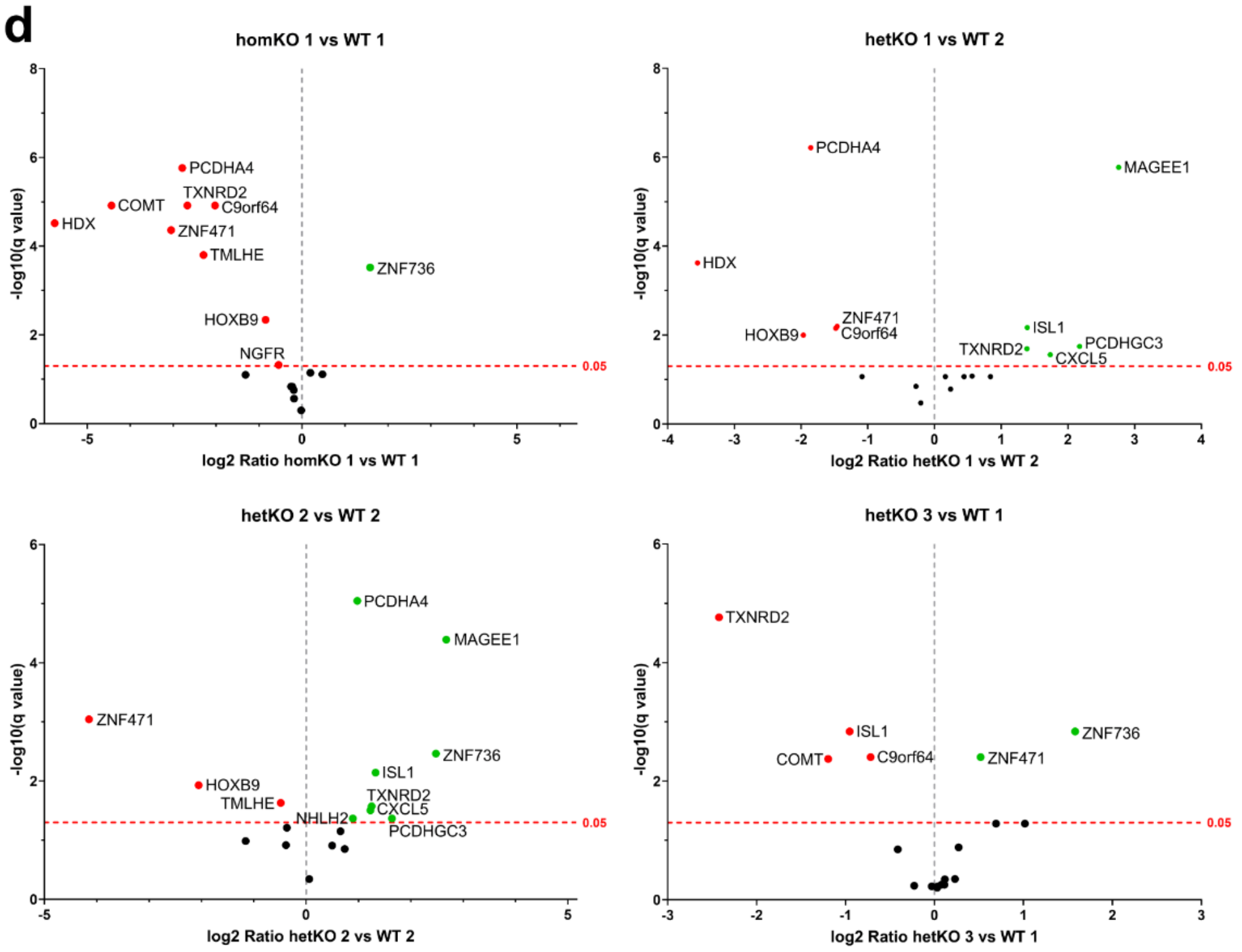

Supplementary Figure 23. Differential expressed protein coding genes in *KCNQ1*-KO neurons. a Table with the number of significantly protein-coding DEGs (FDR < 0.05) identified by whole transcriptome analysis. **b** Venn diagram of significantly differentially expressed protein coding genes in neurons. **c** Heatmap of selected protein coding genes. Five protein-coding genes (*C9orf64*, *COMT*, *TXNRD2*, *ZNF471*, and *ZNF585B*) were consistently differentially expressed across all four *KCNQ1*-KO neuronal

lines compared to WT controls. *C9orf64* encodes a protein involved in tRNA-guanine transglycosylation, while *COMT* encodes catechol-O-methyltransferase. *TXNRD2* regulates mitochondrial redox homeostasis and reactive oxygen species levels. The zinc finger genes (*ZNF471, ZNF585B*) encode transcriptional regulators. Moreover, in three KO cell lines overlapping differential expression was seen for genes with a role in cell adhesion (*PCDHA4*, *PCDHGC3*), neurodevelopment, differentiation, neuronal survival (*NGFR*, *NHLH2*), insulin regulation (*ISL1*), and transcriptional regulation (*ZFP3*, *ZNF585A*, six different *HOX* (e.g. *HOXB9*) genes, and *MAGEE1* which enhances ubiquitine-ligase function. Genes labelled in bold were further followed-up by nCounter analysis. Like in the NSCs, *KCNQ1*, other KCNQ family members (*KCNQ2–5*), and key KCNQ1 interaction partners were not found differentially expressed in any of the neuronal samples analysed. **d** Gene expression analysis with nCounter. Volcano plots showing differential expression of the followed-up genes.

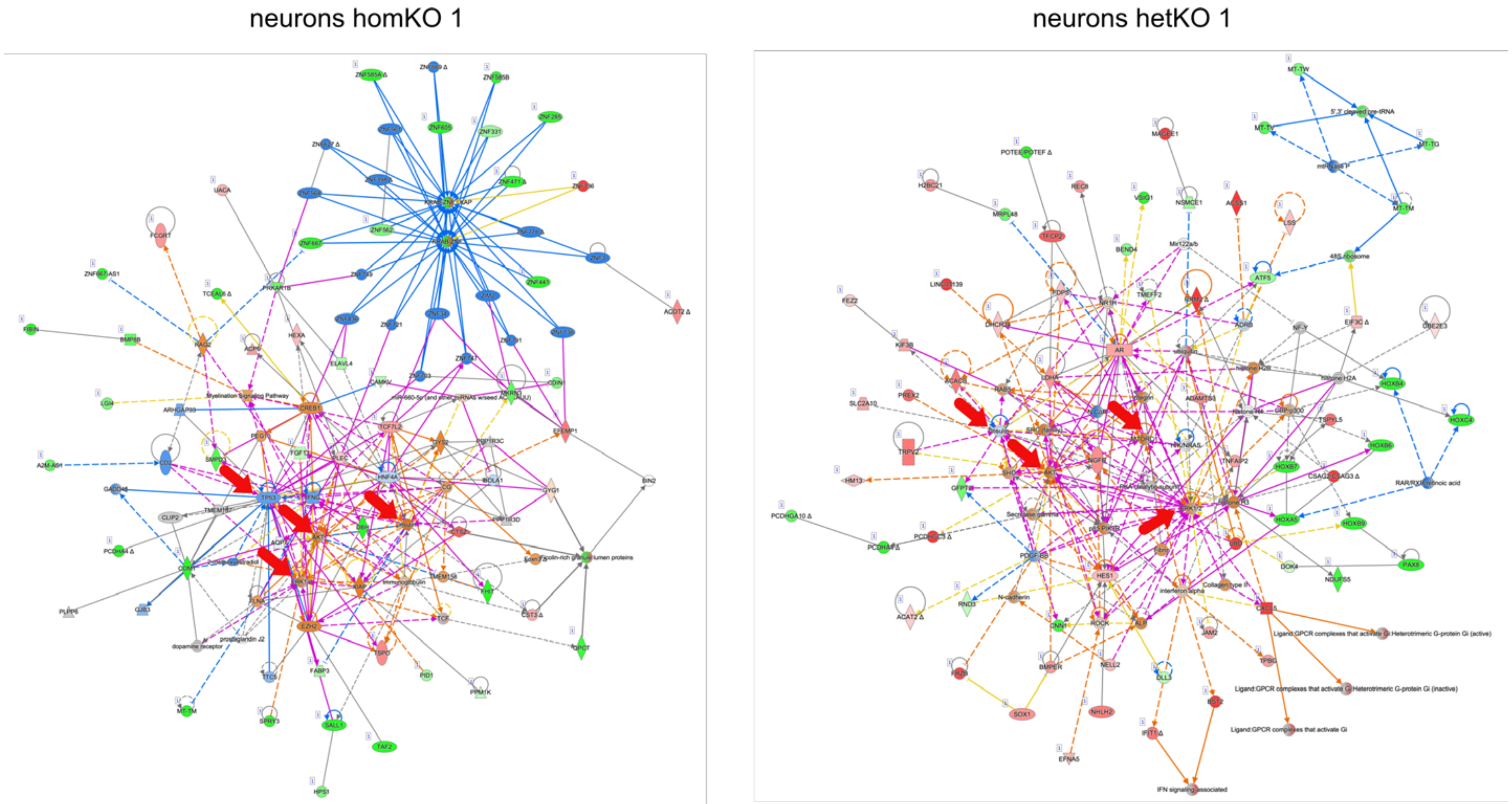

Supplementary Figure 24. Network enrichment analyses of transcriptomic data in homKO 1 and hetKO 1 neurons. The three most significantly enriched networks for all DEGs were merged (IPA). The network enrichment analyses point towards an involvement of insulin signalling (insulin, AKT, ERK) in homKO 1 and hetKO 1 neurons and TP53 in homKO 1 neurons (indicated by red arrows). Genes: green: downregulated in expression, red: upregulated in expression, blue: predicted to be inhibited, orange: predicted to be activated, grey: differentially expressed but not significantly, white: not present; arrows: purple: overlap between networks, orange: predicted positive effect, blue: predicted negative effect, yellow: inconsistent/unpredicted effect, grey: no predicted effect.

neurons hetKO 2

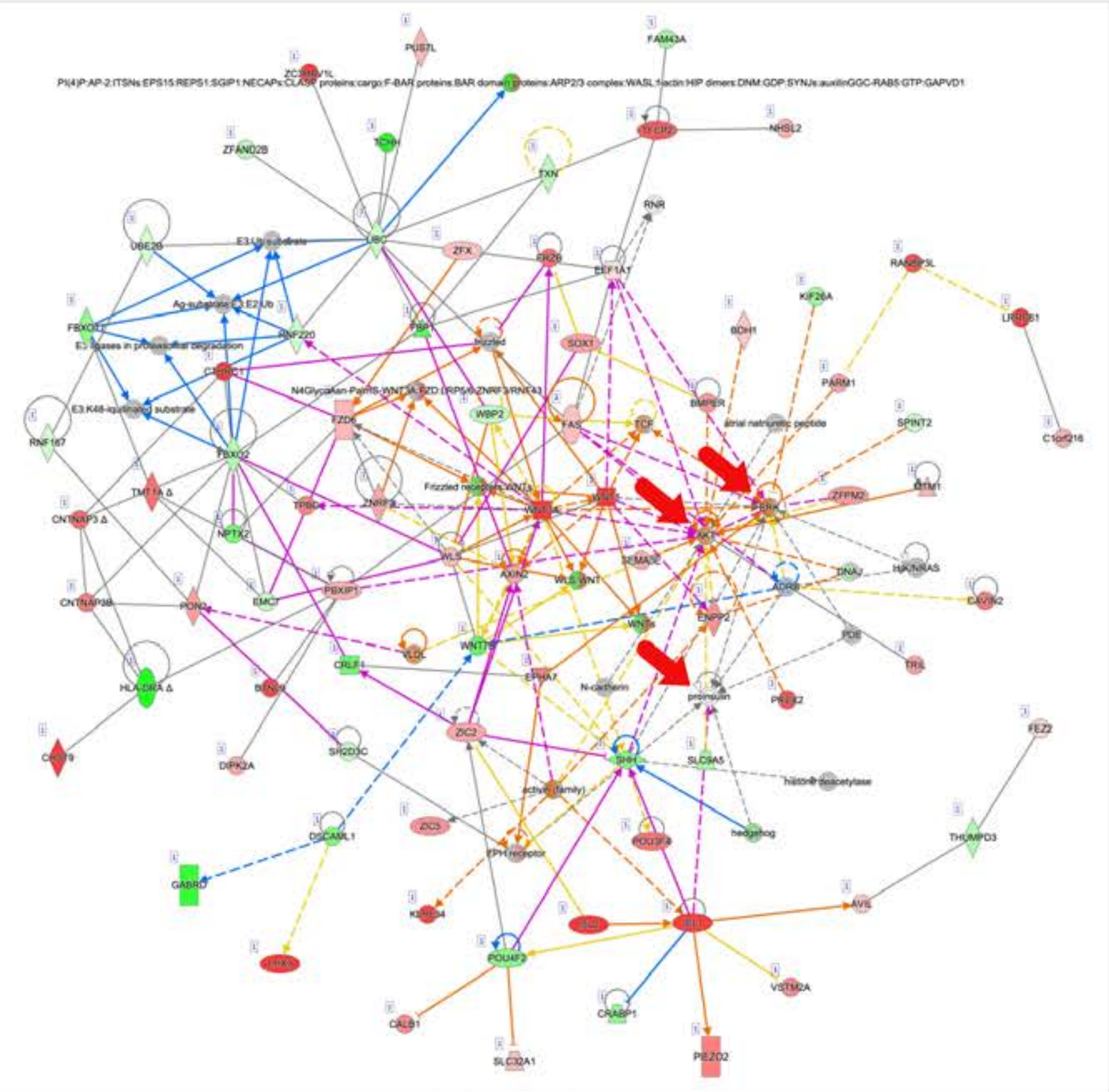

neurons hetKO 3

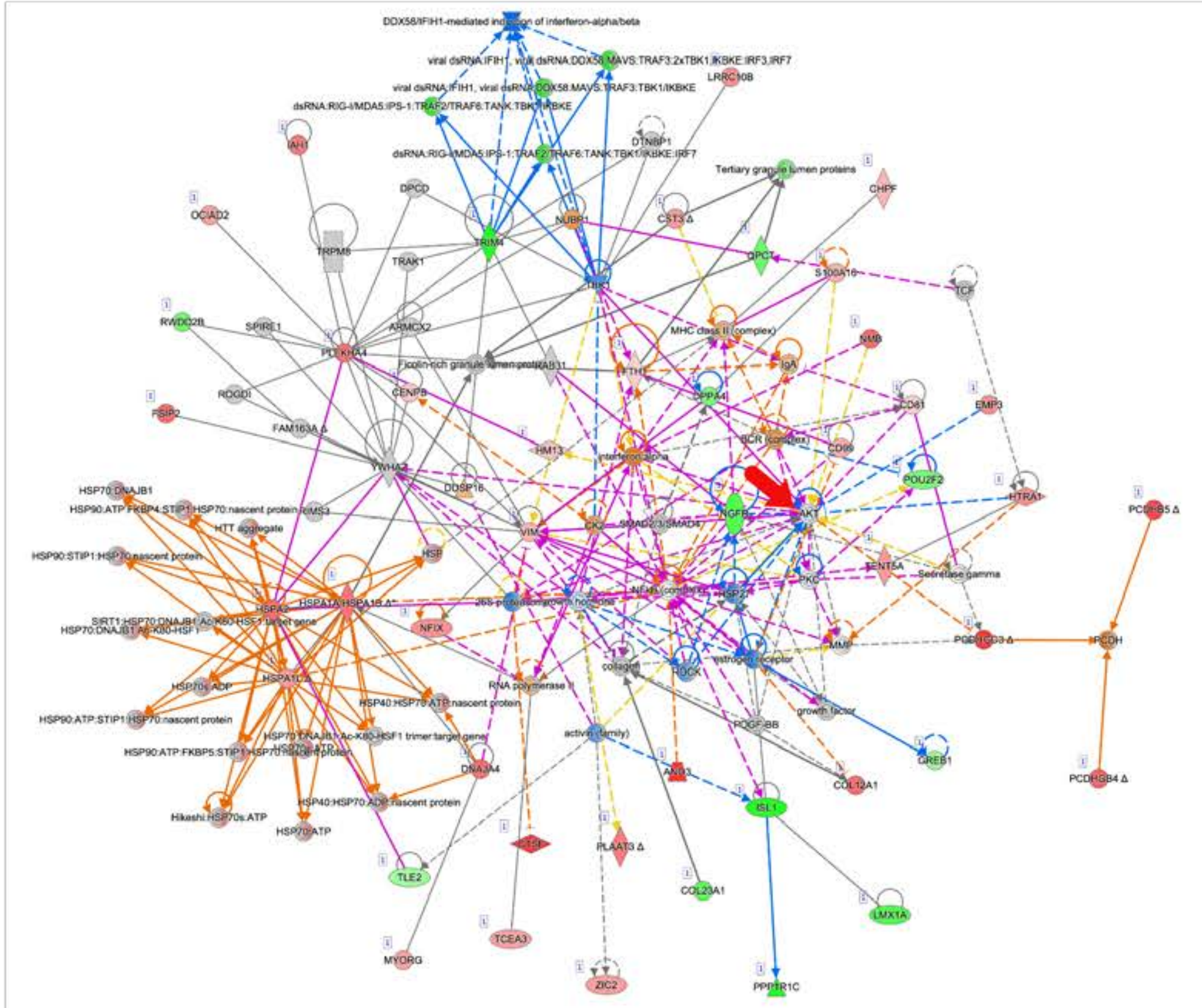

Supplementary Figure 25. Network enrichment analysis of transcriptomic data in hetKO 2 and hetKO 3 neurons. The three most significantly enriched networks for all DEGs were merged (IPA). The network enrichment analyses point towards an involvement of insulin signaling (insulin, AKT, ERK) in hetKO 2 neurons, which is comparable to what we observed in homKO 1 and hetKO 1 neurons. For hetKO 3, only AKT was found as a gene overlapping with the analyses for the other KO cell lines (indicated by red arrows). Genes: green: downregulated in expression, red: upregulated in expression, blue: predicted to be inhibited, orange: predicted to be activated, grey: differentially expressed but not significantly, white: not present; arrows: purple: overlap between networks, orange: predicted positive effect, blue: predicted negative effect, yellow: inconsistent/unpredicted effect, grey: no predicted effect.

a

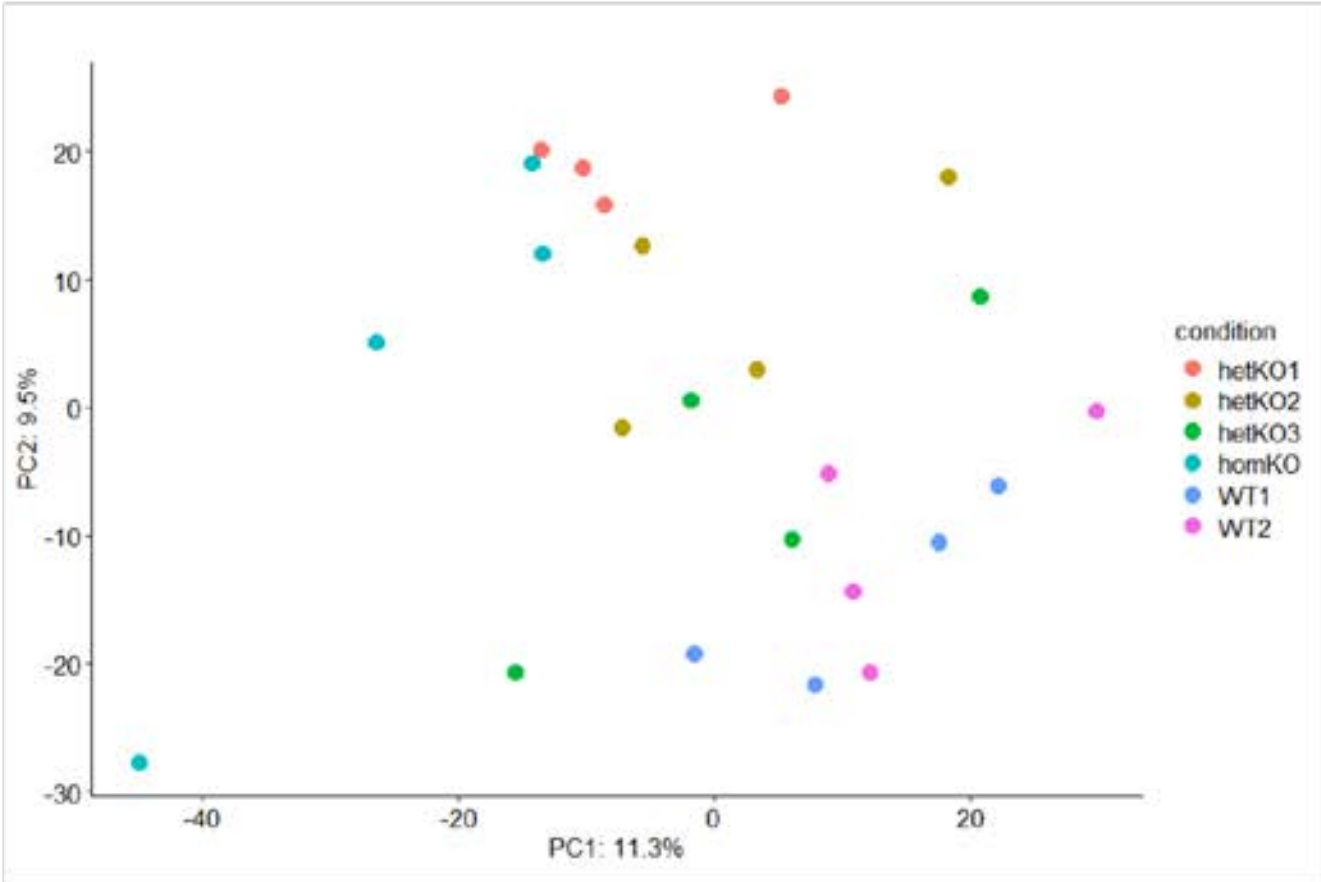

b

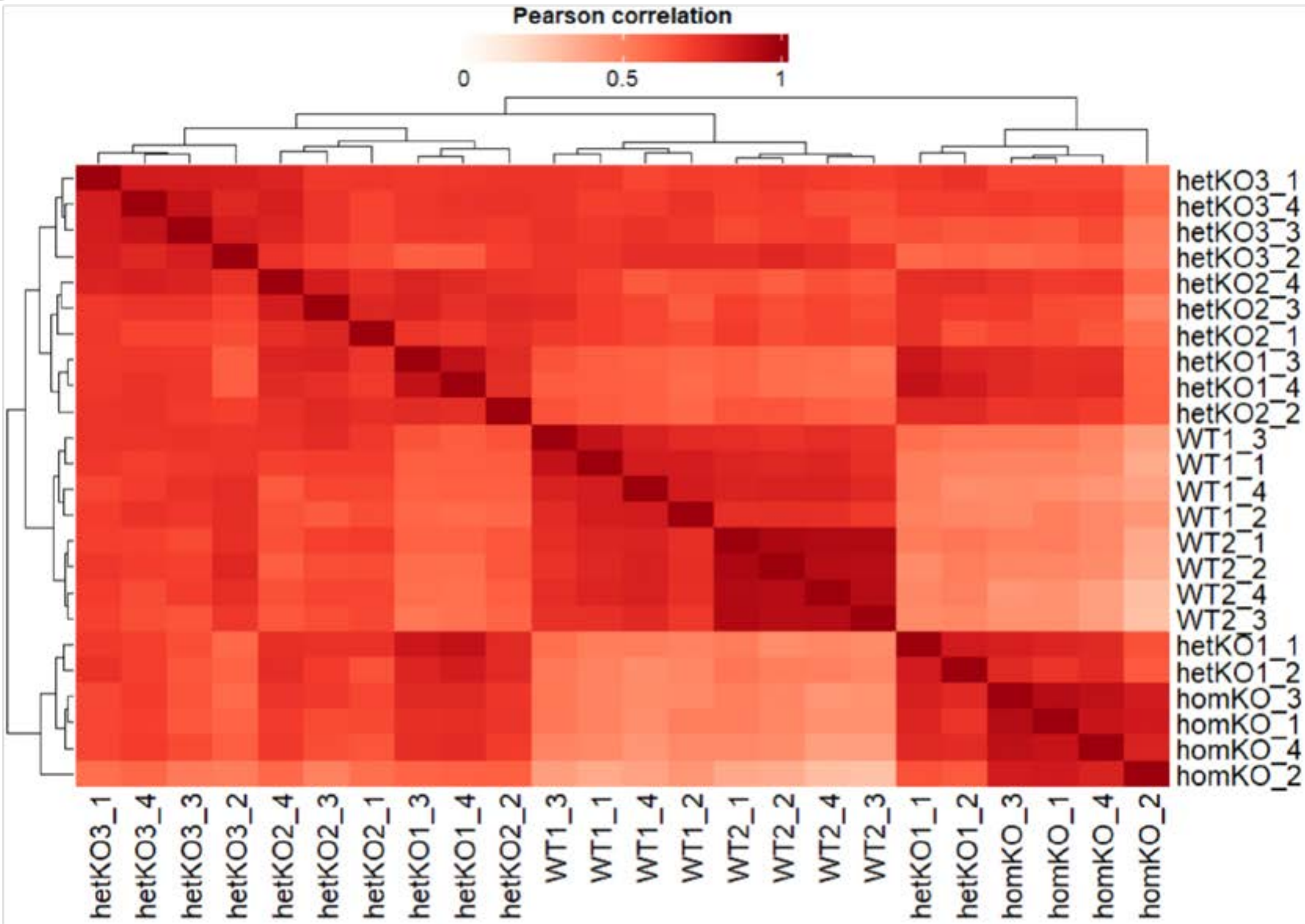

Supplementary Figure 26. Quality assessment of proteomic data from neuronal cell lines. a PCA of proteomic data from WT 1, WT 2, homKO 1, hetKO 1, hetKO 2, and hetKO 3 neurons. PC1 and PC2 explain 11.3% and 9.5% of variance. In the PCA plot WT 1 and WT 2 are separated from homKO 1, hetKO 1, and hetKO 2. HetKO 3 resides between the WT and the other KO lines. **b** Heatmap displaying pairwise Pearson correlation coefficients (based on log_2-transformed protein intensities) across all biological replicates with the dendrogram reflecting hierarchical clustering based on correlation distances. The correlation matrix highlights clear grouping by genotype. One replicate of hetKO 1 clusters with homKO 1, suggesting shared proteomic features likely due to the deleted allele in hetKO 1 being on the active allele. Three replicates of hetKO 1 group closely with hetKO 2, indicating strong similarity in protein expression profiles. HetKO 3 forms a distinct sub-cluster but remains closer to other hetKO samples than to WT samples. WT and all hetKO replicates exhibit higher inter-sample similarity compared to homKO 1, which shows the most divergent proteomic profile, indicating the strongest impact of full *KCNQ1* deletion on protein expression.

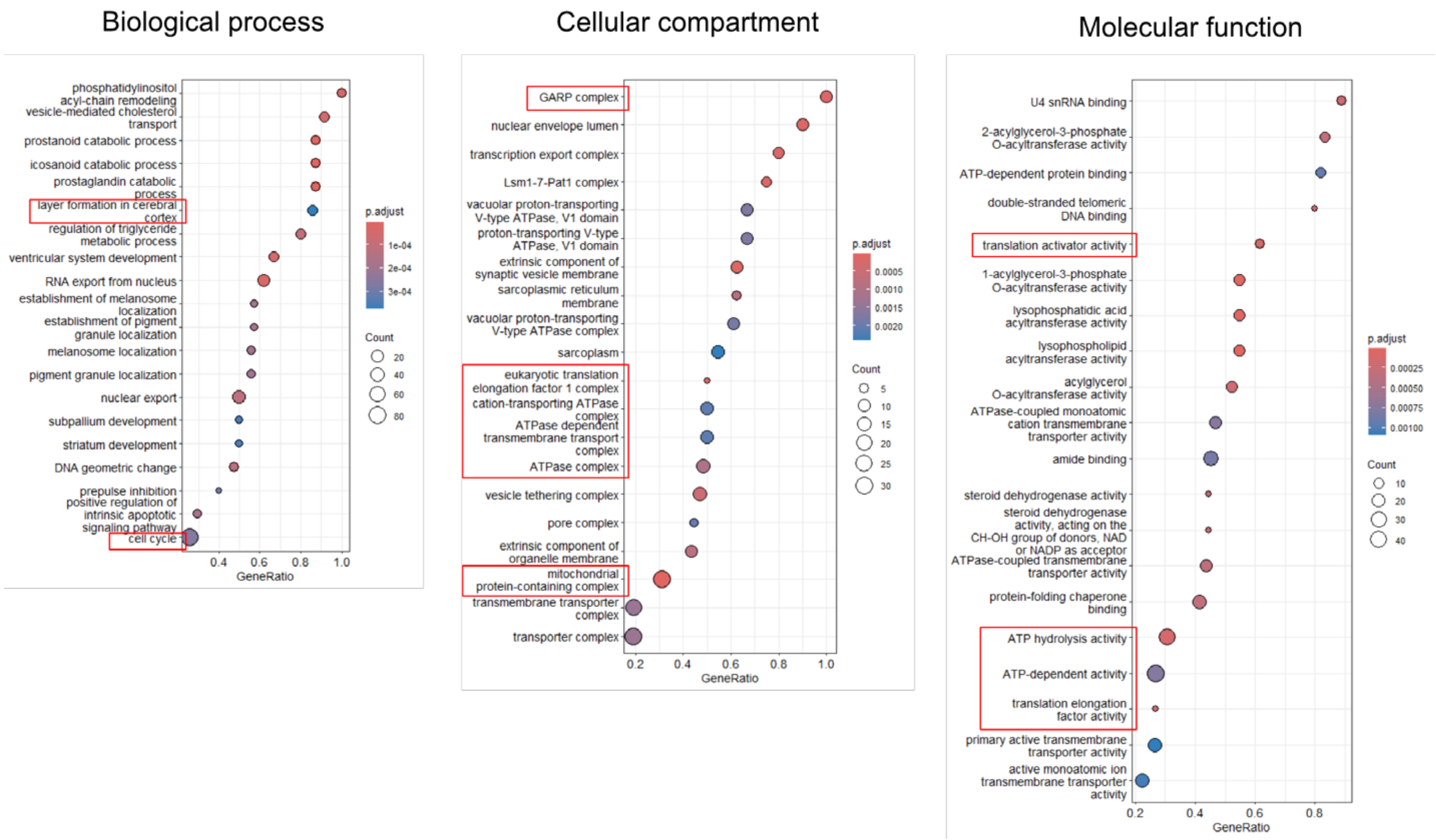

Supplementary Figure 27. GSEA comparing homKO 1 and WT 1 neurons. All nominal significant proteins and their log2fold changes were taken for the analysis. The top 20 affected biological processes, cellular compartments, and molecular functions are shown. Most interesting findings were labelled in red boxes and are summarized in the main Table 1.

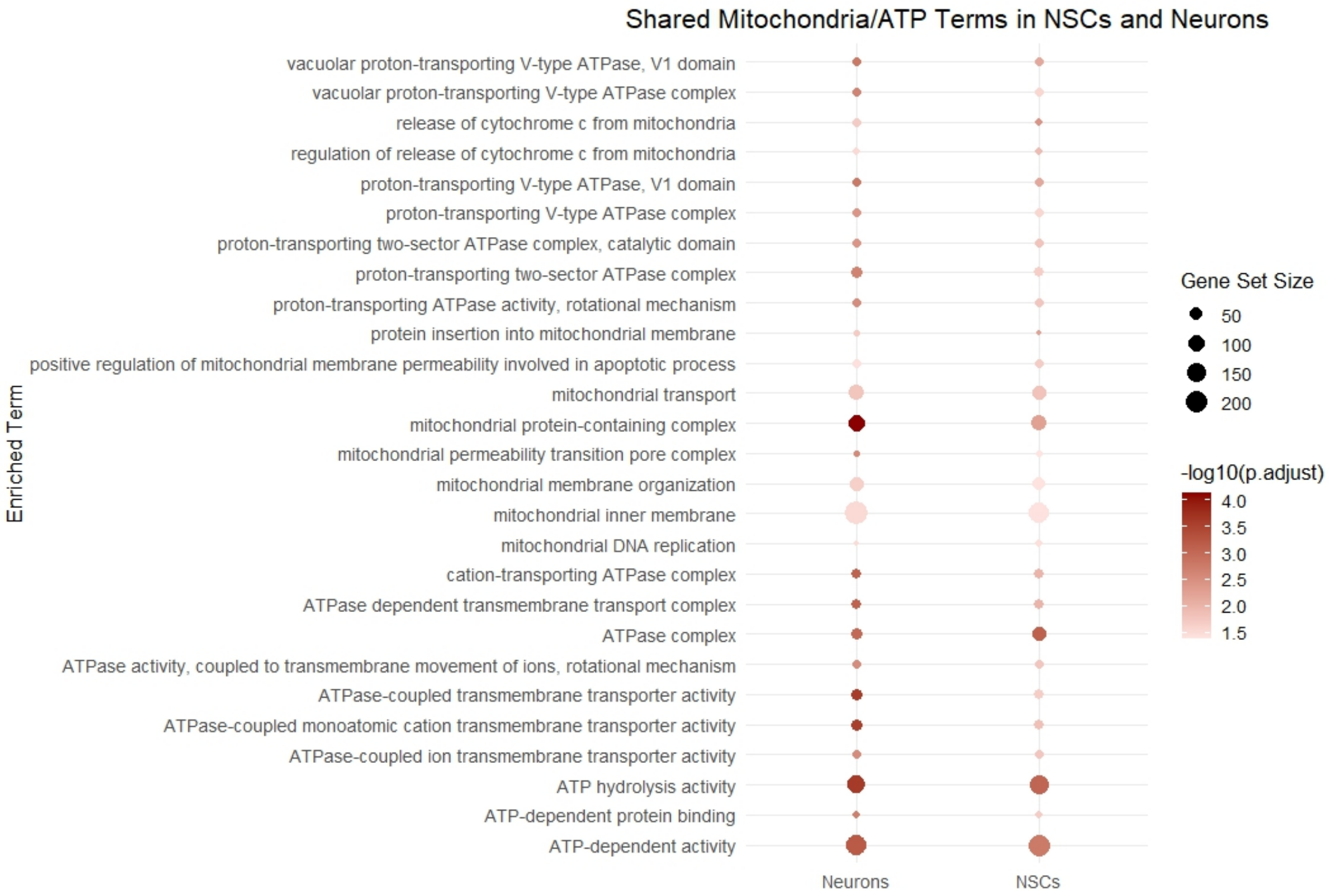

Supplementary Figure 28. GSEA focusing on mitochondria and ATP-related GO terms in homKO 1 / WT 1 NSCs and neurons. Several mitochondrial (mt) functional aspects showed enrichment of downregulated proteins in both cell types, including cytochrome c release, protein insertion into the mt membrane, mt transport, regulation of mt membrane permeability in apoptosis, and mt membrane organization (see also Suppl. Table 2 Tab 3 and 5). Mt DNA replication was found enriched among the upregulated proteins. Overlapping cellular compartments (significant GO terms) are: mt protein-containing complex, the inner membrane, and the permeability transition pore complex. Several ATP-related processes were found enriched among the upregulated proteins such as ATPase activity, ATP-dependent activity, and among the downregulated proteins such as ATP-dependent protein binding.

Supplementary Materials + Methods

Neuronal differentiation protocol 2

This differentiation protocol was only used for one set of neurite outgrowth measurements. Neurons were generated according to the protocol by Qi et al. (PMID: 28112759), adapted by Dr. Magdalena Laugsch and colleagues. IPSCs were seeded in a 6-well on Geltrex with 250.000 cells in mTeSR Plus medium. 24h after splitting, medium was changed to differentiation medium including different small molecules. Every second day, media with small molecules was changed (see details in table below). On day 8, cells reached an NSC-like state and were split in a 24-well on Ornithin-Geltrex coating with 380.000 cells in NDM + PD0325901 + SU5402 + DAPT + Chir99021. Cells were placed into the Incucyte system immediately after splitting and measured for 48h.

Overview of medium composition and small molecules for day 0-7 of protocol 2.

Day of differentiation	Material	Concentration
Day 0 + Day 1	DMEM F12 + Glutamax	84%
	Knockout Serum Replacement	15%
	MEM-NAC	1%
	β-Mercaptoethanol	100 µM
	LDN193189	250 nM
	SB431542	10 µM
	XAV939	5 µM
Day 2 + Day 3	DMEM F12 + Glutamax	84%
	Knockout Serum Replacement	15%
	MEM-NAC	1%
	β-Mercaptoethanol	100 µM
	LDN193189	250 nM
	SB431542	10 µM
	XAV939	5 µM
	PD0325901	1 µM
	SU5402	5 µM
	DAPT	10 µM
Day 4 + Day 5	DMEM F12 + Glutamax	72%
	Knockout Serum Replacement	10%
	MEM-NAC	1%
	β-Mercaptoethanol	66.6 µM
	Neurobasal	16.60%
	Glutamax	0.16%
	N2	0.33%
	B27	0.66%
	LDN193189	250 nM
	SB431542	10 µM
	XAV939	5 µM
	PD0325901	1 µM
	SU5402	5 µM
	DAPT	10 µM
Day 6 + Day 7	DMEM F12 + Glutamax	51%

	Knockout Serum Replacement	5%
	MEM-NAC	1%
	β-Mercaptoethanol	33.3 µM
	Neurobasal	33.3%
	Glutamax	0.33%
	N2	0.66%
	B27	1.33%
	PD0325901	1 µM
	SU5402	5 µM
	DAPT	10 µM

Primer table

Experiment	Target	Sequence
Genome editing	gRNA 1 (chr11: 2,571,242-2,571,261; GRCh38/hg38)	GCAGGGTGTATGCTCTTCC
	gRNA 2 (chr11: 2,571,251-2,571,270; GRCh38/hg38)	GTTCAGGTACCCCTGCGCCC
	gRNA 3 (chr11: 2,572,227-2,572,246; GRCh38/hg38)	ATACACCCTGCTCTCGTCTG
	gRNA 4 (2,572,450-2,572,469; GRCh38/hg38)	CCCATCTGAGTGCAGCCCTC
KO screening	KCNQ1 intron 2 F	CCCTTTCTGGCCACTTGCA
	KCNQ1 intron 5 R	TTGCCCAGCTCTCTTTCCTG
	KCNQ1 exon 4 F	GTGTATGCTCTTCCCTGGGG
	KCNQ1 exon 4 R	GTGGATGGGGCGTGAGAC
	KCNQ1 exon 5 F	GGACACCCATGCCATCGG
	KCNQ1 exon 5 R	CAAGCTGTCCTAGTGTGGGC
Assignment of deletion and allele	KCNQ1 WT allele F	GTTGAGCACTGACTGGAGCA
	KCNQ1 WT allele R	GCTGGGTATATGTCCATCCCG
	KCNQ1 KO allele F	CGATGACCAAACAAAGGCGG
	KCNQ1 KO allele R	CCTCACAACTCCGGTAAGTGG
	SNP 1 F	GTTGAGCACTGACTGGAGCA
	SNP 1 R	CGGACAGGTGCATACTGGAG
	SNP 2 F	CCCTGCCATCTCCAGTATGC
	SNP 2 R	CACCATCCGGCATGTGTTTG
Off-target analysis – gRNA 1	RPS19 F	GGCCTTCCCAGGTCAAACAG

	RPS19 R	AAGGACCATTCAGCCTGTGC
	ELAV4 F	AGCACTCTCCCACGTCCA
	ELAV4 R	ATGCACTTAGTGGGTTCAGCA
	NES F	TTCCAGGATCGGGGTGTACG
	NES R	AGTTTGGAGGCAAAGAGGGT
	CLDN9 F	TGAATGTGGAACCGGTGAGTC
	CLDN9 R	GGGGCTCAGATTTCACAGGG
Off-target analysis – gRNA 2	PCBD1 F	AGGGAAAAGGTCTAAATTCCTG
	PCBD1 R	AGAATAGCAGCTGGCCAGTAC
Off-target analysis – gRNA 3	RAD51B F	AAAGCTGGTGAAAGTCAGGG
	RAD51B R	AGTTTGGAGGCAAAGAGGGT
	AC145676.2 F	TCAGTGTGGGGTCAACAAGC
	AC145676.2 R	GGCGTCTGTTCAGTCAGGTC
	ADAM15 F	CAAGCAACACTCTGCGGACCTGC
	ADAM15 R	ACAGCTTGACAGATTGCGCC
	TCEB1P20 F	AGGGAGTGCTGGAGCTAGTA
	TCEB1P20 R	CAAGTACTGCACGGCTTGTC
	MCF2L2 F	AGGGAGTGCTGGAGCTAGTA
	MCF2L2 R	CAAGTACTGCACGGCTTGTC
	LARGE F	TTGCTCTTCCGTATCTACCACA
	LARGE R	GAGGCCAGAGAGCTTTCAAGT
	PKHD1 F	TGCCTACTCCCAAGCTCAGAA
	PKHD1 R	TGCAACTTTGTCCCTCGATGT
Off-target analysis – gRNA 4	PRR5L F	AAACGTGTCACCAGCCCG
	PRR5L R	ACCCACCACTCACCTCCTT
	THEM6 F	CGTAGCTTAGCTGGGTGTCC
	THEM6 R	GTCAGGATGCCAATGCCACT
Trilineage differentiation	SOX17 F	ACGCCGAGTTGAGCAAGA
	SOX17 R	TCTGCCTCCTCCACGAAG
	GATA6 F	ACCACCTTATGGCGCAGAAA
	GATA6 R	ATAGCAAGTGGTCTGGGCAC
	WNT3 F	ATCTACGACGTGCACACCTG
	WNT3 R	TGCTTCCCATGAGACTTCGC
	ACTA2 F	CCTATCCCCGGGACTAAGAC
	ACTA2 R	AGGCAGTGCTGTCCTCTTCT
	TBXT F	GCTGTGACAGGTACCCAACC
	TBXT R	CATGCAGGTGAGTTGTCAGAA
	FOXF1 F	CAGCCTCTCCACGCACTC
	FOXF1 R	CCTTTCGGTCACACATGCT
	DESa F	GTGAAGATGGCCCTGGATGT
	DESa R	CGGCTGGTTTCTCGGAAGTT
	NCAM1 F	AGACGCAGCCAGTCCAAG
	NCAM1 R	TGCTTGATCAGGTTCACTTTAATAGA
	PAX6 F	TCAGAGCCCCATATTCGAGC
	PAX6 R	CAAAGACACCACCGAGCTGA
	NES F	CAGCGTTGGAACAGAGGTTGG

	NES R	TGGCACAGGTGTCTCAAGGGTAG
Characterization neurons	MAP2 F	CGAAGCGCCAATGGATTCC
	MAP2 R	TGAACTATCCTTGCAGACACCT
	SYP F	CCAATCAGATGTAGTCTGGTCAGT
	SYP R	AGGGGTGGAGACCTAGGGTA
	DLG4 F	TCACAACCTCTTATTCCCAGCA
	DLG4 R	CATGGCTGTGGGGTAGTAGTCG
	GRIA2 F	ACTGACACCCCACATCGAC
	GRIA2 R	TCGAAAACTGGGAGCAGAAA
	GABRB3 F	TGAGCTCCCGCAGTTCTC
	GABRB3 R	CAGTGACAGTCGAGGATAGGC
	RELN F	GCCAAAGGACTTCACACAAGC
	RELN R	CATGTAATTTTGTTTGCGAGTGC
	CUX2 F	AAGGAGATCGAGTCGCAGAA
	CUX2 R	CTCCAGGATGCTCTTGATGG
	POU3F2 F	TTGTGTTGCCCCTTCTTCGT
	POU3F2 R	TTGCCTTCGATAAAGCGGGT
	FOXP2 F	GCAGCAGAGATGGAAGATCA
	FOXP2 R	AGTTGTCTTGCTGCCTGGAG
	GFAP F	GCACGCAGTATGAGGCAATG
	GFAP R	TAGTCGTTGGCTTCGTGCTT
	INSR F	CGAGAAGACCATCGACTCGG
	INSR R	GACACCAGAGCGTAGGATCG
Expression of KCNQ1	KCNQ1 F	ATCTCCTTCTTTGCGCTCCC
	KCNQ1 R	CCTCCATGCGGTCTGAATGA
Expression of KCNQ1OT1	KCNQ1OT1 F	CTGGCCCTGGAGATTATTCA
	KCNQ1OT1 R	AGCCCTAAAATGGAGGAATAGG
Reference genes	GAPDH F	CTGGGCTACACTGAGCACC
	GAPDH R	AAGTGGTCGTTGAGGGCAATG
	SDHA F	TGGGAACAAGAGGGCATCTG
	SDHA R	CCACCACTGCATCAAATTCATG
RT_PCR	KCNQ1 exon 2-7F	GTCTGCCTCATCTTCAGCGT
	KCNQ1 exon 2-7R	CAGAGAAGCAGGAGGCGATG
Mitochondria copy number and deletion	ND4 F	GCTCACTCACCCACCACATT
	ND4 R	TCGGGGTTGAGGGATAGGAG
	ND2 F	ATCCACCCTCCTCTCCCTAG
	ND2 R	GGTGGGGATGATGAGGCTAT
	CO1 F	GCCCACTTCCACTATGTCCT
	CO1 R	GCGTAGGTTTGGTCTAGGGT
	β2M F	TCCACCTCTTGATGGGGCTA
	β2M R	GAGTACCAGGCCACCTTGAC
RNA seq follow-up	COMT F	CCCAGCTGAAGAAGAAGTATGAT
	COMT R	CCGCAGCAGGCCACATT

	PCDHA4 F	GCCCCAGTTTACCTGACTCT
	PCDHA4 R	GAGGCAGAGTAACGCCAGTC
	ZNF471 F	GAGCCTTGGGAGATGACGAG
	ZNF471 R	TCATACATGAACTGCTTCAGAGGT
	HDX F	CTGTGCCTTGGATTACAGGGT
	HDX R	AAGGGTGGCTAAGTCTCGGT
	TMLHE F	GGATCGGCACACTGACACTA
	TMLHE R	CAGTGTCCTGCCACCAGTTC
Mouse primer	Kcnq1 F	CCACATCAGCTATCAGGGGTA
	Kcnq1 R	TGAAGACTACAGAGCCCAGG
	Hprt F	TCCTCCTCAGACCGCTTTT
	Hprt R	CCTGGTTCATCATCGCTAATC
	Sdha F	CATGCCAGGGAAGATTACAAA
	Sdha R	GTTCCCCAAACGGCTTCT

Antibody table

Antibody	Company	Cat. No	Concentration
Anti-KCNQ1	abcam	ab84819	1:500
Anti-AKT	Cell Signaling Technologies	9272	1:1000
Anti-p44/42 MAPK (ERK1/2)	Cell Signaling Technologies	4696	1:1000
Anti-Phospho-p44/42 MAPK (Erk1/2) (Thr202/Tyr204)	Cell Signaling Technologies	4370	1:1000
Anti-ATP5A	Abcam	Ab14748	1:1000
Anti-GAPDH	NovusBio	NB300-328SS	1:15000
Anti-Rabbit, IRDye800CW	LI-COR	926-32213	1:15000
Anti-Mouse, IRDye800CW	LI-COR	926-32212	1:15000
Anti-Rabbit, IRDye680CW	LI-COR	926-32223	1:15000
Anti-Mouse, IRDye680CW	LI-COR	926-32222	1:15000
Anti-Nanog	NovusBio	NB100-588	1:100
Anti-OCT4	Abcam	ab181557	1:200
Anti-Nestin	Abcam	ab22035	1:400
Anti-PAX6	BioLedgend	901301	1:100
Anti-SOX2	Abcam	ab97959	1:100
Anti-mouse Alexa Fluor 594	Invitrogen	A11032	1:500

Anti-rabbit Alexa Fluor 488	Invitrogen	A11008	1:500

TagSet probe design for nCounter analysis

Gene	Accession number	Sequence probe A	Sequence probe B
C9orf64	NM_032307.3	GAAAAGAGCCTCCAAACTTC TCCAGCAGAATTTTCCCGGTT TCATTGAGACCAATTTGGTTT TACTCCCCTCGATTATGCGGAGT	CGAAAGCCATGACCTCCGATCACTCTGC ATTAACTTCTGCGCACTATTCTCACTTTC TCGGACGCAGTTGA
COMT	NM_000754.3	GCAGCAGGGGAATGGCAGTTGA GAAGGATGTACTAAGTCGAGTT ATATCTCTTTCGGGTTATATCTAT CATTTACTTGACACCCT	CGAAAGCCATGACCTCCGATCACTCCGT TAGCGTCCGTCAAGGGGAGCTTTGAAT GTTTGGTGCCCAAGTCAAGG
TXNRD2	NM_006440.3	GCAACGCCGCAAACCGTGTGCT CGTCAACAAAGCTGGCTTTGAT GTTAAACAACAGCCACTTTTTTT CCAAATTTTGCAAGAGCC	CGAAAGCCATGACCTCCGATCACTCTAG CAATGATGATGTGATCGGCTGACAGCA GAATCTCTTTCCCACCTTTG
ZNF471	NM_020813.2	GCATCTTGTCTGAGGACATTTG CACATTCTTTACATTGTTAAGA CCGCTCCACCGTGTGGACGGC AACTCAGAGATAACGCATAT	CGAAAGCCATGACCTCCGATCACTCGGT GAGGAAGAGCTGGCTGCCTGAAATGAA CTCACTAATGAGCAAGGTGT
ZNF736	NM_001170905.3	TACCACCACCAGCGCTCTTTG GTTGATGGAGTTTTGAAACC TCTTTGGGACCTGGAGTTTAT GTATTGCCAACGAGTTTGTCTTT	CGAAAGCCATGACCTCCGATCACTCCCG TTTCATTCCTTCTCACTGGAAAGTACTTC CCAATGAGGGTCTCCAGC
ACOT2	NM_006821.4	GACGCTCGAAGGACAGCAGG AGATGAGGCCATTTTGAATTC AGACCTGAGCAGATAAGGTTG TTATTGTGGAGGATGTTACTACA	CGAAAGCCATGACCTCCGATCACTCAGG AACTGCGCCGAACTCTTCAGGCTCCATTG GTACAGCCGG
PCDHA4	NM_031500.2	ATAACTCTACAATGGCCAGAA AGTGGGAGCTGTCCCTTATCA ATGCCCTCCTTCCTTCCTGTGT TCCAGCTACAAACTTAGAAAC	CGAAAGCCATGACCTCCGATCACTCACT TGAATTCCAAATCTGGGACATTATCGT TGTTGTCTTCTACTTCCACA
ISL1	NM_002202.2	CTTTCCAAGGTGGCTGGTAAC TTTGTACTTCCACTGGGTTAGC CTGTAAGCATAAAATTGGTTTT GCCTTTCAGCAATTCAACTT	CGAAAGCCATGACCTCCGATCACTCCTG AAAAGCAGGCTGATCTATGTCACTCTG CAAGGCGAAGTCGCTCAGTA
NGFR	NM_002507.3	CGAAGTCAATTCCTTCTTGCCG CATTCCCACACTGGCTGGTCAA GACTTGCATGAGGACCC GCAAATTCCT	CGAAAGCCATGACCTCCGATCACTCGT CTAGAGCTGGGAGAAATCCCCACAGG TCACAGT
NHLH2	NM_005599.3	CGCCCAGGGACTCCGGATCCGA GTGCGCCGAGCTGGGATGGTCC GAATCTCTTTCGTTGGGACGCTT GAAGCGCAAGTAGAAAAC	CGAAAGCCATGACCTCCGATCACTCTC CAGGTCCGACACGCTGCCGAGCACCT TGGTGTCCGTGC
PCDHGC3	NM_032403.3	CGCCGAGTCCGACATGGCTTTC TGGATGCCGTTGATTTGCCCAG CAGACCTGCAATATCAAAGTTA TAAGCGCGT	CGAAAGCCATGACCTCCGATCACTCTGA GATTTCGCTGAGAAACTTTCAGCGGGT TAGCGCTTGGGCGCTGGG

CXCL5	NM_002994.3	TTGGGATGAACTCCTTGCGTGGT CTGTAAACAAACGCAACGCAGCT CTCTCCTGCCAATGCACTCGATCT TGTCATTTTTTTGCG	CGAAAGCCATGACCTCCGATCACTCCC TTGGAGCACTGTGGGCCTATGGCGAA CACTTGCAGATTACTGATCATT
HOXB9	NM_024017.5	AGACAGCACTGGCTTTGCAGTCG TCACATAACTAAGAGTGAGATGG GGAACGATTGCTGCATTCCGCTC AACGCTTGAGGAAGTA	CGAAAGCCATGACCTCCGATCACTCTA AAGGACTTGGAAGAGAGACTCCCTTC CCCATTCACTTGAATACATCCC
MAGEE1	NM_020932.2	GAATCCATAAGCAGAAATAACTG CACCAGCTTCCTTGCCTTGCTCTC CAACTGAGGCTGTTAAAGCTGTA GCAACTCTTCCACGA	CGAAAGCCATGACCTCCGATCACTC CTCGGCCAATGTAGTACAGAATTCC TTTCTTTGGTATAGGCAGCTTAGTT
HDX	NM_001177478.1	TGTACTTTGGTTCTCAGCTCG AAGGGAACTGCCACTTTCATA AAATCTCGCTAGGACGCAAAT CACTTGAAGAAGTGAAAGCGAG	CGAAAGCCATGACCTCCGATCACT CATATTCACCATTTGTGAATTTGGCA TATTTCTTCCTGGTCCGGGCAAGGT
TMLHE	XM_011531182.4	TTCTAAGAAGCTCTGGCAATC TACCGATGGAACTTGGGCTTG CTGGTAGACCACGCGATGACG TTCGTCAAGAGTCGCATAATCT	CGAAAGCCATGACCTCCGATCACTC ATTCCATAGAGCAGAAAGTTTTGCA GAAACTTCTTCAGTCCCTCGTTGGT
GAPDH (HKG)	NM_001256799.1	GCTCCTGGAAGATGGTGATGGG ATTTCCATTGATGACAAGCTTCC CGTTCCCTCAAGACCTAAGCGAC AGCGTGACCTTGTTTCA	CGAAAGCCATGACCTCCGATCACT CCGCCAGCATCGCCCCACTTGATT TTGGAGGGATCTC
HPRT1 (HKG)	NM_000194.1	TGAGCACACAGAGGGCTACA ATGTGATGGCCTCCCATCTCC TTCATCACACATCCTCTTCTTT TCTTGGTGTTGAGAAGATGCTC	CGAAAGCCATGACCTCCGATCACTC CAGTGCTTTGATGTAATCCAGCAGG TCAGCAAAGAATTTATAGCCCCCCT
HSPD1 (HKG)	NM_002156.4	TCAACATCTTCAGCGATTATG ACCAAAGGCTTACGGTGAGCA TTGGCAATCACAATTCTGCGGG TTAGCAGGAAGGTTAGGGAAC	CGAAAGCCATGACCTCCGATCACTC GAAGACCAACCTTTAGCCTATTCAA GACGAGTGTACTTAGAGCTTCTCCA
PSMB4 (HKG)	NM_002796.2	CGGATCCATGAAGGAATCGGGA GTGGACGGAATGCGGTAAAACT GTCCTGCTGTTGAGATTATTGAG CTTCATCATGACCAGAAG	CGAAAGCCATGACCTCCGATCACTCTT CTGGGTCCGCGTGATTGGACCTCTGTA AAGTGCAGACGC
SDHA (HKG)	NM_004168.1	TAAACCCTGCCTCAGAAAGGCCA AATGCAGCTCGCAAGCCTGCCAA AGACGCCTATCTTCCAGTTTGATC GGGAAACT	CGAAAGCCATGACCTCCGATCACTCTG CAACAGTGTGTGACCTGGTAGGAAACA GCTTGGTAACACATGCTGTAT
TBP (HKG)	NM_001172085.1	GCACGAAGTGCAATGGTCTTTAG GTCAAGTTTACAACCAAGATTCAC TGTCGAACCTAACTCCTCG CTACATTCCTATTGTTTTC	CGAAAGCCATGACCTCCGA TCACTCTCCTCATGATTACC GCAGCAAACCGCTTGGGAT TATATTCGGCGTTTCGG
